# Synthesis of New Thiourea-Metal Complexes with Promising Anticancer Properties

**DOI:** 10.3390/molecules26226891

**Published:** 2021-11-16

**Authors:** Guillermo Canudo-Barreras, Lourdes Ortego, Anabel Izaga, Isabel Marzo, Raquel P. Herrera, M. Concepción Gimeno

**Affiliations:** 1 Departamento de Química Inorgánica, Instituto de Síntesis Química y Catálisis Homogénea (ISQCH), CSIC-Universidad de Zaragoza, C/Pedro Cerbuna 12, 50009 Zaragoza, Spain; guillermo@canudo.org (G.C.-B.); lourdesortego@hotmail.com (L.O.); a_izaga_@hotmail.com (A.I.); 2 Laboratorio de Organocatálisis Asimétrica, Departamento de Química Orgánica, Instituto de Síntesis Química y Catálisis Homogénea (ISQCH), CSIC-Universidad de Zaragoza, C/Pedro Cerbuna 12, 50009 Zaragoza, Spain; 3 Departamento de Bioquímica y Biología Celular, Universidad de Zaragoza, C/Pedro Cerbuna 12, 50009 Zaragoza, Spain; imarzo@unizar.es

**Keywords:** biological properties, cancer, cytotoxicity, gold, metal complexes, silver, thiourea

## Abstract

In this work, two thiourea ligands bearing a phosphine group in one arm and in the other a phenyl group (**T2**) or 3,5-di-CF_3_ substituted phenyl ring (**T1**) have been prepared and their coordination to Au and Ag has been studied. A different behavior is observed for gold complexes, a linear geometry with coordination only to the phosphorus atom or an equilibrium between the linear and three-coordinated species is present, whereas for silver complexes the coordination of the ligand as P^S chelate is found. The thiourea ligands and their complexes were explored against different cancer cell lines (HeLa, A549, and Jurkat). The thiourea ligands do not exhibit relevant cytotoxicity in the tested cell lines and the coordination of a metal triggers excellent cytotoxic values in all cases. In general, data showed that gold complexes are more cytotoxic than the silver compounds with **T1**, in particular the complexes [Au**T1**(PPh_3_)]OTf, the bis(thiourea) [Au(**T1**)_2_]OTf and the gold-thiolate species [Au(SR)**T1**]. In contrast, with **T2** better results are obtained with silver species [Ag**T1**(PPh_3_)]OTf and the [Ag(**T1**)_2_]OTf. The role played by the ancillary ligand bound to the metal is important since it strongly affects the cytotoxic activity, being the bis(thiourea) complex the most active species. This study demonstrates that metal complexes derived from thiourea can be biologically active and these compounds are promising leads for further development as potential anticancer agents.

## 1. Introduction

Cancer is one of the leading causes of death in the world [1]. Due to the continued necessity to combat this mortal illness, the number of research groups that has focused their studies on this area is very wide. Antitumor compounds that show cytotoxicity and genotoxicity in these treatments can also alter healthy cells causing very important side effects [2]. Consequently, these undesired effects can limit the effectiveness of the treatments. For this reason, the development of selective antitumor agents has become a great challenge in last decades. 

Metal-based drugs have been widely used for chemotherapeutic treatments in certain types of cancer. For instance, cisplatin and other Pt derivatives have been clinically used in several cancer types [3,4,5]. However, recently, the interest in metal complexes not containing Pt as a metal center has stimulated the possibility of developing new anticancer agents with new modes of action [6,7,8,9]. For example, ruthenium complexes are already being studied and some of them are in clinical trials [10,11].

In this field, one of the best alternatives has been the design and preparation of active gold species, which have gathered increasing attention due to their strong inhibitory growth effect in tumor cells [12,13,14,15,16,17,18,19]. In addition, gold complexes exert their antitumor properties through different targets to those of cisplatin, and thiol containing enzymes mainly in mitochondria, especially thioredoxin reductase and others as for example proteasome, glutathione reductase, or cyclooxygenases, are the main targets of gold compounds [14,16,20]. Our group has a great and long experience in this appealing field of research [21,22,23,24,25,26,27]. 

In this context, numerous gold complexes have been prepared with a wide range of ligands with antitumor activity and some studies show that the gold centers and their ligands are important for the transport of the metal atom to the biological target, thus avoiding collateral effects. For this reason, the use of biologically active ligands is an interesting and challenging task to be developed [28,29,30].

**Figure 1:**
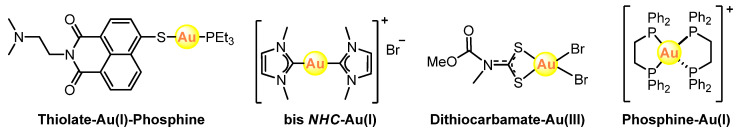
Biologically active gold complexes with interesting ligands.

The stability of gold complexes in the biological environment is an important property to take into account in the development of new gold-based drugs. For this reason, ligands with biological interest have been chosen to achieve an effective interaction with gold atom. Hence, thiolate [31,32], dithiocarbamate [33,34], phosphine [35,36], or *N*-heterocyclic carbene [20,37,38,39,40] ligands, among others, have been at the center of study in the last two decades, showing good biological results (Figure 1).

On the other hand, Ag(I) complexes have also been widely used as antimicrobial [41] and anti-inflammatory [42] agents and have also received special attention for their antitumor activity [26,43,44,45,46,47]. An important advantage of silver compounds as anticancer drugs is the low toxicity exhibited by their complexes [48]. The mechanism of action for which silver compounds exert their antiproliferative effects is probably the interaction with DNA and by binding to thiol containing proteins, thus combining to some extent the mode of action of platinum and gold complexes.

Therefore, the design and synthesis of new Au and Ag species can give access to a class of complexes with interesting biological and pharmacological properties which are different from those of cisplatin derivatives, becoming an interesting alternative in the research of bioinorganic and bioorganometallic medical chemistry.

Moreover, among the antitumor compounds discovered in recent years thiourea derivatives have exhibited potent anticancer properties [49,50,51,52,53,54,55,56,57]. These ligands, due to the presence of hydrogen bonding NH moieties, can favor the formation of such bonds, thus increasing their solubility in an aqueous medium and allowing a better cellular uptake. The great versatility of thioureas is noteworthy, since some of their derivatives have proven to be agents with a great diversity of biological properties, ranging from bactericidal to herbicidal or insecticides [58,59,60,61,62,63].

**Figure 2:**
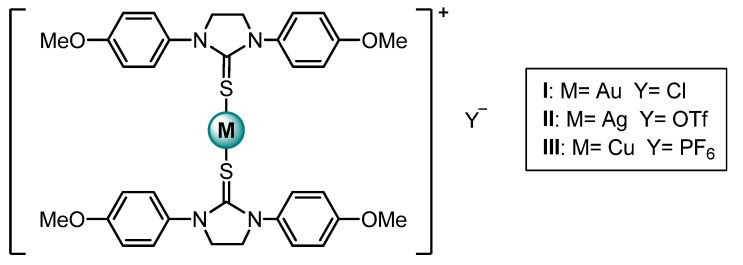
Thiourea-group 11 metal complexes **I–III** with good cytotoxic activity.

In 2010, Che and co-workers pioneered the use of thiourea-group 11 metal complexes with good cytotoxic activity and excellent TrxR inhibition (Figure 2) [64]. In this work the authors concluded that the metal complex considerably improved the results given by thiourea ligand alone. Hence, by varying the thiourea ligands, the resulting metal complexes can lead to a novel alternative in drug development. In particular, it has been demonstrated that the Au(I)–thiourea complex **I** confers specific tight-binding inhibition of TrxR with a potency among the highest reported and exhibits effective suppression of the cellular reductase activity. By variation of the thiourea ligand, metal-thiourea complexes have the prospect to be a new class of metal-based drugs leads.

After this pioneering work, scarce transition metal complexes bearing thiourea derived ligands have been reported and evaluated as potential anticancer drugs. In this context and more recently, only Kollipara and co-workers disclosed a work based on neutral and cationic half-sandwich arene d^6^ metal complexes (Ru, Rh, Ir) containing pyridyl and pyrimidyl thiourea ligands with interesting bonding modes and their cytotoxic activity against different cancer cells [65]. 

Hence, in this manuscript, we report the study concerning a synergistic effect between metals, such as Au and Ag, and thiourea ligands. To the best of our knowledge, this work represents one of the scarce examples centered on thiourea-metal complexes and their antitumor activity.

## 2. Results and Discussion

### 2.1. Synthesis of Thioureas ***T1*** and ***T2*** and Metal Complexes ***C1a–e***

**Scheme 1:**
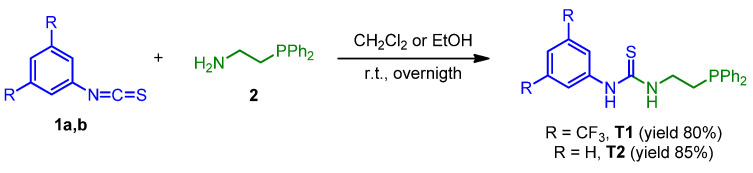
Synthesis of thioureas **T1** and **T2**.

Initially, the synthesis of model thioureas **T1** and **T2** was carried out (Scheme 1). The formation of **T1** and **T2** was performed by reaction of the corresponding isothiocyanate **1a**,**b** with 2-(diphenylphosphino)ethylamine (**2**), in a 1:1 ratio, generating thioureas **T1** (80%) and **T2** (85%) with very good yields. We hypothesized that the synthesis of these two thioureas could facilitate their access to the interior of the cell membrane and thus, their activity could be improved for the metal complexes, especially **T1** with two CF_3_ groups, since it is well-known that that presence of fluorine atoms in the structure would improve metabolic stability, bioavailability, and protein–ligand interactions, as already demonstrated in fluorinated drugs [66,67].

**Scheme 2:**
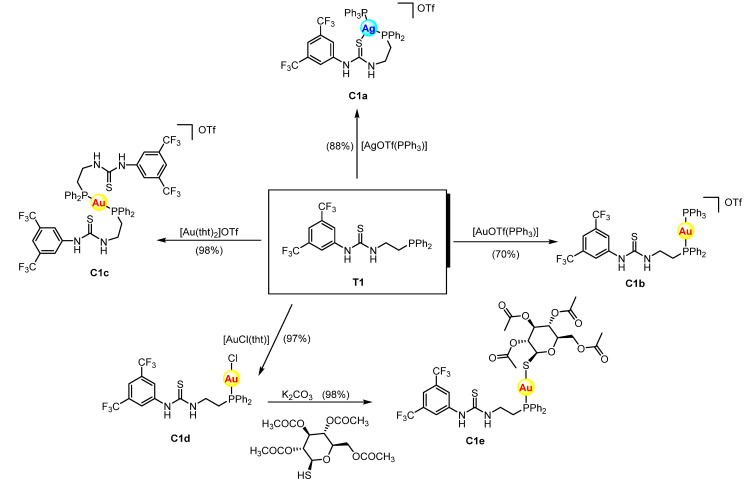
Synthesis of metal complexes **C1a–e** derived from **T1**.

After that, the corresponding metal complexes derived from **T1** were prepared. First, the synthesis of Ag(I) and Au(I) complexes were carried out (Scheme 2). Therefore, complexes **C1a** and **C1b** were synthesized in a 1:1 ratio of **T1** ligand by reaction with complex [M(OTf)(PPh_3_)] (M = Ag, Au), in which the high tendency of silver to higher coordination geometries gives coordination to both the sulfur and phosphorus atoms and for gold only coordination to the phosphorus atom is proposed. The reaction of thiourea **T1** with [AuCl(tht)] affords complex **C1d**, for which substitution of the Cl ligand for a 2,3,4,6-tetra-*O*-acetyl-1-thio-β-d-glucopyranose moiety provides the thiolate gold complex with the sugar fragment, which may enhance the cellular uptake and the selectivity to cancer cells, as they are overexpressed in cancer cells. 

The structures of the synthesized complexes are disclosed in Scheme 2.

All synthesized compounds were characterized by different spectroscopic techniques. A relevant signal in this type of compounds is the phosphorus resonance. Interestingly, in the ^31^P{^1^H} NMR spectrum of **T1**, a singlet was observed at −21.83 ppm that corresponds to the P atom, with the negative value of the chemical shift showing the presence of a non-coordinated phosphorus atom. Therefore, when the P atom is coordinated to the metal atom, its value is shifted towards lower fields. The values of the ^3^^1^P{^1^H} NMR spectra for the ligand and complexes are represented in Table 1.

It is worth noting the complexity of these metal species in terms of the signals disclosed in the spectra of ^31^P{^1^H} NMR. Usually, in silver phosphine complexes, a dissociation of the phosphine ligand takes place and, consequently, these species are in equilibrium. This, together with the presence of two silver isotopes active in NMR, ^109^Ag and ^107^Ag, causes broadening of the signals. For complex **C1a** there are two inequivalent phosphorus atoms, and two broad signals, one of them as a doublet assigned to the average coupling to the silver isotopes. In order to avoid this effect, the NMR was carried out at low temperature and it is expected that the equivalent phosphorus atoms would give rise to two doublets by coupling with the two silver isotopes. Unfortunately, although the NMR was carried out in CD_2_Cl_2_ at −80 °C, no resolution of the signals was observed. In this case, we propose a flat trigonal coordination of silver, with coordination to phosphorus and sulfur atoms of thiourea. 

**Scheme 3:**
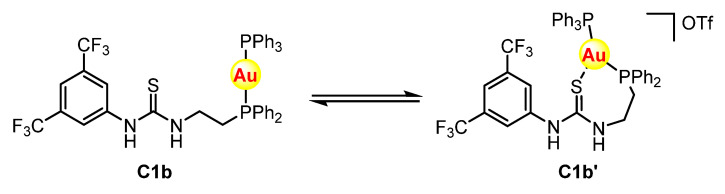
Equilibrium of complex **C1b** in solution.

In the case of complex **C1b**, the gold center has a higher tendency to be linearly coordinated but the presence of the sulfur atom nearby could afford the three-coordination. The ^1^H and the ^31^P{^1^H} NMR spectra showed resonances compatible with the coordination of the gold center to the phosphorus atom, with two slightly broad resonances in the phosphorus spectrum. However, the low temperature spectrum presents equilibrium between the linear **C1b** and the three-coordinated species **C1b’** (Scheme 3), both as an AB system for the inequivalent phosphorus.

Complex **C1c** with the gold center coordinated to two thiourea-phoshine ligands showed a sharp singlet in the ^31^P{^1^H} NMR spectrum even at low temperature, for which linear coordination to the phosphorus atoms is proposed. However, we cannot rule out the possibility of a *pseudo*-tetracoordination around the metal center. Unfortunately, all the attempts to grow crystals in order to confirm the structure by X-ray diffraction were unsuccessful. 

Complex **C1d** is formed by addition of [AuCl(tht)] to the thiourea and also showed a singlet resonance in the ^31^P{^1^H} NMR at lower field corroborating the coordination of the gold center to the phosphorus atom. Substitution of the chloride ligand for a thiolate can be achieved by treatment of **C1d** with 2,3,4,6-tetra-*O*-acetyl-1-thio-β-d-glucopyranose in the presence of a mild base, K_2_CO_3_.

### 2.2. Synthesis of Complexes ***C2a–c***

**Scheme 4:**
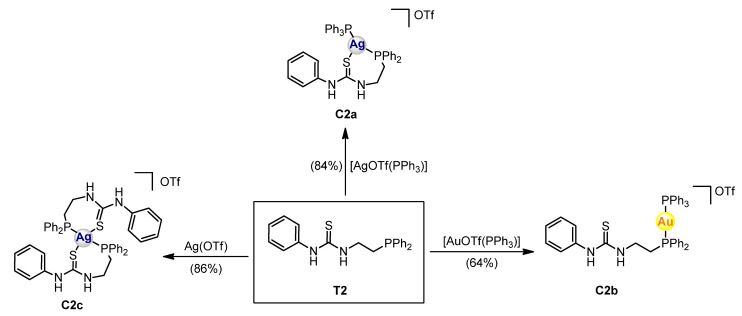
Synthesis of metal complexes **C2a–c** derived from **T2**.

The synthesis of new complexes **C2a–c** was carried out in the same way as for the case of **T1**, in order to compare the cytotoxic effect of both thioureas (Scheme 4).

All complexes were characterized by NMR techniques. As mentioned in the previous example, the ^31^P{^1^H} NMR spectra provide relevant information in the synthesis of these complexes. In this way, the presence of a signal at −21.33 ppm that corresponds to the P of thiourea **T2** is observed, and for the synthesized complexes, signals are shifted towards down field (see the characterization part). Similar spectra to those of thiourea **T1** were found for all metal complexes (see Supporting Information). 

**Figure 3:**
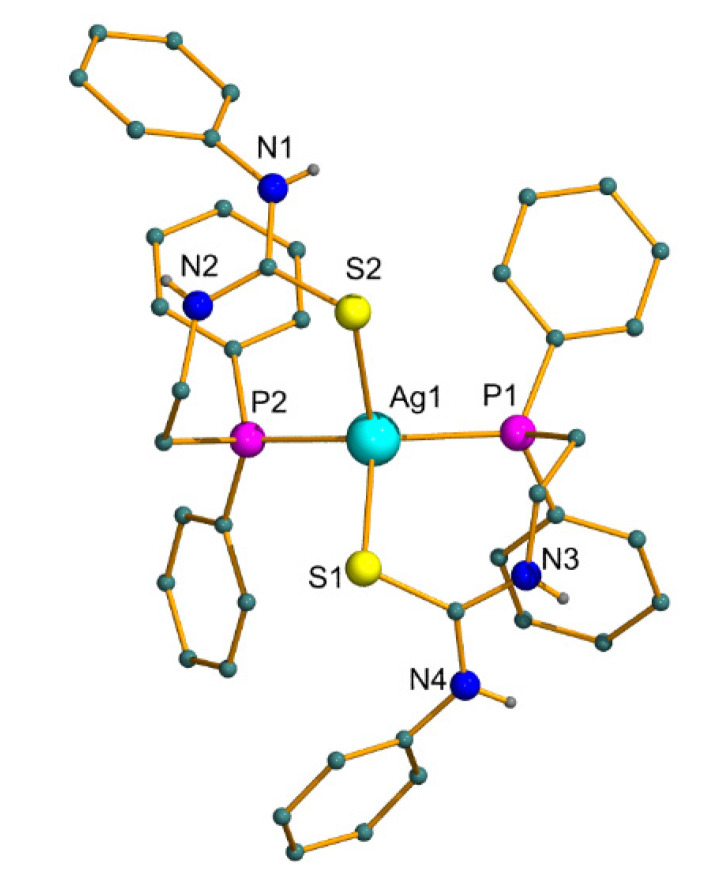
Crystal structure of the cation of complex **C2c**. Hydrogen atoms with the exception of thiourea NH have been omitted for clarity. Selected bond lengths (Å) and angles (°): Ag1-P1 2.4198(11), Ag1-P2 2.4270(13), Ag1-S1 2.6725(8), Ag1-S2 2.7028(9), S1-C41 1.694(2), S2-C42 1.714(2); P1-Ag1-P2 137.81(3), P1-Ag1-S1 101.74(3), P2-Ag1-S1 105.08(3), P1-Ag1-S2 101.87(4), P2-Ag1-S2 102.96(3), S1-Ag1-S2 103.18(3).

Moreover, and interestingly, we were able to obtain the crystal structure of the silver complex **C2c** with two thiourea ligands and the proposed structure was confirmed by X-ray diffraction (Figure 3).

The crystal structure corroborates that the silver atom is coordinated by two thiourea **T2** ligands in a chelated form, through the sulfur and phosphorus atoms. The silver center has a somewhat distorted tetrahedral geometry, mainly due to the chelation angles of the ligand, P1-Ag1-S1 101.74(3)° and P2-Ag1-S2 102.96(3)°, which are slightly lower than the ideal of 109°. The Ag-P distances are Ag1-P1 2.4198(11) and Ag1-P2 2.4270(13) Å, which are in agreement for this type of bonds in tetra-coordinated silver complexes [68]. The Ag-S distances of Ag1-S1 2.6725(8) and Ag1-S2 2.7028(9) Å are longer than those found in other complexes, and consequently there is a weaker bond interaction with these atoms. The triflate counteranion is disordered over two positions and the oxygen atoms form hydrogen bonds with the NH groups of the thiourea.

### 2.3. Biological Study

After the synthesis of the ligands **T1** and **T2** and the resulting metal complexes **C1** and **C2** above described, the cytotoxicity study (IC_50_) was carried using three cancer cell lines: HeLa (human cervical carcinoma), A549 (human lung carcinoma) and Jurkat (leukemia) (Table 2 and Table 3).

Before starting the cytotoxicity measurements, we tested the stability of all complexes in DMSO, as part of the medium used in the *in vitro* assays. Thus, we studied the stability of the complexes **C1** and **C2** in DMSO by ^1^H NMR, verifying that they are stable after one week. The cytotoxicity study performed by the MTT assay with the family of compounds derived from thiourea **T1** and the calculated IC_50_ values for the complexes after 24 h of incubation are shown in Table 2. The values for the cisplatin after 24 h, although dissolved in H_2_O, for A549 and Jurkat and in DMSO for HeLa, have been included for comparison in Table 1 and this supports that the silver and gold complexes with both thioureas are considerably more active.

From these results, it can be concluded that the ligand **T1** showed only a moderate cytotoxic activity in the A549 cells, whereas higher values were found for the other cell lines. Comparing all the cell lines, the complexes were more sensitive to Jurkat cells, for which all the complexes showed very good activity, except complex **C1d**. Interestingly, a certain selectivity of some of the compounds towards HeLa and Jurkat was observed, and this is because the A549 cancer cell line is more resistant. Comparing the gold and silver complexes, most of them showed good activity but the results depend upon the ancillary ligand. Coordination of two thiourea ligands promoted higher cytotoxicity in the gold derivative against HeLa and Jurkat cell lines, while higher cytotoxicity for the same complex is found against A549 cells. For gold complexes, the substitution of the chloride ligand for a thiosugar drastically enhanced the anticancer activity for the three cell lines. 

These values support our initial hypothesis of work, since this demonstrates that the cytotoxic activity of thiourea **T1** is modulated and improved with the presence of a metal, achieving better results with Au(I) complexes. 

After this first promising study, the effects of thiourea **T2** and its derivatives were also analyzed (Table 3).

The results showed a better effect for the thiourea **T2** on the cytotoxicity in the cell lines studied in contrast with the moderate cytotoxicity showed by **T1** in general. Interestingly, all synthesized metal complexes **C2a-c** improved these results.

In some cases, excellent and better results have been obtained with this family of metal complexes in comparison with those derived from **T1** in Table 2. Analyzing independently each cancer line, we have achieved the best results of cytotoxicity in HeLa, reaching values of <1 µM using Ag(I) complex **C2a** (entry 2). Furthermore, the higher value was only around 5 µM with Au(I)-triphenylphosphine complex **C2b** (entry 3) against Jurkat cell line. Regarding the A549 cell line, excellent results were also achieved with Ag(I) complexes **C2a** and **C2c**, 0.79 µM (entry 2) and 0.58 µM (entry 4), respectively.

Finally, with the Jurkat cells, although the complexes are less active than in the other two cell lines, values of 0.64 µM were achieved for the silver complex **C2a** (entry 2). Nevertheless, in all cases very encouraging results were found. 

In general, the best results were observed for **T2** and its derivatives. This may be due to the fact that the aromatic ring can favor the stabilization of thiourea, allowing its complexes to cross the membrane more easily, achieving lower values of cytotoxicity. 

These preliminary results are of high importance because a new field of investigation with thiourea ligands can be opened in which their activity is improved with the formation of Au and Ag complexes. Although these results are still at a very early stage in a long process, it is encouraging to continue in the development of biologically active molecules to combat diseases such as cancer and open the door to further investigations ongoing in our lab. In particular, interaction with key enzymes such as thioredoxin reductase, type of cell death, cellular uptake by ICP-mass, toxicity, etc. 

## 3. Materials and Methods

Purification of reaction products was carried out by column chromatography using silical-gel (0.063–0.200 mm). Analytical thin layer chromatography was performed on 0.25 mm silical gel 60-F plates. ^1^H NMR spectra were recorded at room temperature on a BRUKER AVANCE 400 (Bruker, Billerica, MA, USA) spectrometer (^1^H, 400 MHz) or on a BRUKER AVANCE II 300 (Bruker, Billerica, MA, USA) spectrometer (^1^H, 300 MHz), with chemical shifts (ppm) reported relative to the solvent peaks of the deuterated solvent. CD_2_Cl_2_ and DMSO-*d*_6_ were used as the deuterated solvents. Chemical shifts were reported in the *δ* scale relative to residual CH_2_Cl_2_ (5.32 ppm) and DMSO (2.50 ppm) for ^1^H-NMR and to the central line of CD_2_Cl_2_ (54 ppm) and DMSO-*d*_6_ (39.43 ppm) for ^13^C-APT NMR. Mass spectra were recorded on a BRUKER ESQUIRE 3000 PLUS (Bruker, Boston, MA, USA), with the electrospray (ESI) technique.

All reactions were performed under air atmosphere and solvents and reagents were used as received without further purification or drying. All reagents were commercially available.

### 3.1. Synthesis of Thiourea ***T1***

To a solution of CH_2_Cl_2_ (5 mL), 2-(diphenylphosphino)ethylamine (1.8 mmol, 413.2 mg) and 3,5-bis(trifluoromethyl)phenyl isothiocyanate (2 mmol, 310 µL) were added. The reaction was carried out at room temperature during 18 h. After that, the solvent was partially evaporated, and the product was purified by precipitating with hexane, filtered and successive washes with more hexane (3 × 5 mL). The final thiourea **T1** was obtained as a white solid in 80% yield [70]. ^1^H NMR (300 MHz, CD_2_Cl_2_) δ (ppm): 7.89 (br s, 1H, NH_b_), 7.81 (s, 2H, H-C_5_), 7.72 (s, 1H, H-C_7_), 7.51–7.29 (m, 10H, H-C_10–12_), 6.46 (br s, 1H, NH_a_), 3.79 (td, *J =* 12.5, 6.8 Hz, 2H, H-C_2_), 2.47 (t, *J =* 7.0 Hz, 2H, H-C_1_). ^13^C-APT NMR (101 MHz, CD_2_Cl_2_) δ (ppm): 181.1 (s, 1C, C_3_), 139.7 (s, 1C, C_4_), 137.9 (d, *J =* 11.4 Hz, 2C, C_9_), 133.2 (d, *J =* 19.0 Hz, 4C, C_10_), 132.9 (q, *J =* 32.0 Hz, 2C, C_6_), 129.6 (s, 2C, C_12_), 129.2 (d, *J =* 7.0 Hz, 4C, C_11_), 124.6 (m, 2C, C_5_), 123.5 (q, *J =* 273.7 Hz, 2C, C_8_), 119.8 (m, 1C, C_7_), 42.9 (d, *J =* 18.8 Hz, 1C, C_2_), 28.1 (d, *J =* 13.0 Hz, 1C, C_1_). ^31^P{^1^H} NMR (162 MHz, CD_2_Cl_2_) δ (ppm): −21.83 (s, 1P, P-Ph_2_). ^19^F NMR (377 MHz, CD_2_Cl_2_) δ (ppm): −63.25 (F-CF_3_). HRMS (ESI+) calculated for C_23_H_19_F_6_N_2_NaPS 523.0803, found 523.0803 [M + Na].

### 3.2. Synthesis of Metal Complexes ***C1a–g***

#### 3.2.1. Synthesis of Complex [Ag(PPh_3_)(**T1**)]OTf (**C1a**)

To a solution of **T1** (0.052 mmol, 26.0 mg) in CH_2_Cl_2_ (8 mL), [AgOTfPPh_3_] (0.052 mmol, 26.9 mg) was added. The reaction was carried out at room temperature during 30 min covered from the light. After the indicated reaction time, the solvent was partially evaporated, precipitated with hexane, and filtered to give a white solid in 88% yield. ^1^H NMR (400 MHz, CD_2_Cl_2_) δ (ppm): 9.37 (br s, 1H, NH_b_), 8.54 (s, 1H, NH_a_), 7.77 (s, 2H, H-C_5_), 7.65 (s, 1H, H-C_7_), 7.59–7.06 (m, 25H, H-C_10–12_, H-C_14–16_), 4.51–4.19 (m, 2H, H-C_2_), 2.88–2.60 (m, 2H, H-C_1_). ^13^C-APT NMR (101 MHz, CD_2_Cl_2_) δ (ppm): 181.1 (s, 1C, C_3_), 140.5 (s, 1C, C_4_), 134.2 (d, *J =* 16.0 Hz, 6C, C_14_), 133.1 (d, *J =* 16.0 Hz, 4C, C_10_), 131.9 (q, *J =* 33.0 Hz, 2C, C_6_), 131.7 (s, 3C, C_13_), 131.5 (s, 3C, C_16_), 131.4 (d, *J =* 7.0 Hz, 2C, C_12_), 129.9 (m, 4C, C_11_), 129.7 (d, *J =* 10 Hz, 6C, C_15_), 125.3 (m, 2C, C_5_), 123.7 (q, *J =* 273.7 Hz, 2C, C_8_), 120.9 (q, *J =* 315.2 Hz, 1C, OTf), 119.4 (m, 1C, C_7_), 43.2 (m, 1C, C_2_), 29.2 (s, 1C, C_1_). ^31^P{^1^H} NMR (162, CD_2_Cl_2_) δ (ppm): 12.55 (Ph_3_P-Ag), 5.89–3.41 (Ph_2_P-Ag). ^19^F NMR (377 MHz, CD_2_Cl_2_) δ (ppm): −63.16 (F-CF_3_), −79.00 (F-OTf). HRMS (ESI+) calculated for C_41_H_34_AgF_6_N_2_P_2_S 869.0868, found 869.0868 [M − OTf].

#### 3.2.2. Synthesis of Complex [Au(PPh_3_)(**T1**)]OTf (**C1b**)

To a solution of CH_2_Cl_2_ (5 mL), [AuCl(PPh_3_)] (0.1 mmol, 49.5 mg) and Ag(OTf) (0.12 mmol, 30.8 mg) were added. The reaction was carried out at room temperature during 1 h covered from the light. The generated AgCl was filtered by diatomaceous earth and washed with CH_2_Cl_2_. The white solid [Au(PPh_3_)](OTf) was obtained after evaporation of the solvent under vacuum. To the resulting solution in CH_2_Cl_2_ (5 mL), **T1** (0.1 mmol, 50.0 mg) was added. The solution was reacting during 1 h and, after that, the solvent was partially evaporated, precipitated with hexane, and filtered to give a white solid in 70% yield. ^1^H NMR (400 MHz, CD_2_Cl_2_) δ (ppm): 9.13 (br s, 1H, NH_b_), 8.30 (br s, 1H, NH_a_), 7.83 (s, 2H, H-C_5_), 7.78–7.63 (m, 5H, H-C_7_, 4H-Ar), 7.61–7.28 (m, 21H, H-Ar), 4.33–4.14 (m, 2H, H-C_2_), 3.10–2.95 (m, 2H, H-C_1_). ^13^C-APT NMR (101 MHz, CD_2_Cl_2_) δ (ppm): 181.3 (s, 1C, C_3_), 141.2 (s, 1C, C_4_), 134.4 (d, *J =* 14.4 Hz, 6C, C_14_), 133.5 (d, *J =* 12.8 Hz, 4C, C_10_), 132.2 (d, *J =* 20.9 Hz, 2C, C_12_), 132.2 (s, 3C, C_16_), 131.9 (q, *J =* 33.3 Hz, 2C, C_6_), 131.3 (m, 3C, C_13_), 130.6 (m, 2C, C_9_), 129.9 (d, *J =* 13.0 Hz, 4C, C_11_), 129.8 (d, *J =* 10.9 Hz, 6C, C_15_), 123.9 (q, *J =* 273.7 Hz, 2C, C_8_), 123.8 (m, 2C, C_5_), 121.2 (q, *J =* 321.2 Hz, 1C, OTf), 118.0 (m, 1C, C_7_), 41.2 (s, 1C, C_2_), 29.6 (d, *J =* 29.1 Hz, 1C, C_1_). ^31^P{^1^H} NMR (162 MHz, CD_2_Cl_2_) δ (ppm): 36.60 (P-PPh_3_), 34.52 (P-PPh_2_). ^19^F-RMN (373 MHz, CD_2_Cl_2_) δ (ppm): −63.13 (F-CF_3_), −78.96 (F-OTf). HRMS (ESI+) calculated for C_41_H_34_AuF_6_N_2_P_2_S 959.1482, found 959.1484 [M − OTf].

#### 3.2.3. Synthesis of Complex [Au(**T1**)_2_]OTf (**C1c**)

To a solution of **T1** (0.1 mmol, 50.2 mg) in CH_2_Cl_2_ (8 mL), [Au(tht)_2_](OTf) (0.05 mmol, 26.1 mg) was added after generation in situ. For the preparation of this species, [Ag(tht)OTf] (0.055 mmol, 19 mg) reacts with [AuCl(tht)] (0.055 mmol, 17.6 mg) in a solution with CH_2_Cl_2_ (8 mL) for 2 h covered from the light. The generated AgCl was filtered by diatomaceous earth and washed with CH_2_Cl_2_. The white solid [Au(tht)_2_](OTf) was obtained after evaporation of the solvent under vacuum. Then, **T1** was added to this solution and it was allowed to react during 45 min at room temperature. After the indicated reaction time, the solvent was partially evaporated, precipitated with hexane, and filtered to give a white solid in 98% yield. ^1^H NMR (400 MHz, CD_2_Cl_2_) δ (ppm): 8.72 (br s, 1H, NH_b_), 7.85 (s, 2H, H-C_5_), 7.79–7.66 (m, 5H, H-Ar), 7.56 (s, 1H, H-C_7_), 7.52–7.40 (m, 6H, NH_a_, H-Ar), 4.30–4.07 (m, 2H, H-C_2_), 3.15–3.01 (m, 2H, H-C_1_). ^13^C-APT NMR (75 MHz, CD_2_Cl_2_) δ (ppm): 181.5 (s, 2C, C_3_), 140.8 (s, 2C, C_4_), 133.7 (m, 8C, C_10_), 132.6 (m, 4C, C_12_), 131.9 (q, *J =* 33.0 Hz, 4C, C_6_), 130.1 (m, 8C, C_11_), 129.6 (m, 4C, C_9_), 123.8 (q, *J =* 271.5 Hz, 4C, C_8_), 123.6 (s, 4C, C_5_), 120.6 (q, 1C, OTf), 118.4 (m, 2C, C_7_), 41.3 (m, 2C, C_2_), 28.3 (m, 2C, C_1_). ^31^P{^1^H} NMR (121 MHz, CD_2_Cl_2_) δ (ppm): 36.22 (P-Au). ^19^F NMR (282 MHz, CD_2_Cl_2_) δ (ppm): −63.32 (F-CF_3_), −78.94 (F-OTf). HRMS (ESI+) calculated for C_46_H_38_AuF_12_N_4_P_2_S_2_ 1197.1482, found 1197.1450 [M − OTf].

#### 3.2.4. Synthesis of Complex [Au(Cl)(**T1**)] (**C1d**)

To a solution of **T1** (0.12 mmol, 71.4 mg) in CH_2_Cl_2_ (10 mL), [AuCl(tht)] (0.119 mmol, 38.1 mg) was added. The reaction was carried out at room temperature during 30 min. After the indicated reaction time, the solvent was partially evaporated, precipitated with hexane, and filtered to give a white solid in 97% yield. ^1^H NMR (400 MHz, CD_2_Cl_2_) δ (ppm): 10.57 (br s, 1H, NH_b_), 8.46 (br s, 1H, NH_a_), 7.91 (s, 2H, H-C_5_), 7.78–7.61 (m, 5H, H-C_7_, H-C_10–12_), 7.58–7.38 (m, 6H, H-C_10–12_), 4.13–3.93 (m, 2H, H-C_2_), 3.19–2.96 (m, 2H, H-C_1_). ^13^C-APT NMR (101 MHz, CD_2_Cl_2_) δ (ppm): 189.5 (s, 1C, C_3_), 139.7 (s, 1C, C_4_), 133.9 (d, *J =* 13.4 Hz, 4C, C_10_), 132.7 (d, *J =* 2.1 Hz, 2C, C_12_), 132.4 (q, *J =* 33.0 Hz, 2C, C_6_), 130.0 (d, *J =* 11.7 Hz, 4C, C_11_), 128.9 (d, *J =* 60.3 Hz, 2C, C_9_), 126.2 (s, 2C, C_5_), 124.6 (s, 1C, C_7_), 123.5 (q, *J =* 273.7 Hz, 2C, C_8_), 41.8 (d, *J =* 6.6 Hz, 1C, C_2_), 30.3 (d, 1C, C_1_). ^31^P{^1^H} NMR (162 MHz, CD_2_Cl_2_) δ (ppm): 24.56 (s, P-Ph_2_). ^19^F NMR (373 MHz, CD_2_Cl_2_) δ (ppm): −63.15 (F-CF_3_). HRMS (ESI+) calculated for C_23_H_19_AuF_6_N_2_PS 697.0571, found 697.0571 [M − Cl].

#### 3.2.5. Synthesis of Complex [Au(2,3,4,6-tetra-*O*-acetyl-1-thio-β-d-glucopyranose)(**T1**)] (**C1e**)

To a solution of complex **C1d** (0.075 mmol, 56.1 mg) in CH_2_Cl_2_ (10 mL), 2,3,4,6-tetra-*O*-acetyl-1-thio-β-d-glucopyranose (0.075 mmol, 27.3 mg) and an excess of K_2_CO_3_ (1.5 equivalents) were added. The reaction was carried out at room temperature during 5 h. After the indicated reaction time, the base was filtered, and the solvent was partially evaporated, precipitated with hexane, and filtered again, to give a white solid in 98% yield. ^1^H NMR (400 MHz, CD_2_Cl_2_) δ (ppm): 8.79 (s, 1H, NH_b_), 8.17–7.15 (m, 14H, NH_a_, H-Ph), 5.27–4.88 (m, 4H, H-C_13–16_), 4.36–4.051 (m, 3H, H-C_2_, H-C_17_), 4.00–3.61 (m, 2H, H-C_1_), 3.43–3.19 (m, 1H, H-C_18_), 3.06–2.80 (m, 1H, H-C_18_), 2.26–1.73 (m, 12H, H-C_20_, H-C_22_, H-C_24_, H-C_26_). ^13^C-APT NMR (101 MHz, CD_2_Cl_2_) δ (ppm): 181.5 (s, 1C, C_3_), 171.0, 170.7, 170.2, 169.9 (s, 4C, C_19_, C_21_, C_23_, C_25_), 133.9 (dd, *J =* 3.0 Hz, 13.0 Hz, 4C, C_10_), 132.4 (s, 2C, C_5_), 131.8 (q, *J =* 33.3 Hz, 2C, C_6_), 130.7 (d, *J =* 7.0 Hz, 2C, C_9_), 129.9 (dd, *J =* 3.3 Hz, 11.3 Hz, 4C, C_11_), 123.95 (q, *J =* 274.7 Hz, 2C, C_8_), 123.7 (m, 4C, C_12_), 118.1 (m, 1C, C_7_), 84.3 (s, 1C, C_13_), 79.3 (s, 1C, C_14_), 76.6 (s, 1C, C_15_), 74.9 (s, 1C, C_17_), 69.0 (s, 1C, C_17_), 62.9 (s, 1C, C_18_), 41.0 (s, 1C, C_2_), 28.0 (d, 1C, C_1_), 21.0 (s, 4C, C_20_, C_22_, C_24_, C_26_). ^31^P{^1^H} RMN (162 MHz, CD_2_Cl_2_) δ (ppm): 30.80 (s, 1P, Au-P). ^19^F NMR (373 MHz, CD_2_Cl_2_) δ (ppm): −63.23 (F-CF_3_). HRMS (ESI+) calculated for C_37_H_38_AuF_6_N_2_O_9_PS_2_Na 1083.1218, found 1083.1196 [M + Na].

### 3.3. Synthesis of Thiourea ***T2***

To a solution of CH_2_Cl_2_ (5 mL) and 2-(diphenylphosphino)ethylamine (1.8 mmol, 418.2 mg), phenyl isothiocyanate (2.0 mmol, 244 µL) was added. The reaction was carried out at room temperature during 18 h. After that, the solvent was evaporated and the product was purified by column chromatography (SiO_2_, hexane:AcOEt, 7:3), obtaining the final product **T2** as a white solid in 85% yield [71]. ^1^H NMR (400 MHz, CD_2_Cl_2_) δ (ppm): 7.66 (br s, 1H, NH_b_), 7.51–7.08 (m, 15H, H-C_5–7_, H-C_9–11_), 6.29 (br s, 1H, NH_a_), 3.82–3.66 (m, 2H, H-C_2_), 2.47–2.37 (m, 2H, H-C_1_). ^13^C-APT NMR (101 MHz, CD_2_Cl_2_) δ (ppm): 181.1 (s, 1C, C_3_), 138.3 (d, *J* = 12.2 Hz, 2C, C_8_), 136.8 (s, 1C, C_4_), 133.2 (d, *J =* 19.1 Hz, 4C, C_9_), 130.6 (s, 2C, C_11_), 129.4 (s, 2C, C_6_), 129.1 (d, *J =* 6.9 Hz, 4C, C_10_), 127.5 (s, 1C, C_7_), 125.6 (s, 2C, C_5_), 43.1 (d, *J* = 21.1 Hz, 1C, C_2_), 28.4 (d, *J* = 13.4 Hz, 1C, C_1_). ^31^P{^1^H} NMR (162 MHz, CD_2_Cl_2_) δ (ppm): −21.33 (P-PPh_2_). HRMS (ESI+) calculated for C_21_H_21_N_2_NaPS 387.1055, found 387.1055 [M + Na].

### 3.4. Synthesis of Metal Complexes ***C2a–e***

#### 3.4.1. Synthesis of Complex [Ag(PPh_3_)(**T2**)]OTf (**C2a**)

To a solution of **T2** (0.05 mmol, 18.2 mg) in CH_2_Cl_2_ (8 mL), [Ag(OTf)(PPh_3_)] (0.05 mmol, 25.9 mg) was added. The reaction was stirring at room temperature during 1 h. After the indicated reaction time, the solvent was partially evaporated, precipitated with hexane, and filtered to give a white solid in 84% yield. ^1^H NMR (400 MHz, CD_2_Cl_2_): 8.69 (s, 1H, NH_b_), 7.65–7.11 (m, 30H, H-Ar), 7.01 (br s, 1H, NH_a_), 4.48–4.13 (m, 2H, H-C_2_), 2.83–2.56 (m, 2H, H-C_1_). ^1^H NMR (300 MHz, CD_2_Cl_2_) δ (ppm): 8.69 (br s, 1H, NH_b_), 7.65–7.01 (m, 30H, H-Ar), 7.01 (br s, 1H, NH_a_), 4.48–4.13 (m, 2H, H-C_2_), 2.83–2.56 (m, 2H, H-C_1_). ^13^C-APT NMR (101 MHz, CD_2_Cl_2_) δ (ppm): 180.4 (s, 1C, C_3_), 134.2 (d, *J =* 16.1 Hz, 6C, C_13_), 133.2 (d, *J =* 16.2 Hz, 4C, C_9_), 132.1 (d, *J =* 28.0 Hz, 3C, C_12_), 131.2 (d, *J =* 8.9 Hz, 4C, C_10_), 129.8 (s, 2C, C_6_), 129.7 (s, 6C, C_14_), 129.6 (m, 3C, C_15_), 129.5 (s, 2C, C_11_), 127.0 (m, 1C, C_7_), 125.8 (m, 2C, C_5_), 121.1 (q, *J =* 319.8 Hz, 1C, OTf), 43.3 (m, 1C, C_2_), 29.1 (s, 1C, C_1_). ^31^P{^1^H} NMR (162 MHz, CD_2_Cl_2_) δ (ppm): 12.21 (P-PPh_3_), 1.59 (P-PPh_2_). ^19^F NMR (373 MHz, CD_2_Cl_2_) δ (ppm): −78.95 (F-OTf). HRMS (ESI+) calculated for C_39_H_36_AgN_2_P_2_S 735.1121, found 735.1096 [M − OTf].

#### 3.4.2. Synthesis of Complex [Au(PPh_3_)(**T2**)]OTf (**C2b**)

To a solution of [Au(OTf)(PPh_3_)] (0.05 mmol, 30.4 mg), prepared in situ as above mentioned, in CH_2_Cl_2_ (5 mL), **T2** (0.05 mmol, 18.2 mg) was added. The reaction was carried out at room temperature during 2.5 h. After the indicated reaction time, the solid was partially evaporated, precipitated with hexane, and filtered to give a white solid in 64% yield. ^1^H NMR (400 MHz, CD_2_Cl_2_) δ (ppm): 8.44 (br s, 1H, NH_b_), 7.93–6.91 (m, 31H, NH_a_, H-Ar), 4.38–4.12 (m, 2H, H-C_2_), 3.06–2.81 (m, 2H, H-C_1_). ^13^C-APT NMR (101 MHz, CD_2_Cl_2_) δ (ppm): 181.1 (s, 1C, C_3_), 138.3 (s, 1C, C_4_), 134.5 (d, *J =* 14.6 Hz, 6C, C_13_), 133.4 (d, *J =* 13.5 Hz, 4C, C_9_), 132.0 (d, *J =* 17.1 Hz, 4C, C_10_), 131.6 (d, *J =* 9.09 Hz, 3C, C_12_), 131.1 (d, *J =* 5.1 Hz, 2C, C_8_), 129.9 (s, 2C, C_11_), 129.7 (d, *J =* 10.9 Hz, 6C, C_14_), 129.2 (s, 2C, C_6_), 126.3 (s, 1C, C_7_), 125.2 (s, 2C, C_5_), 121.1 (q, *J =* 320.2 Hz, 1C, OTf), 41.9 (s, 1C, C_2_), 29.3 (d, *J =* 28.1 Hz, 1C, C_1_). ^31^P{^1^H} NMR (162 MHz, CD_2_Cl_2_): 36.92 (s, 1P, PPh_3_) 35.51 (m, 1P, PPh_2_). ^19^F NMR (373 MHz, CD_2_Cl_2_) δ (ppm): −78.91 (F-OTf). HRMS (ESI+) calculated for C_39_H_36_AuN_2_P_2_S 823.1734, found 823.1735 [M − OTf].

#### 3.4.3. Synthesis of Complex [Ag(**T2**)_2_]OTf (**C2c**)

To a solution of **T2** (0.10 mmol, 36.4 mg) in CH_2_Cl_2_ (8 mL), [Ag(OTf)] (0.05 mmol, 12.8 mg) was added. The reaction was carried out at room temperature during 1.5 h. After the indicated reaction time, the solid was partially evaporated, precipitated with hexane, and filtered to give a white solid in 86% yield. ^1^H NMR (400 MHz, DMSO-*d_6_*) δ (ppm): 9.91 (br s, 2H, NH_b_), 7.92–7.06 (m, 28H, NH_a_, H-C_5–7_, H-C_9–11_), 6.79–6.52 (m, 4H, H-Ar), 4.35–4.04 (m, 4H, H-C_2_), 2.82–2.61 (m, 4H, H-C_1_). ^13^C-APT NMR (101 MHz, DMSO-*d_6_*) δ (ppm): 178.2 (s, 2C, C_3_), 136.7 (s, 2C, C_4_), 134.0 (m, 4C, C_8_), 132.5 (s, 8C, C_9_), 130.2 (s, 4C, C_11_), 129.0 (s, 8C, C_10_), 128.9 (s, 4C, C_6_), 125.9 (m, 2C, C_7_), 124.8 (s, 4C, C_5_), 42.8 (s, 2C, C_2_), 27.8 (s, 2C, C_1_). ^31^P{^1^H} NMR (162 MHz, DMSO-*d_6_*) δ (ppm): 2.33, 0.00 (Ph_2_P-Ag). ^19^F NMR (373 MHz, DMSO-*d_6_*) δ (ppm): −77.71 (s, 1F, OTf). HRMS (ESI+) calculated for C_42_H_42_AuN_4_P_2_S_2_ 925.1986, found 925.1916 [M − OTf].

### 3.5. Cytotoxicity Assay 

The MTT assay was used to determine cell viability as an indicator of cell sensitivity to the compounds. Exponentially growing cells were seeded at a density of approximately 104 cells per well (HeLa and A549) or 5 × 10^5^ cells/mL (Jurkat) in 96-well flat-bottomed microplates and allowed to attach for 24 h before the addition of compounds (HeLa and A549). The compounds were dissolved in DMSO and added to cells in concentrations ranging from 0.25 to 100 μM in quadruplicate. Cells were incubated with the tested compounds for 24 h at 37 °C. Then, 10 μL of MTT (5 mg mL^−1^) were added to each well and plates were incubated for 2 h at 37 °C. Finally, media was eliminated (plates were centrifuged for 20 min at 2500 rpm in case of Jurkat cell culture) and DMSO (100 μL per well) was added to dissolve the formazan precipitate. The optical density was measured at 550 nm using a 96-well multi scanner auto reader (ELISA). The IC_50_ was calculated by nonlinear regression analysis using OriginPro software. Each compound was analyzed at least in three independent experiments.

### 3.6. X-ray Diffraction Studies

Crystals were mounted in inert oil on glass fibres and transferred to the cold gas stream of an Xcalibur Oxford Diffraction diffractometer equipped with a low-temperature attachment. Data were collected using monochromated MoKα radiation (λ = 0.71073 Å). Scan type ϖ. Absorption corrections based on multiple scans were applied using spherical harmonics implemented in SCALE3 ABSPACK scaling algorithm [72]. The structures were solved by direct methods and refined on F^2^ using the program SHELXL-2016 [73]. All non-hydrogen atoms were refined anisotropically. CCDC deposition numbers 2,114,494 (**C2c**), contain the supplementary crystallographic data. These data can be obtained free of charge by The Cambridge Crystallography Data Center.

## 4. Conclusions

The synthesis of thiourea ligands bearing a phosphine moiety has been carried out with the purpose to study the coordination modes with Au and Ag metal atoms and check the suitability of the final complexes as anticancer drugs. The differences among both metal centers in terms of coordination geometries has allowed the preparation of linear gold complexes, with the metal only bonded to the phosphorus atom, or trigonal planar and tetrahedral silver complexes with the ligand coordinated as P^S chelate. The thiourea ligands and their complexes were explored by the MTT assay against different cancer cell lines (HeLa, A549 and Jurkat). The results showed that thiourea ligands do not exhibit relevant cytotoxicity in the tested cell lines, only a moderate activity was found for the trifluoromethyl-phenyl substituted thiourea in A549, while somehow activity was obtained with **T2** against HeLa and Jurkat cell lines. Coordination of the metal center triggers excellent cytotoxic values in most of the cases, also in comparison with previous reported works, where thiourea-metal complexes were also explored in cancer cell lines [64,65].

In the complexes with thiourea **T1**, very low IC_50_ values were found in general for gold complexes, although the cytotoxic activity depends upon the ancillary ligand bound to the metal, with bis-thiourea complex affording the most active species. For thiourea **T2** all complexes exhibit better cytotoxicity in general. 

This study demonstrates that metal complexes derived from thiourea are biologically active and that they could become a promising research line as a new class of metal-based drug leads. Although additional studies would be required to identify the mechanisms of action of these metal complexes, these compounds are promising leads for further development as potential anti-cancer agents.

## Figures and Tables

**Table 1:** ^31^P{^1^H} NMR (ppm) data for **T1** and complexes **C1**.

Compound	δ 31P{1H} NMR (ppm)
**T1**	−21.83 (s)
**C1a**	12.55 (m) and 5.89–3.41 (m)
**C1b**	36.60 (m) and 34.52 (m)
**C1c**	36.22 (s)
**C1d**	24.56 (s)
**C1e**	30.80 (s)

**Table 2:** Antitumor activity of complexes **C1a–e** expressed in IC_50_ values compared with that of thiourea **T1** as reference.

Entry	Compound	IC_50_ (µM) Values for Cell Lines ^a^		
		HeLa	A549	Jurkat
1	**T1**	>25	13.89 ± 4.0	>25
2	[**T1**-Ag-PPh_3_]OTf (**C1a**)	10.17 ± 1.74	7.06 ± 1.95	3.89 ± 0.19
3	[**T1**-Au-PPh_3_]OTf (**C1c**)	2.09 ± 0.17	>25	0.62 ± 0.03
4	[**T1**-Au-**T1**]OTf (**C1b**)	0.25 ± 0.12	>25	0.70 ± 0.06
5	[**T1**-Au-Cl] (**C1d**)	>25	>25	19.80 ± 0.46
6	[**T1**-Au-SR] (**C1e**)	4.52 ± 0.23	5.98 ± 1.18	2.57 ± 0.15
7	Cisplatin	55 ± 9 ^b^	114.2 ± 9.1 ^c^	10.8 ± 1.2 ^c^

^a^ Each value represents the mean ± standard deviation from three independent experiments. ^b^ Cisplatin dissolved in DMSO [26]. ^c^ Cisplatin dissolved in H_2_O [69].

**Table 3:** Antitumor activity of complexes **C2a–c** expressed in IC_50_ values compared with that of thiourea **T2** as reference.

Entry	Compound	IC_50_ (µM) Values for Cell Lines ^a^		
		HeLa	A549	Jurkat
1	**T2**	8.16 ± 0.15	>25	14.20 ± 0.72
2	[**T2**-Ag-PPh_3_]OTf (**C2a**)	0.87 ± 0.06	0.79 ± 0.04	0.64 ± 0.04
3	[**T2**-Au-PPh_3_]OTf (**C2b**)	1.48 ± 0.15	4.91 ± 0.23	5.15 ± 0.32
4	[**T2**-Ag-**T2**]OTf (**C2c**)	1.52 ± 0.09	0.58 ± 0.02	1.53 ± 0.31

^a^ Each value represents the mean ± standard deviation from three independent experiments.

## Data Availability

Data is contained within the article or Supplementary Material.

## Supplementary Materials

The following are available online, Figure S1: ^1^H, ^13^C-APT, ^31^P{^1^H} and ^19^F NMR spectra (CD_2_Cl_2_) of **T1**, Figure S2: ^1^H, ^13^C-APT, ^31^P{^1^H} and ^19^F NMR spectra (CD_2_Cl_2_) for complex **C1a**, Figure S3: ^1^H NMR spectrum (DMSO-*d_6_*) for complex **C1a**, Figure S4: Spectra of ^1^H, ^13^C-APT, ^31^P{^1^H} and ^19^F NMR spectra (CD_2_Cl_2_) for complex **C1b**, Figure S5: Spectra of ^1^H, ^13^C-APT, ^31^P{^1^H} and ^19^F NMR spectra (CD_2_Cl_2_) for complex **C1c**, Figure S6: ^1^H, ^13^C-APT, ^31^P{^1^H} and ^19^F NMR **s**pectra (CD_2_Cl_2_) for complex **C1d**, Figure S7: ^1^H, ^13^C-APT, ^31^P{^1^H} and ^19^F NMR spectra (CD_2_Cl_2_) of complex **C1e**, Figure S8: ^1^H, ^13^C-APT and ^31^P{^1^H} NMR spectra (CD_2_Cl_2_) of **T2**, Figure S9: ^1^H, ^13^C-APT, ^31^P{^1^H} and ^19^F NMR spectra (CD_2_Cl_2_) for complex **C2a**, Figure S10: ^1^H NMR spectrum (DMSO-*d_6_*) for complex **C2a**, Figure S11: ^1^H, ^13^C-APT, ^31^P{^1^H} and ^19^F NMR spectra (CD_2_Cl_2_) for complex **C2b**, Figure S12: ^1^H NMR spectrum (DMSO-*d_6_*) for complex **C2b**, Figure S13: ^1^H, ^13^C-APT, ^31^P{^1^H} and ^19^F NMR spectra (DMSO-*d_6_*) for complex **C2c**, Figure S14: ^31^P{^1^H} NMR spectrum (CD_2_Cl_2_) at 193 K for complex **C1a**, Figure S15: ^31^P{^1^H} NMR spectrum (CD_2_Cl_2_) at 193 K for complex **C1b**, Figure S16: ^31^P{^1^H} NMR spectrum (CD_2_Cl_2_) at 193 K for complex **C1c**, Figure S17: HSQC and COSY NMR spectra of **C2c** to explain the correlation among protons and proton-carbon on C1 and C2, Figure S18: HSQC and COSY NMR spectra of **C2b** to explain the correlation among protons and proton-carbon on C1 and C2, Figure S19: ESI-mass for all compounds.
