# Supplementary material for: Synthesis of New Thiourea-Metal Complexes with Promising Anticancer Properties
Source: Molecules. 2021 Nov 16;26(22):6891. doi: 10.3390/molecules26226891 (PMC8619901; doi:10.3390/molecules26226891)

Synthesis of new thiourea-metal complexes with promising anticancer properties

Guillermo Canudo-Barreras[1,2], Lourdes Ortego[1], Anabel Izaga[1], Isabel Marzo[3], Raquel P. Herrera[2,*] and M. Concepción Gimeno[1,*]

[1] Departamento de Química Inorgánica, Instituto de Síntesis Química y Catálisis Homogénea (ISQCH) CSIC-Universidad de Zaragoza, C/ Pedro Cerbuna 12, 50009 Zaragoza, Spain; guillermo@canudo.org (G.C.) a_izaga_@hotmail.com (A.I); lourdesortego@hotmail.com (L.O.).

[2] Laboratorio de Organocatálisis Asimétrica. Departamento de Química Orgánica, Instituto de Síntesis Química y Catálisis Homogénea (ISQCH) CSIC-Universidad de Zaragoza, C/ Pedro Cerbuna 12, 50009 Zaragoza, Spain.

[3] Departamento de Bioquímica y Biología Celular, Universidad de Zaragoza, C/ Pedro Cerbuna 12, 50009 Zaragoza, Spain. imarzo@unizar.es (I.M.)

Correspondence: gimeno@unizar.es, +34 976762291 (M.C.G.); raquelph@unizar.es; Tel.: +34 976761190 (R.P.H.).

Figure S1. ^{1}H, ^{13}C-APT, $^{31}P\{^{1}H\}$ and ^{19}F NMR spectra (CD_2Cl_2) of **T1**.

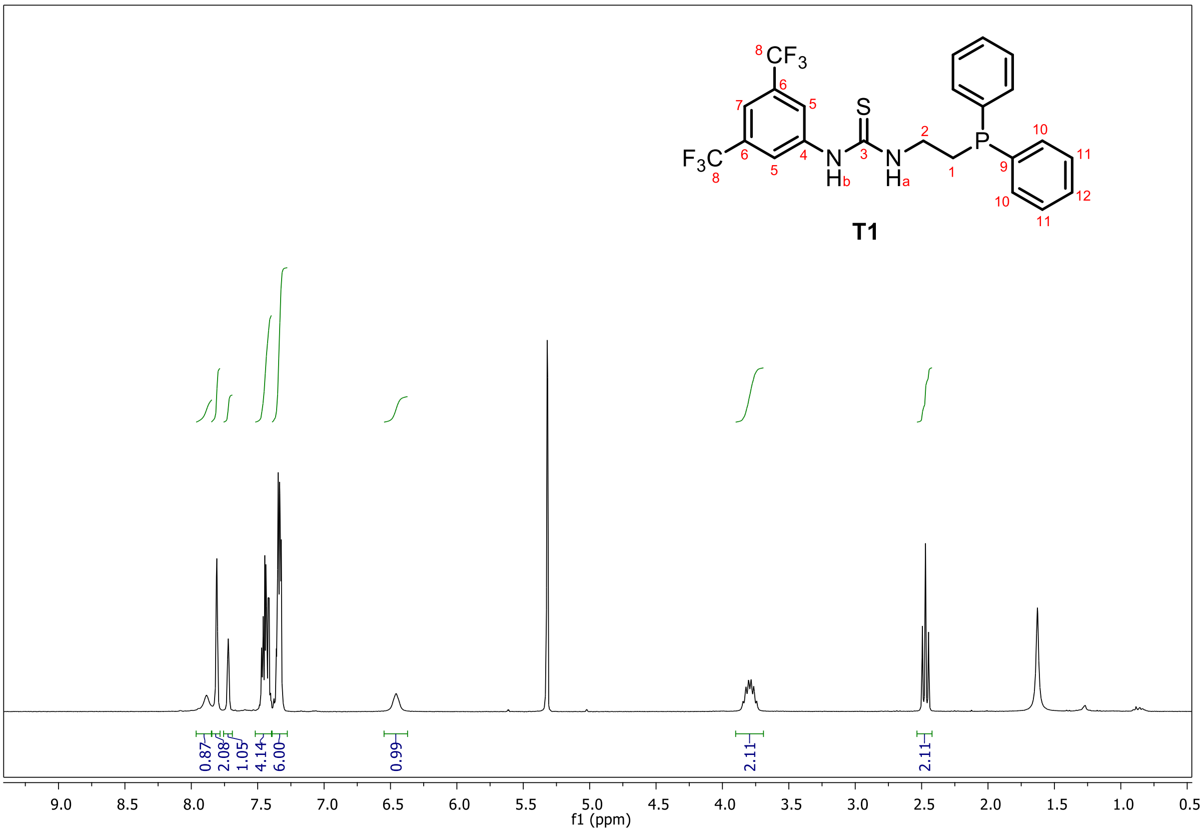

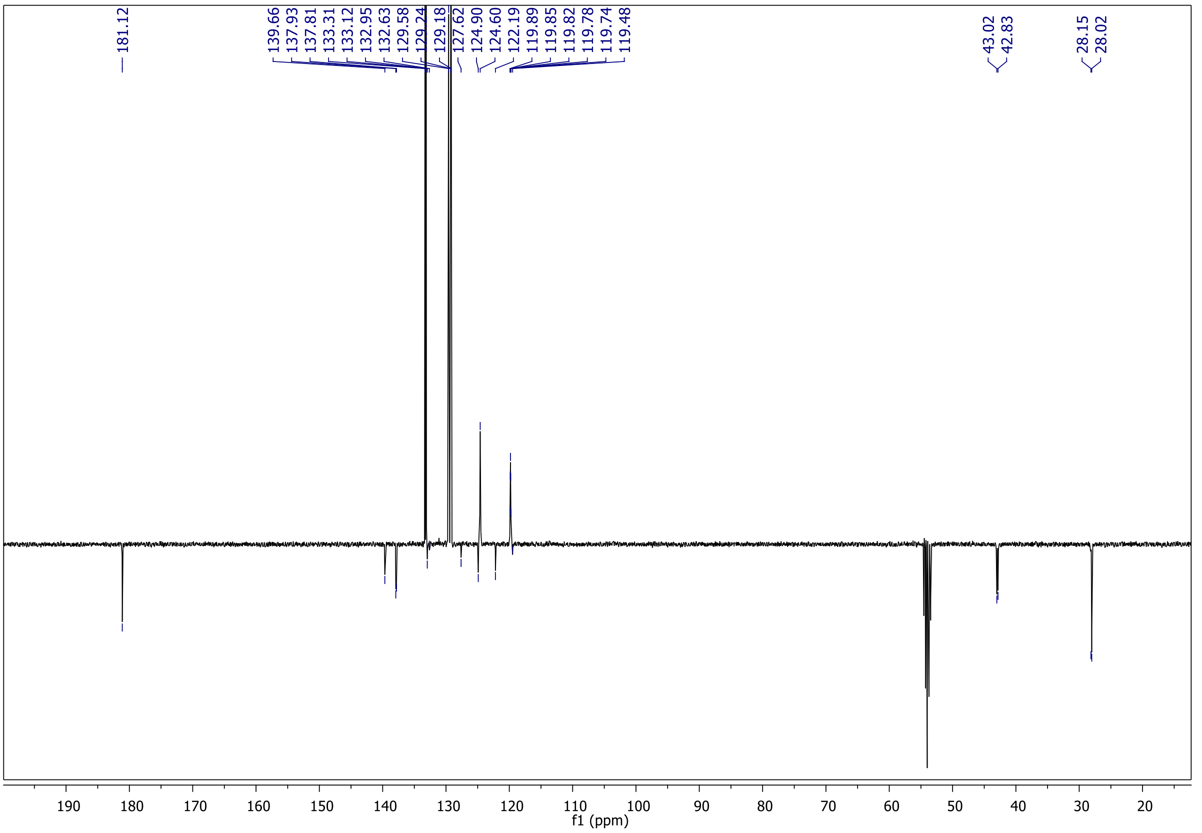

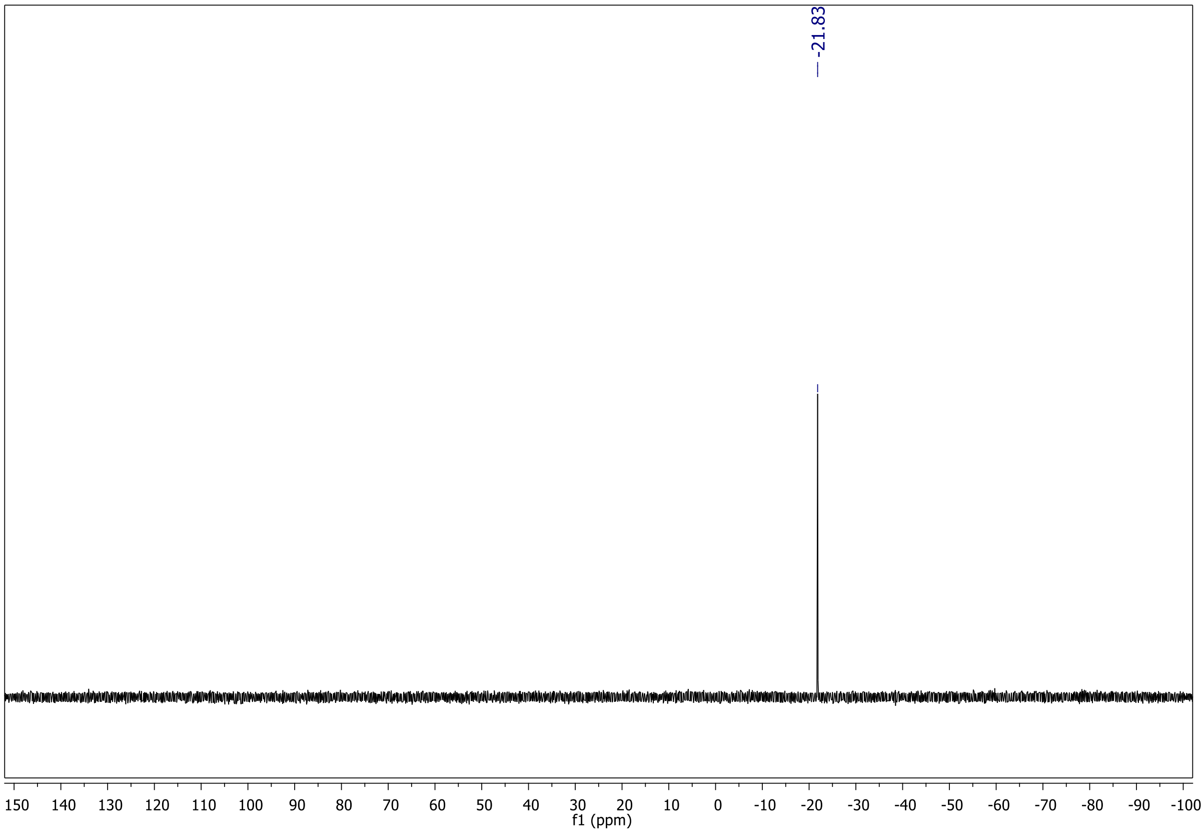

-21.83
150 140 130 120 110 100 90 80 70 60 50 40 30 20 10 0 -10 -20 -30 -40 -50 -60 -70 -80 -90 -100
f1 (ppm)

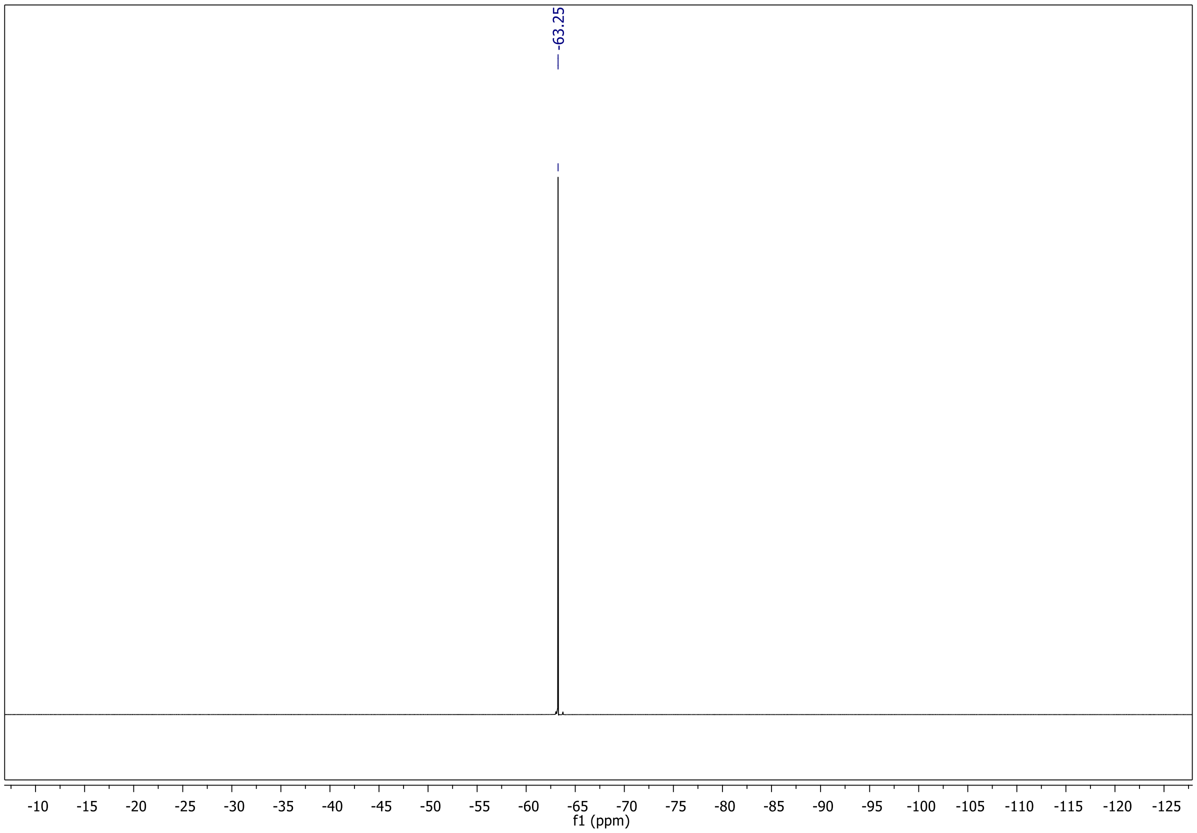

-63.25
-10 -15 -20 -25 -30 -35 -40 -45 -50 -55 -60 -65 -70 -75 -80 -85 -90 -95 -100 -105 -110 -115 -120 -125
f1 (ppm)

Figure S2. ^{1}H, ^{13}C-APT, $^{31}P\{^{1}H\}$ and ^{19}F NMR spectra (CD_2Cl_2) for complex **C1a**.

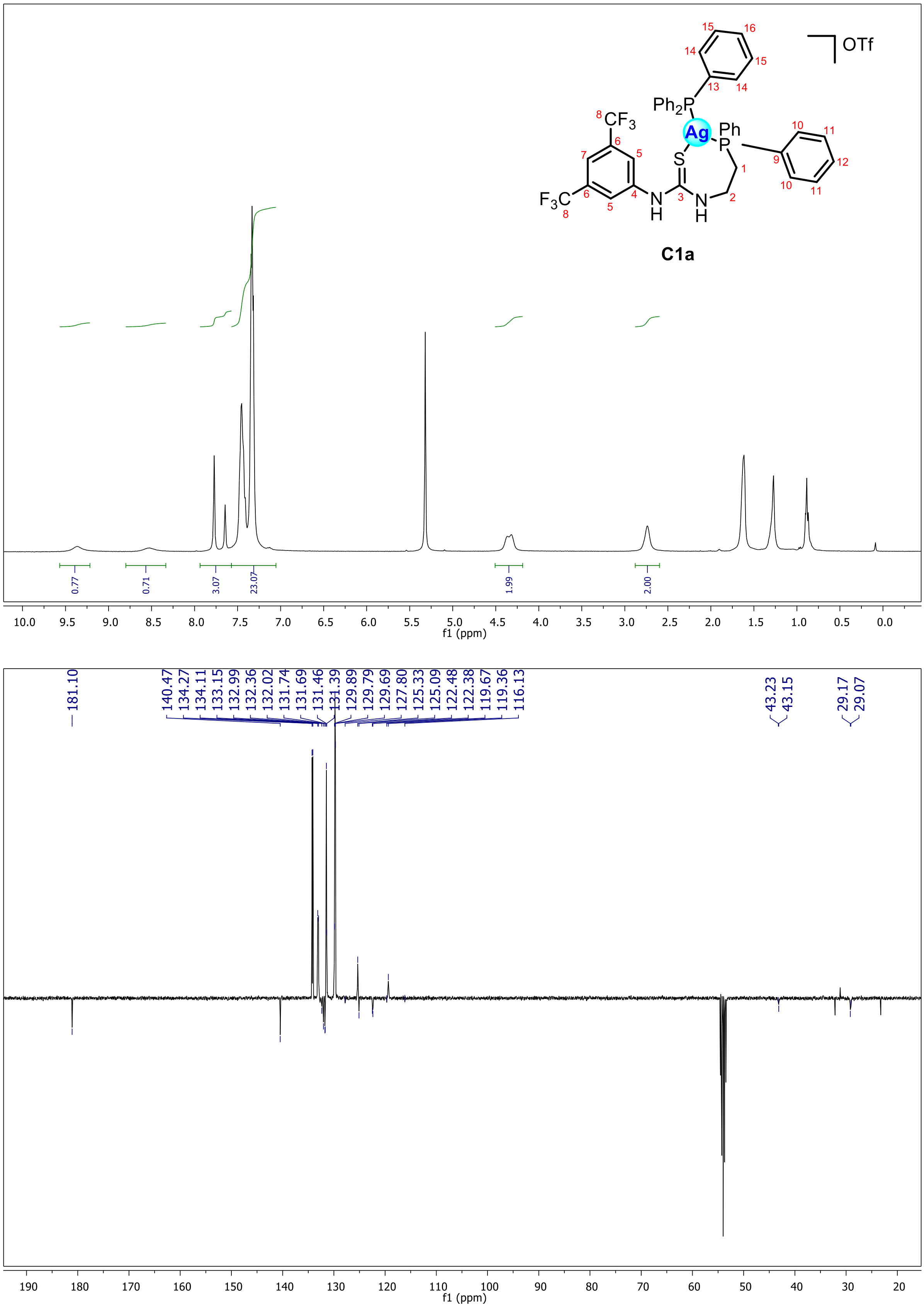

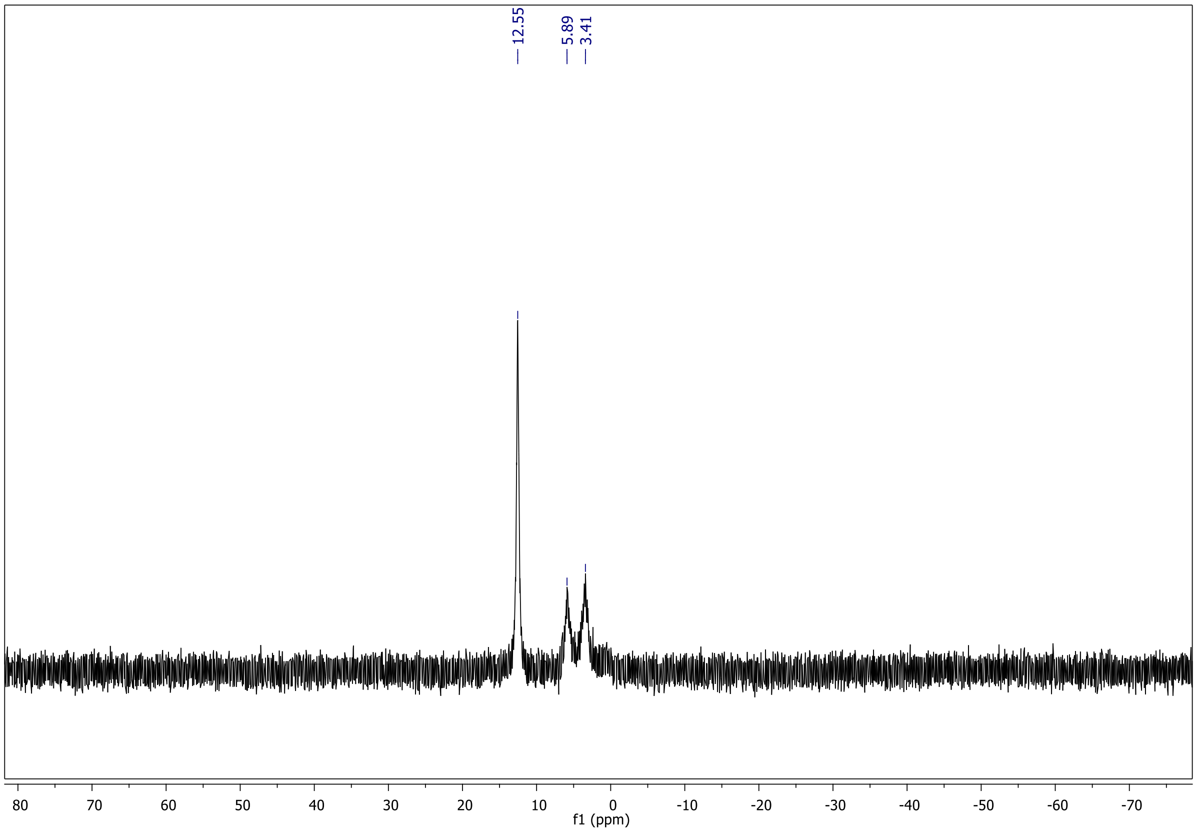
12.55
5.89
3.41
80
70
60
50
40
30
20
10
0
-10
-20
-30
-40
-50
-60
-70
f1 (ppm)

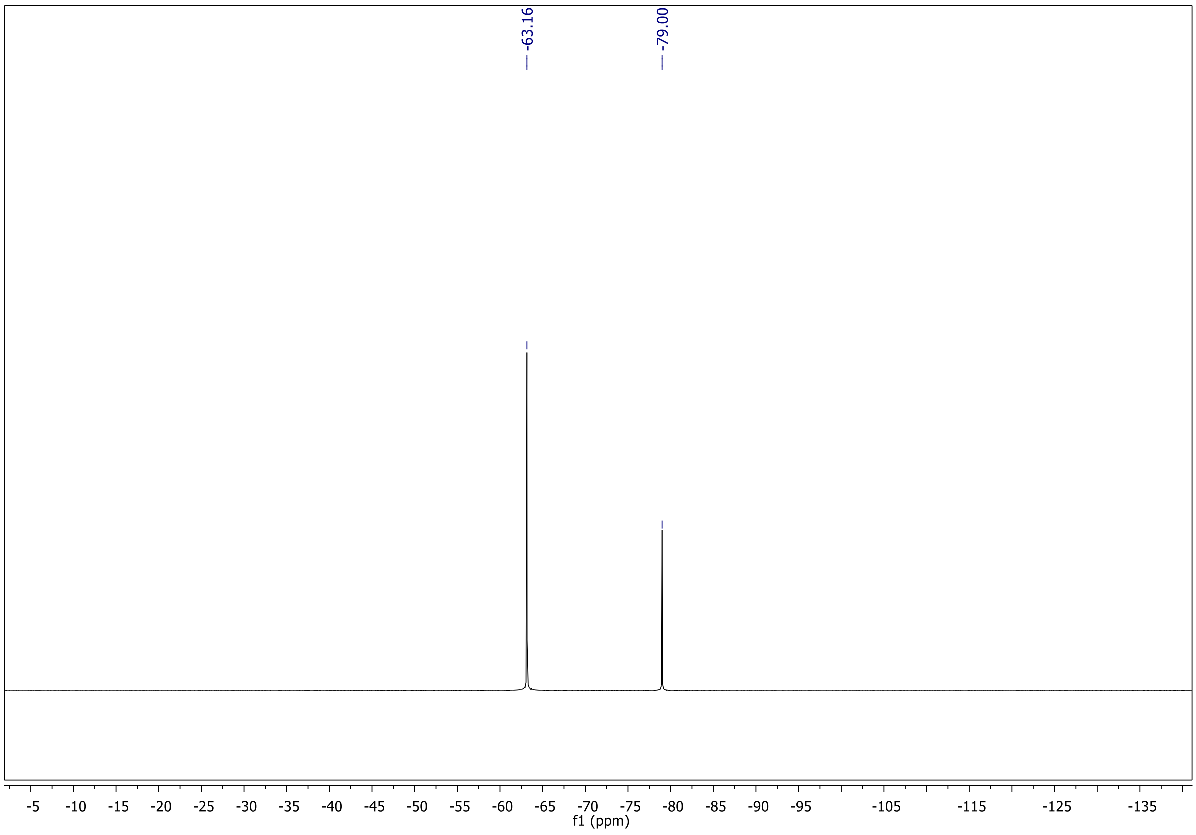
-63.16
-79.00
-5
-10
-15
-20
-25
-30
-35
-40
-45
-50
-55
-60
-65
-70
-75
-80
-85
-90
-95
-105
-115
-125
-135
f1 (ppm)

Figure S3. ^{1}H NMR spectrum (DMSO-d_6) for complex **C1a**.

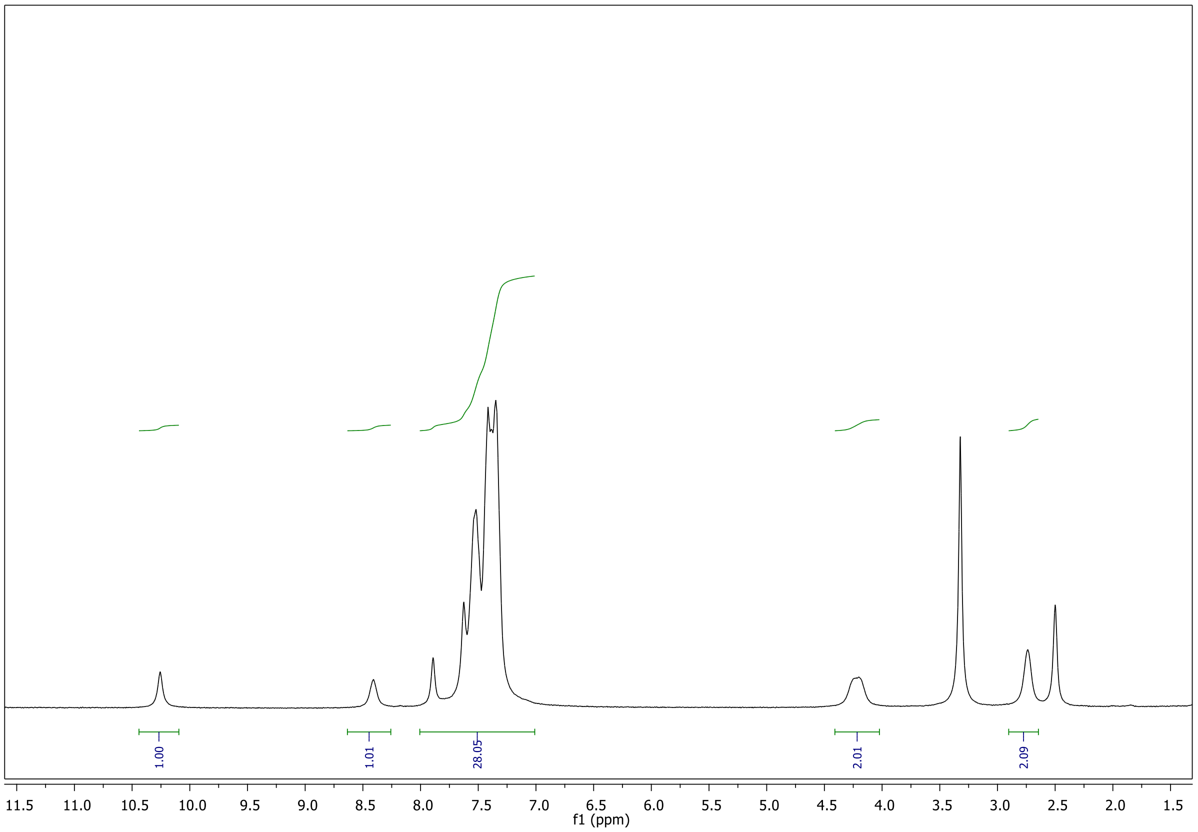

Figure S4. Spectra of ^{1}H, ^{13}C-APT, ^{31}P{^{1}H} and ^{19}F NMR spectra (CD_2Cl_2) for complex **C1b**.

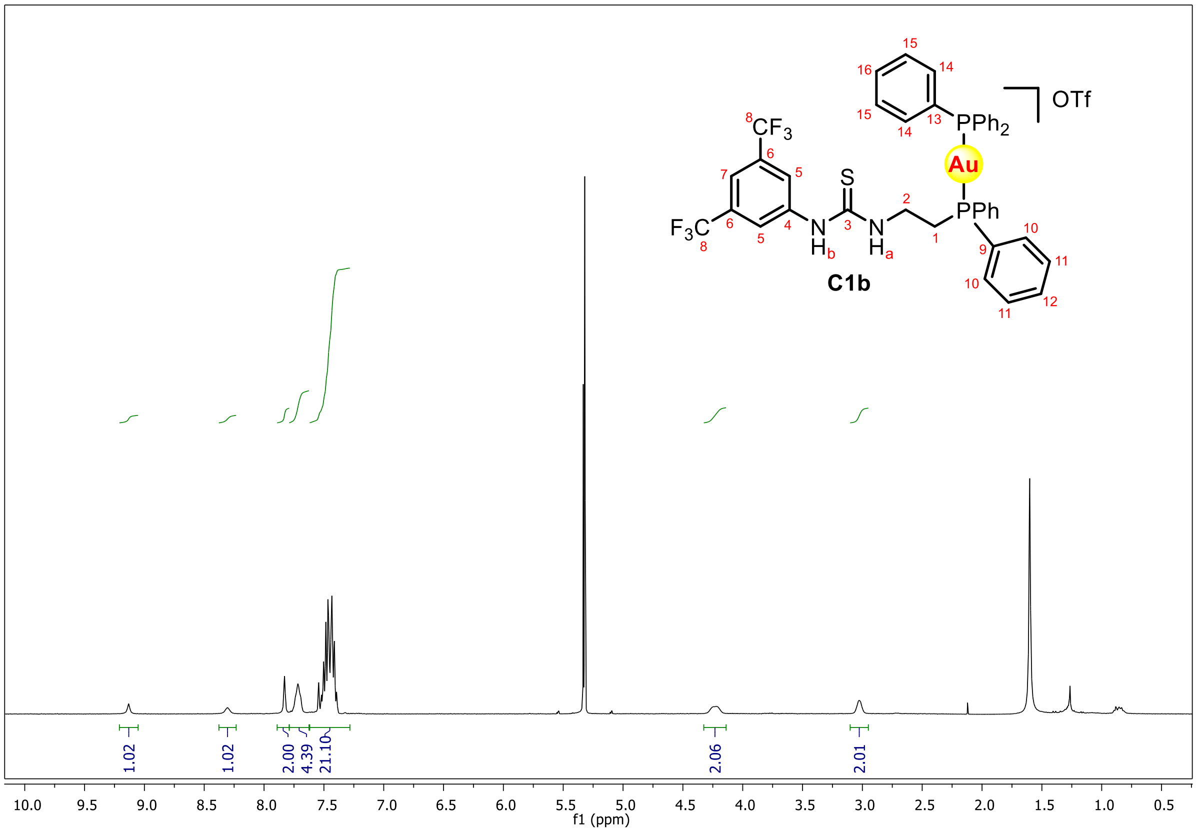

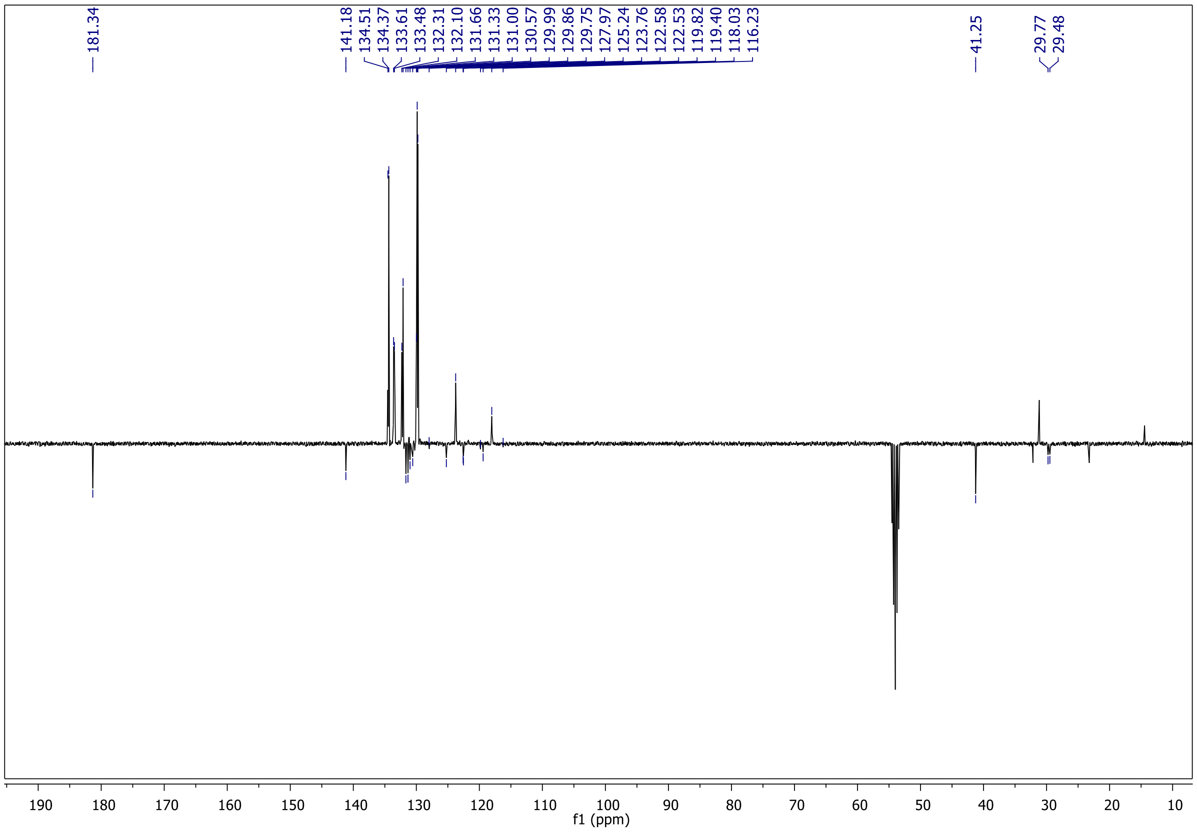
181.34
141.18
134.51
134.37
133.61
133.48
132.31
132.10
131.66
131.33
131.00
130.57
129.99
129.86
129.75
127.97
125.24
123.76
122.58
122.53
119.82
119.40
118.03
116.23
41.25
29.77
29.48
190 180 170 160 150 140 130 120 110 100 90 80 70 60 50 40 30 20 10
f1 (ppm)

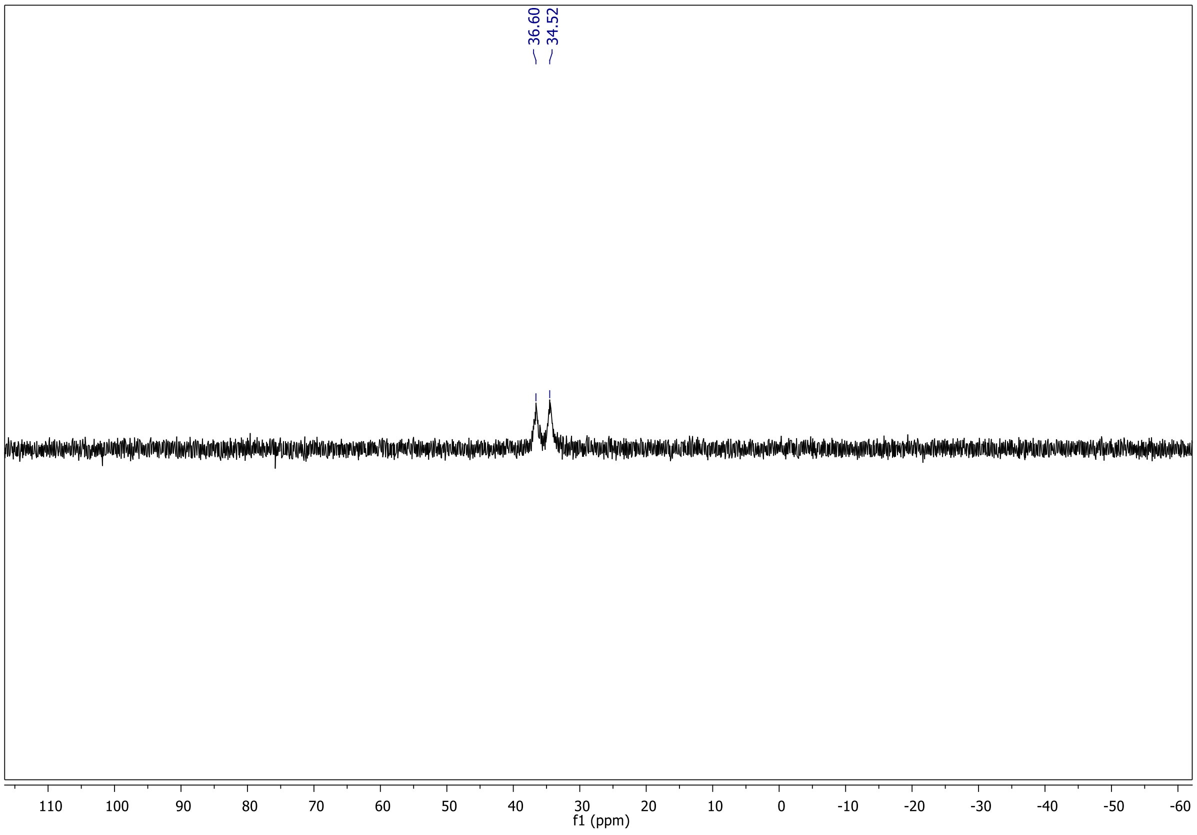
36.60
34.52
110 100 90 80 70 60 50 40 30 20 10 0 -10 -20 -30 -40 -50 -60
f1 (ppm)

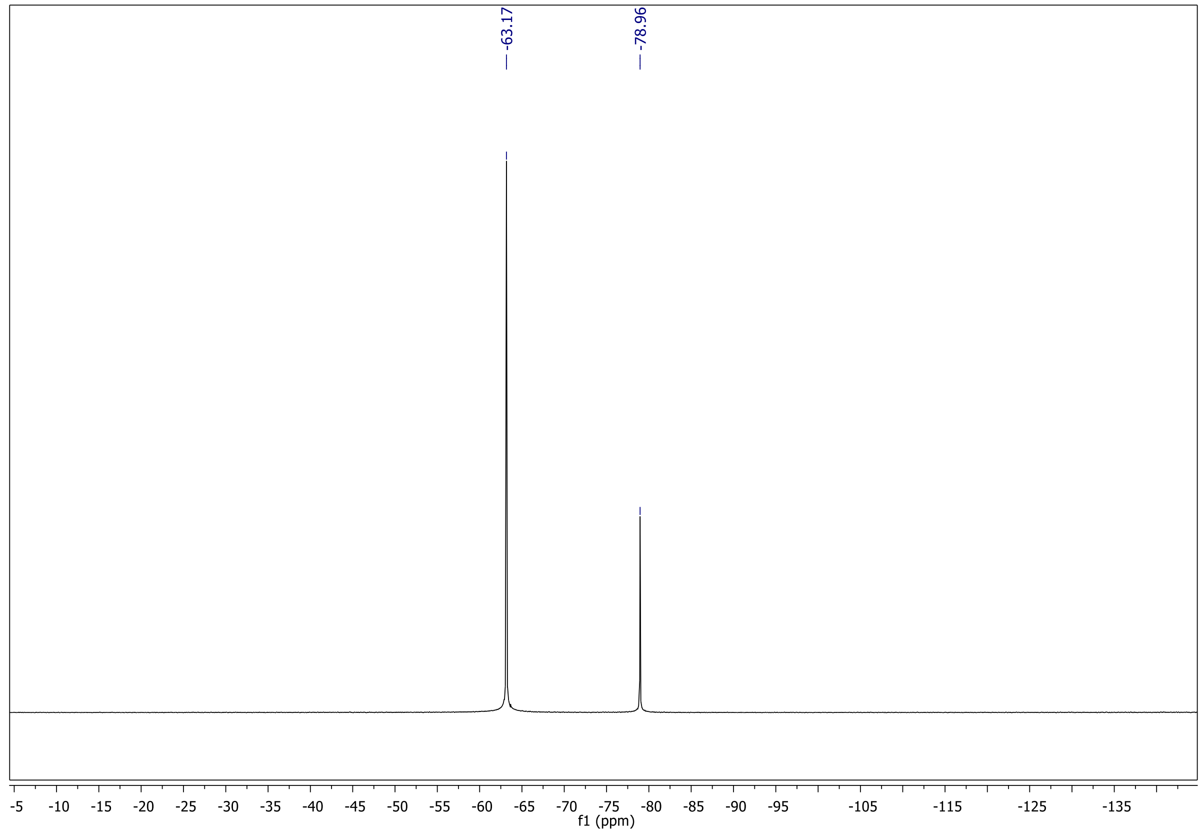

Figure S5. Spectra of ^{1}H, ^{13}C-APT, ^{31}P{^{1}H} and ^{19}F NMR spectra (CD_2Cl_2) for complex **C1c**.

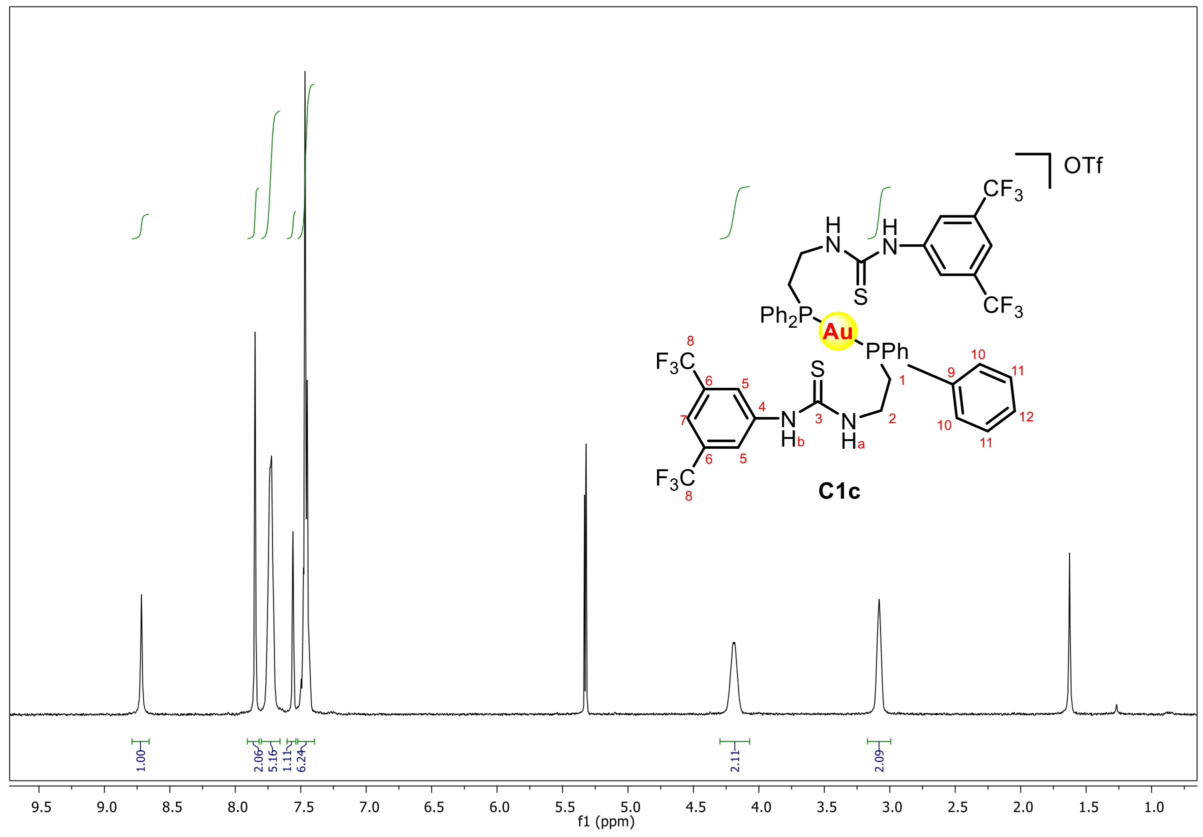

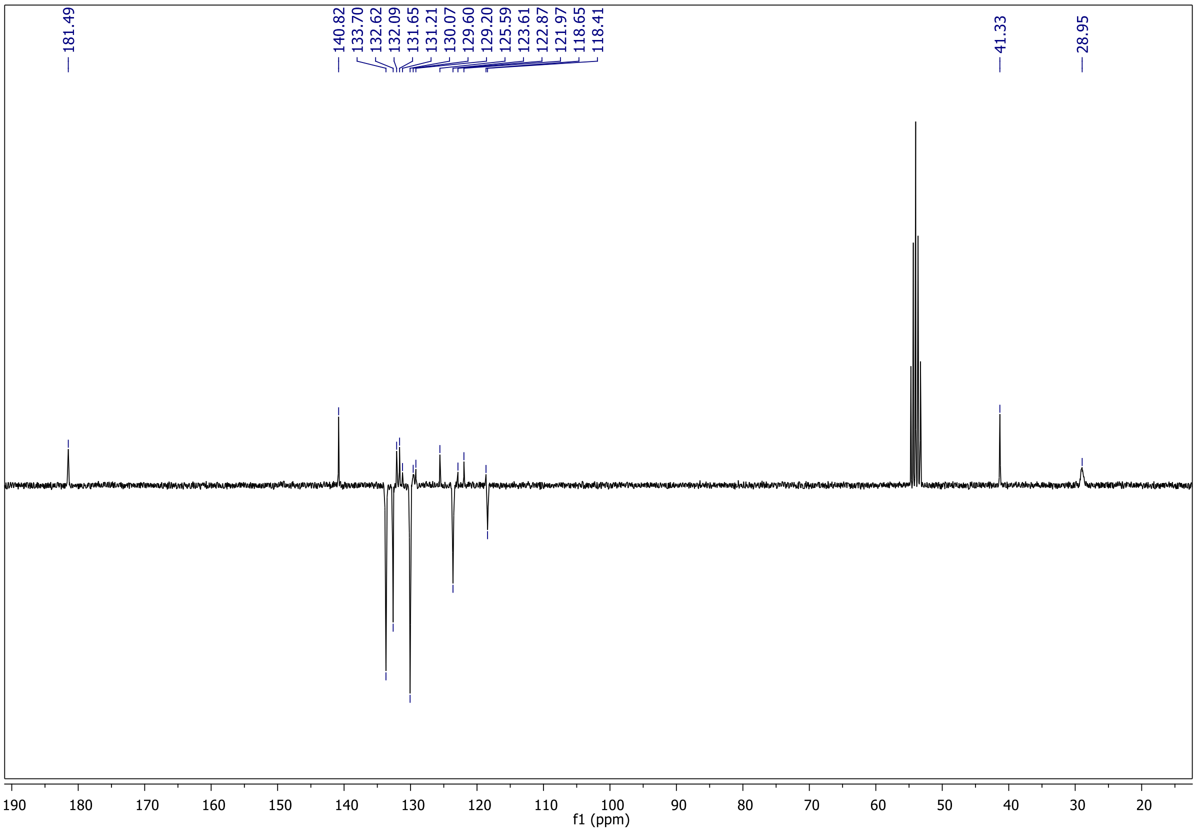
181.49
140.82
133.70
132.62
132.09
131.65
131.21
130.07
129.60
129.20
125.59
123.61
122.87
121.97
118.65
118.41
41.33
28.95
190 180 170 160 150 140 130 120 110 100 90 80 70 60 50 40 30 20
f1 (ppm)

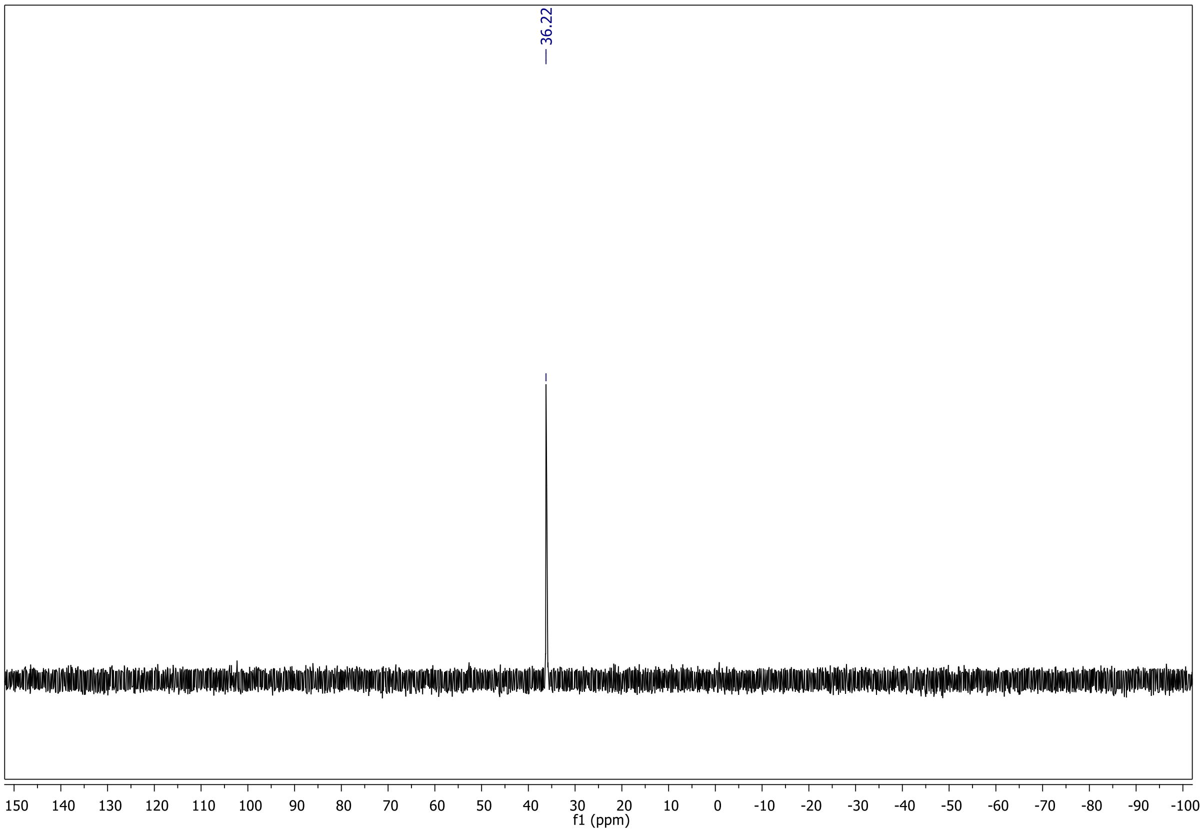
36.22
150 140 130 120 110 100 90 80 70 60 50 40 30 20 10 0 -10 -20 -30 -40 -50 -60 -70 -80 -90 -100
f1 (ppm)

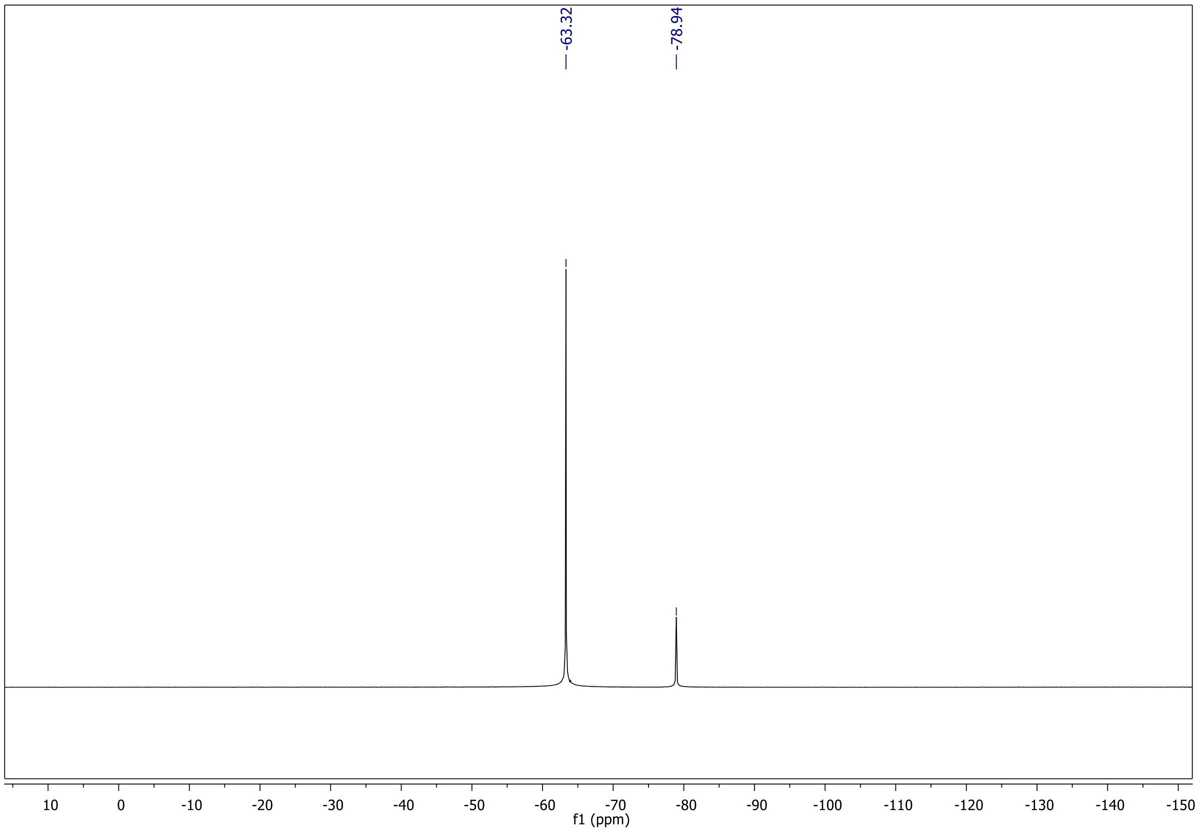

Figure S6. ^{1}H, ^{13}C-APT, $^{31}P\{^{1}H\}$ and ^{19}F NMR spectra (CD_2Cl_2) for complex **C1d**.

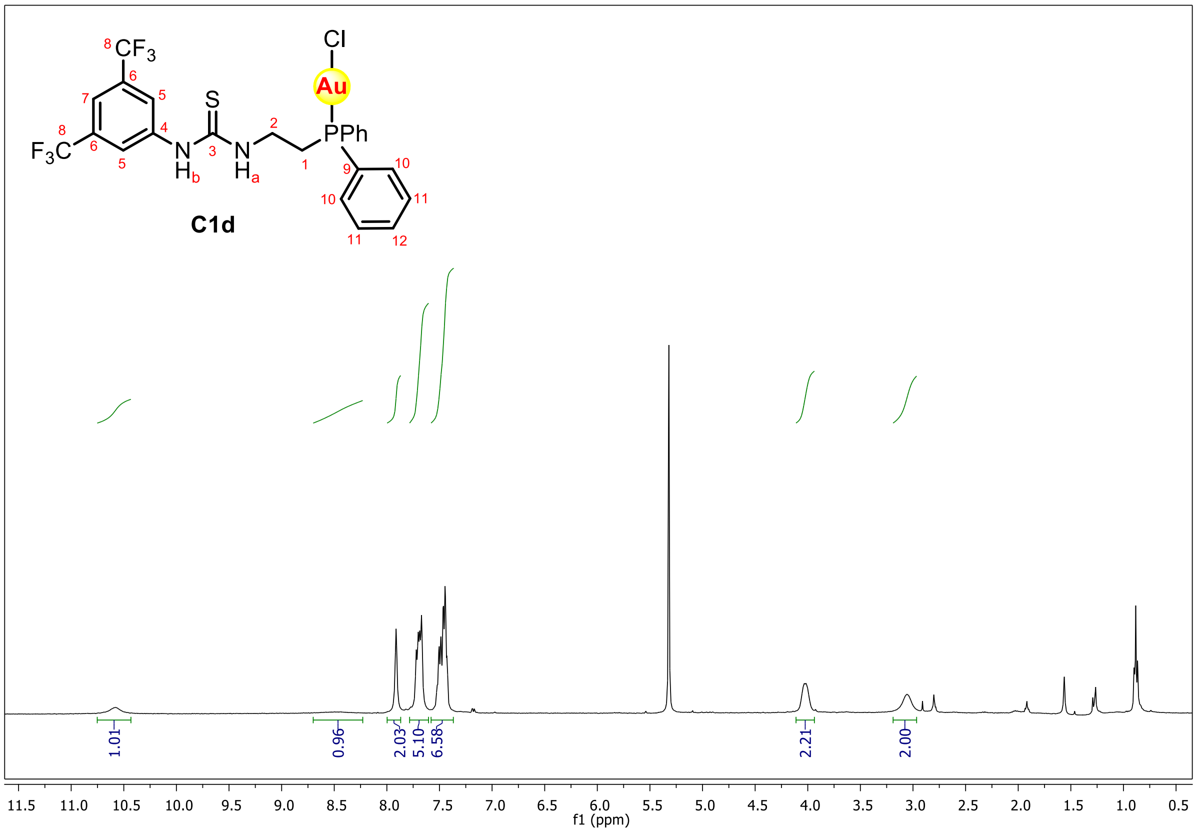

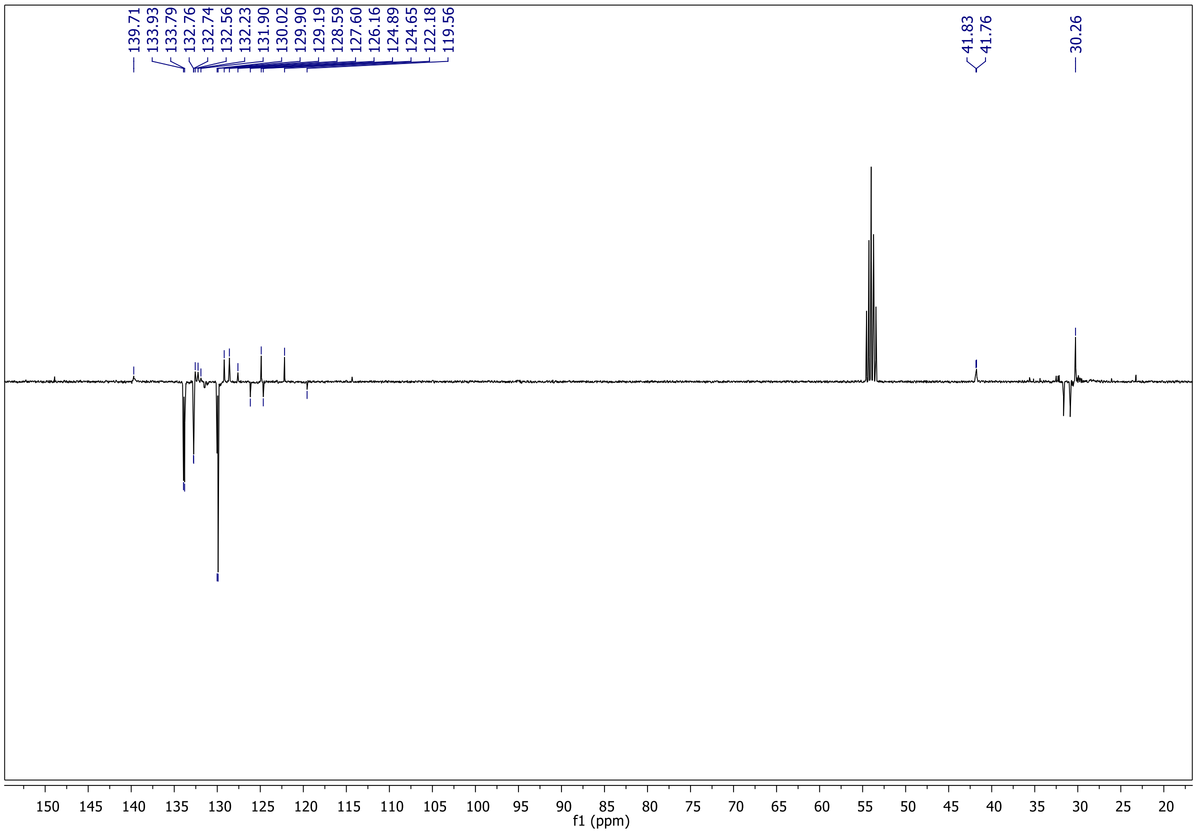
139.71
133.93
133.79
132.76
132.74
132.56
132.23
131.90
130.02
129.90
129.19
128.59
127.60
126.16
124.89
124.65
122.18
119.56
41.83
41.76
30.26
f1 (ppm)

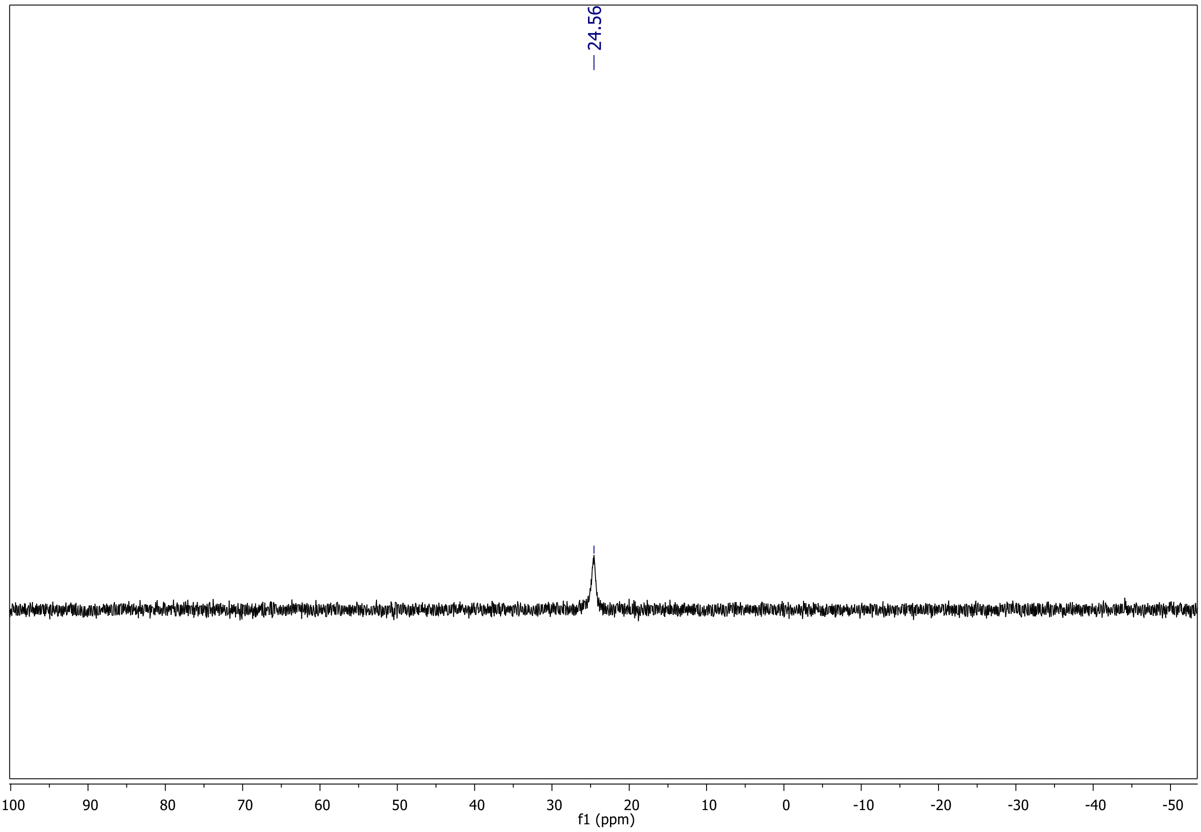
24.56
f1 (ppm)

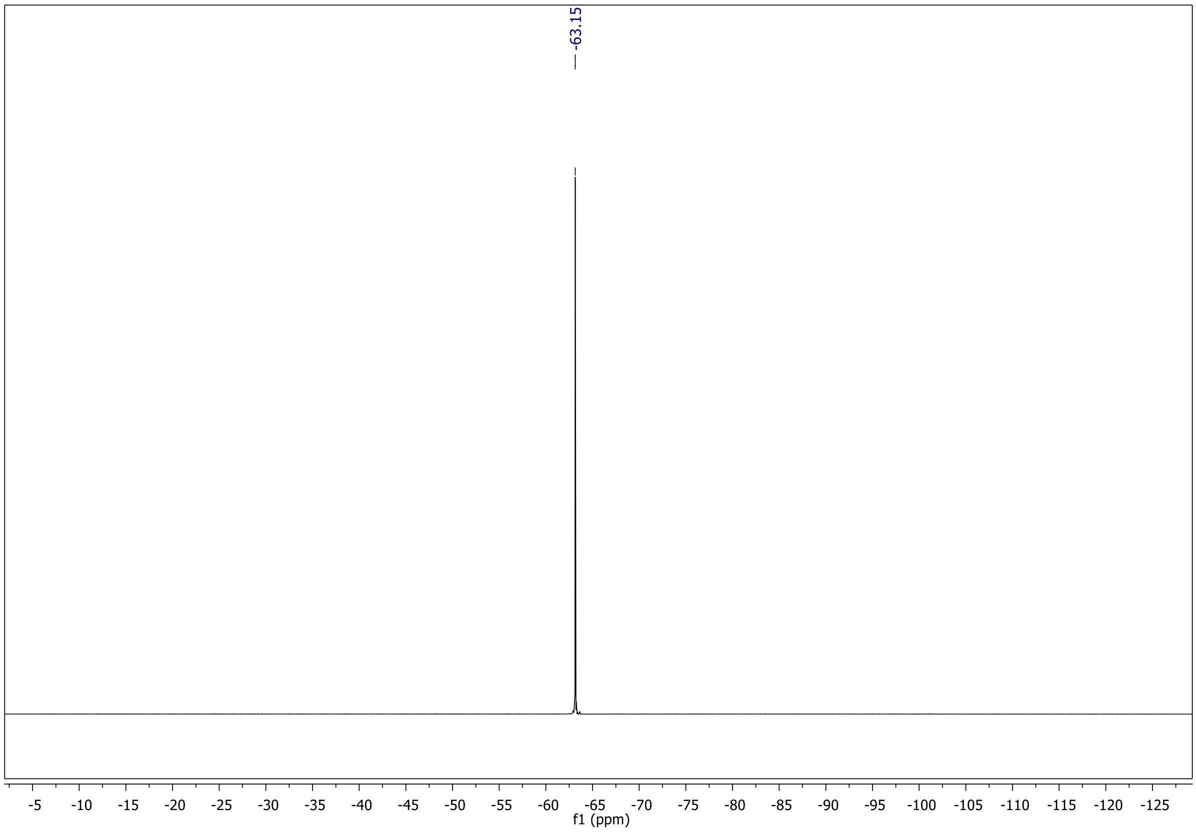

Figure S7. ^{1}H, ^{13}C-APT, $^{31}P\{^{1}H\}$ and ^{19}F NMR spectra (CD_2Cl_2) of complex **C1e**.

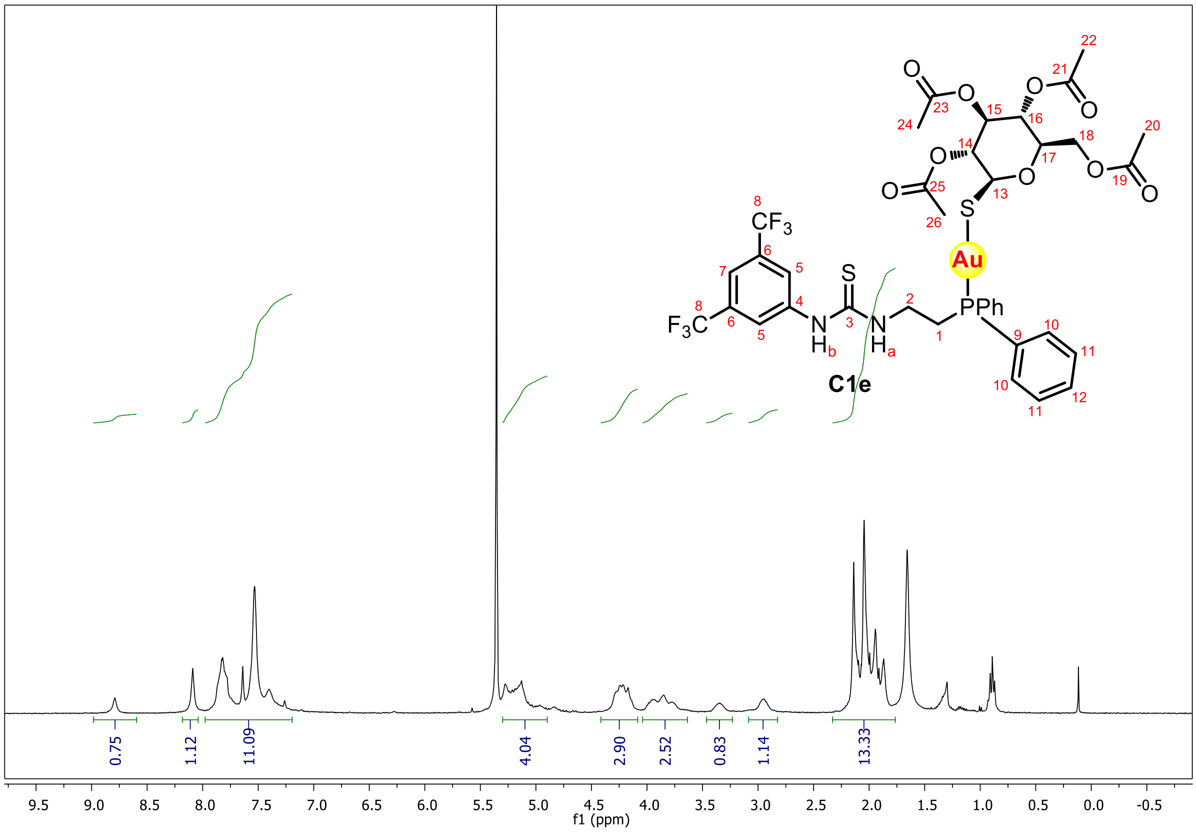

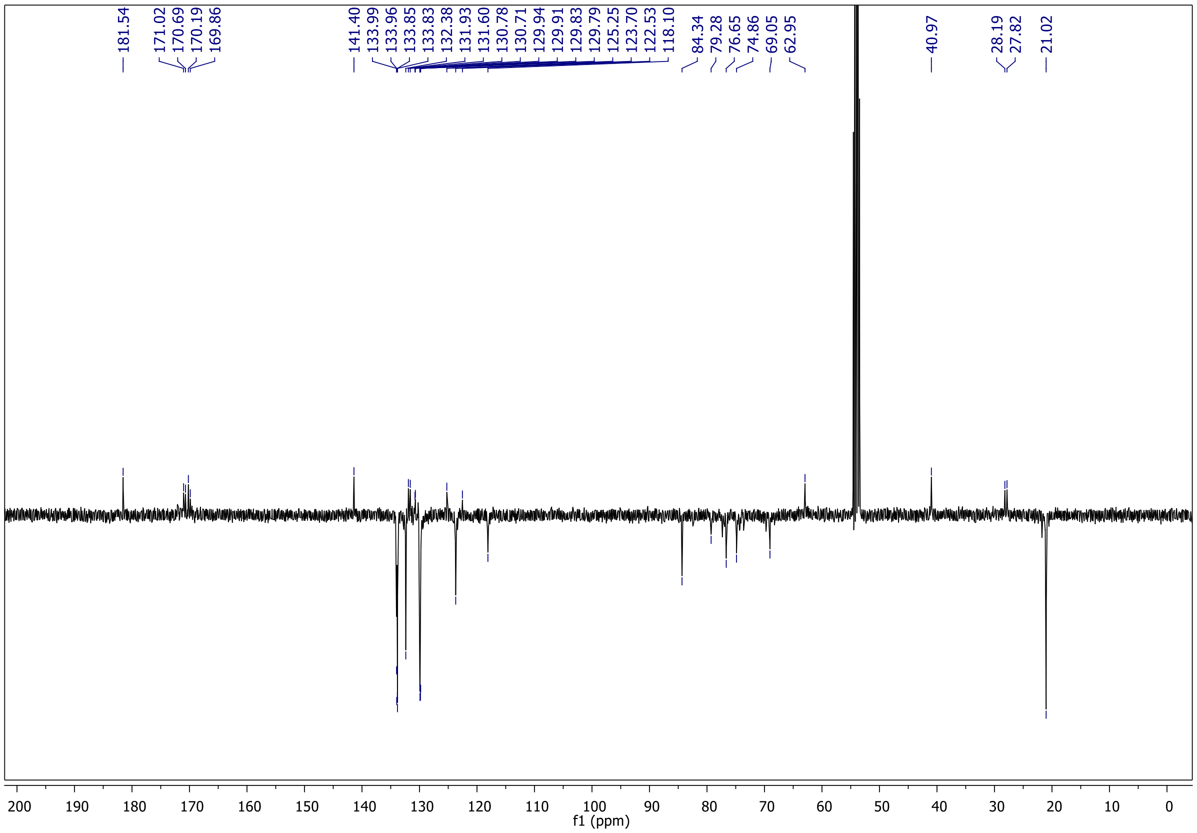
181.54
171.02
170.69
170.19
169.86
141.40
133.99
133.96
133.85
133.83
132.38
131.93
131.60
130.78
130.71
129.94
129.91
129.83
129.79
125.25
123.70
122.53
118.10
84.34
79.28
76.65
74.86
69.05
62.95
40.97
28.19
27.82
21.02
200 190 180 170 160 150 140 130 120 110 100 90 80 70 60 50 40 30 20 10 0
f1 (ppm)

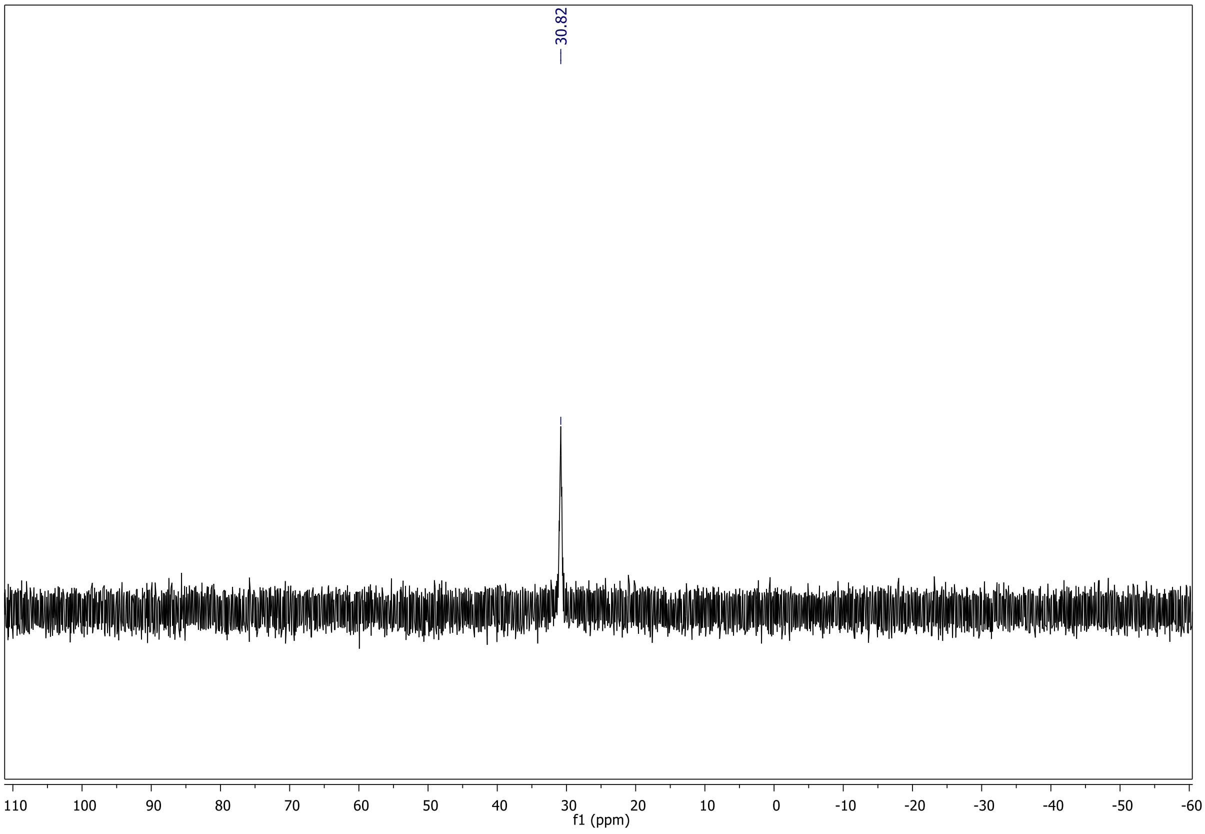
30.82
110 100 90 80 70 60 50 40 30 20 10 0 -10 -20 -30 -40 -50 -60
f1 (ppm)

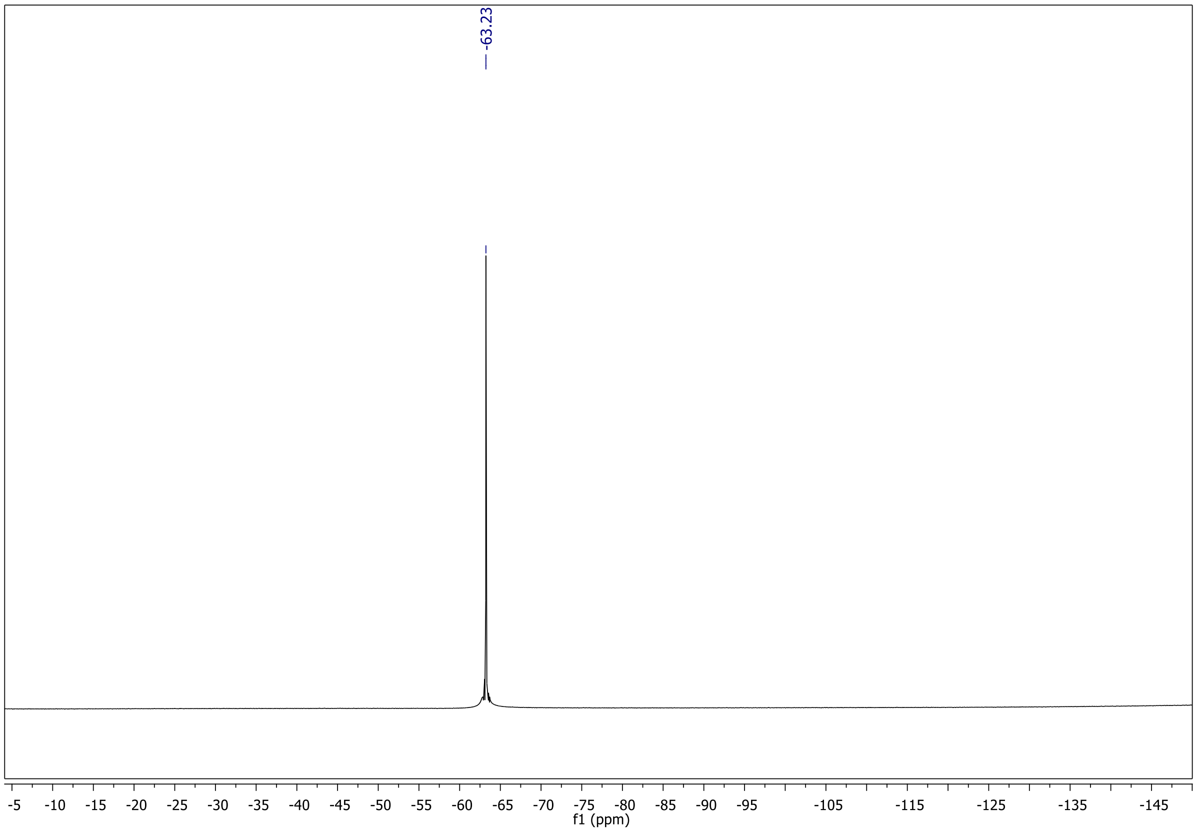

Figure S8. ^{1}H, ^{13}C-APT and ^{31}P{^{1}H} NMR spectra (CD_2Cl_2) of **T2**.

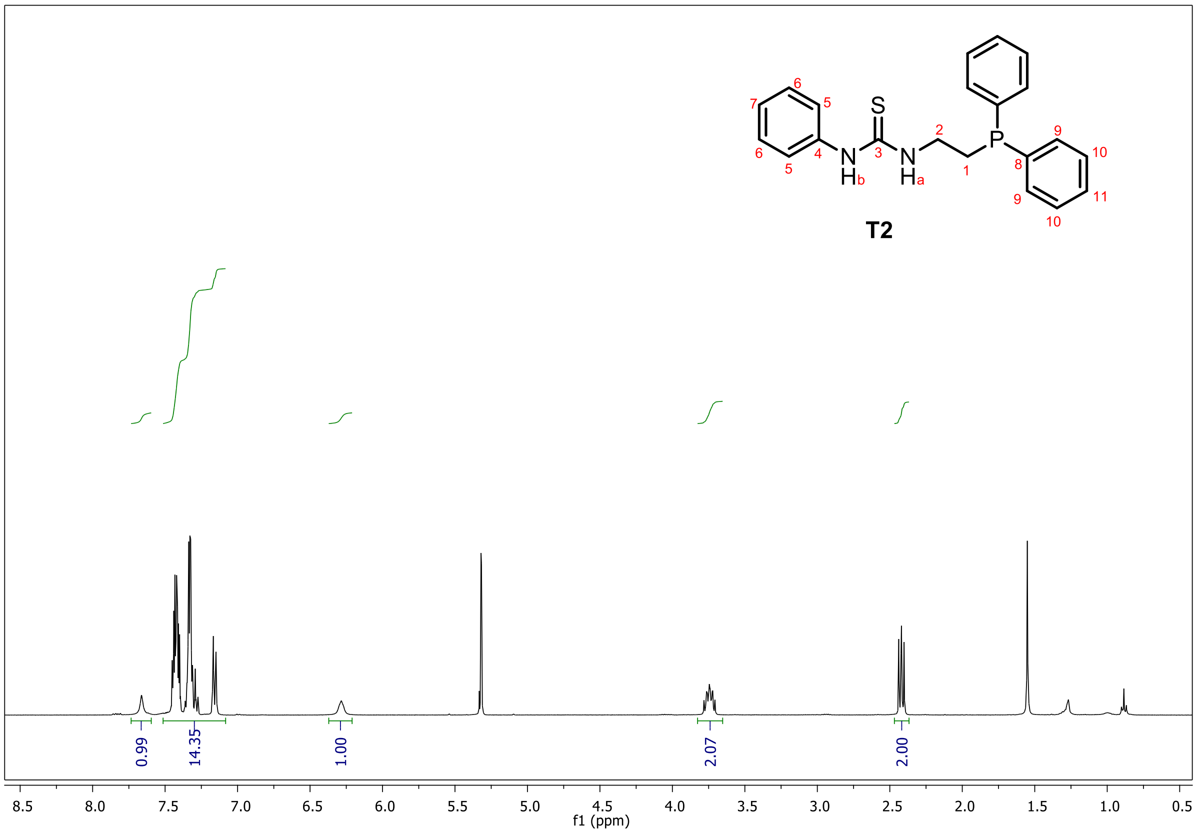

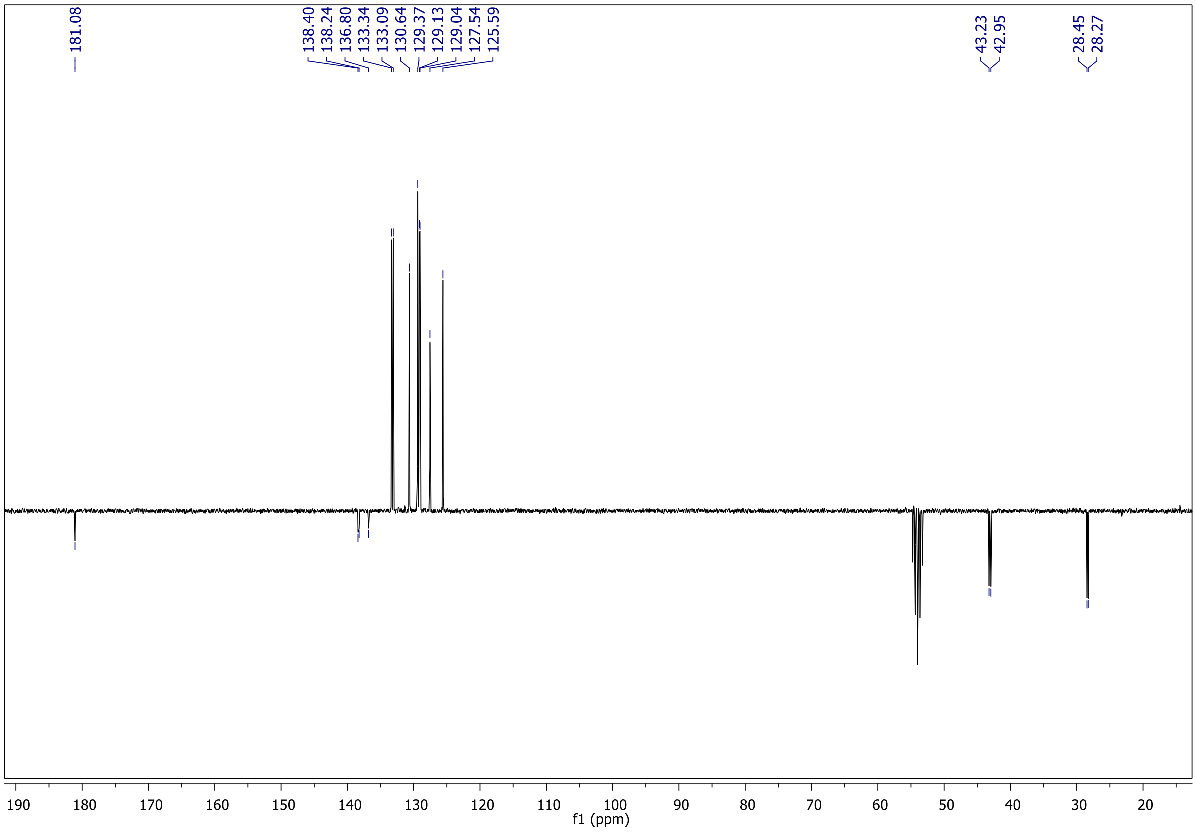
181.08
138.40
138.24
136.80
133.34
133.09
130.64
129.37
129.13
129.04
127.54
125.59
43.23
42.95
28.45
28.27
190 180 170 160 150 140 130 120 110 100 90 80 70 60 50 40 30 20
f1 (ppm)

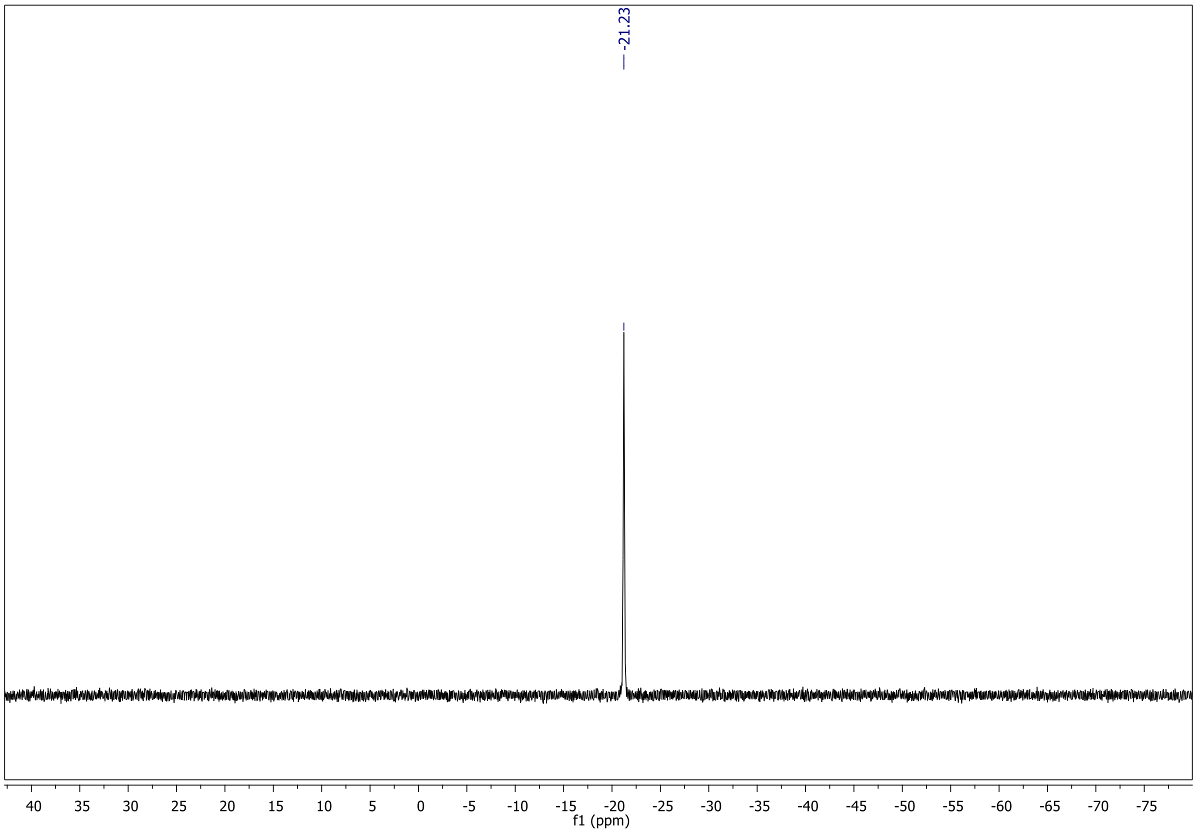
-21.23
40 35 30 25 20 15 10 5 0 -5 -10 -15 -20 -25 -30 -35 -40 -45 -50 -55 -60 -65 -70 -75
f1 (ppm)

Figure S9. ^{1}H, ^{13}C-APT, $^{31}P\{^{1}H\}$ and ^{19}F NMR spectra (CD_2Cl_2) for complex **C2a**.

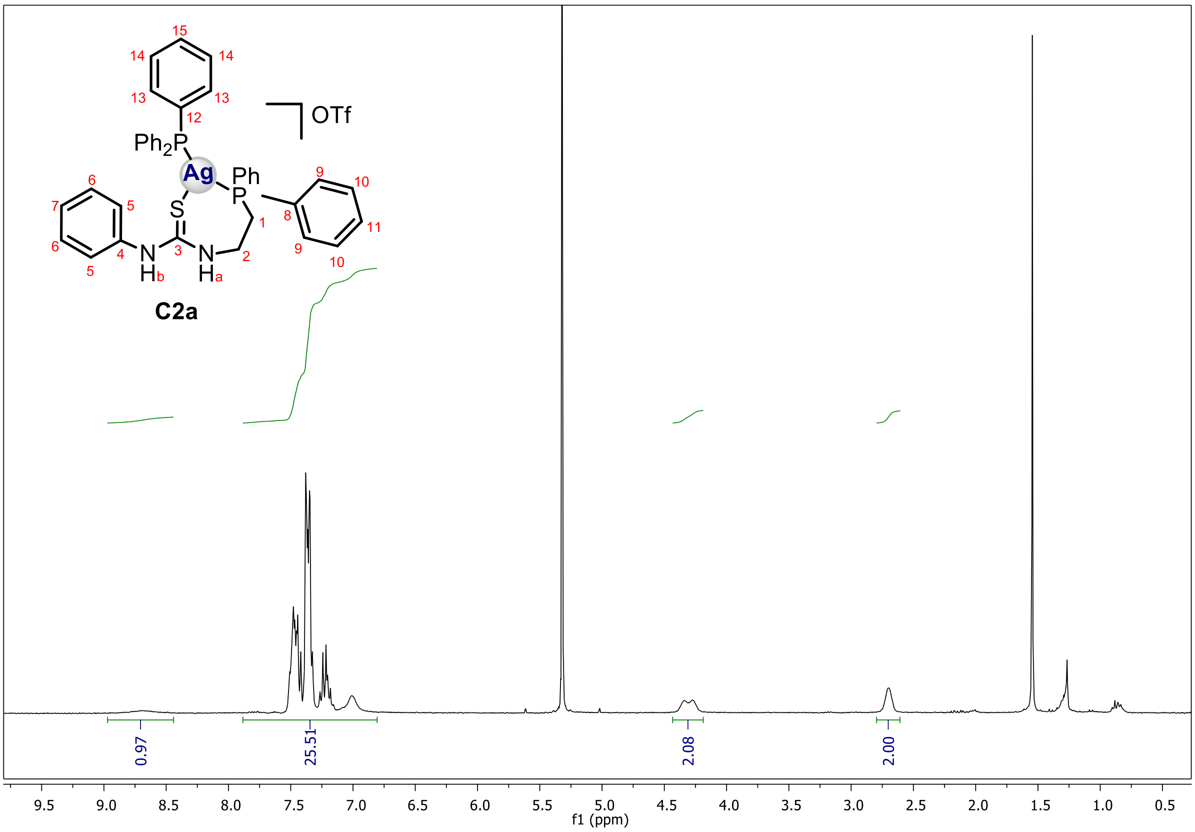

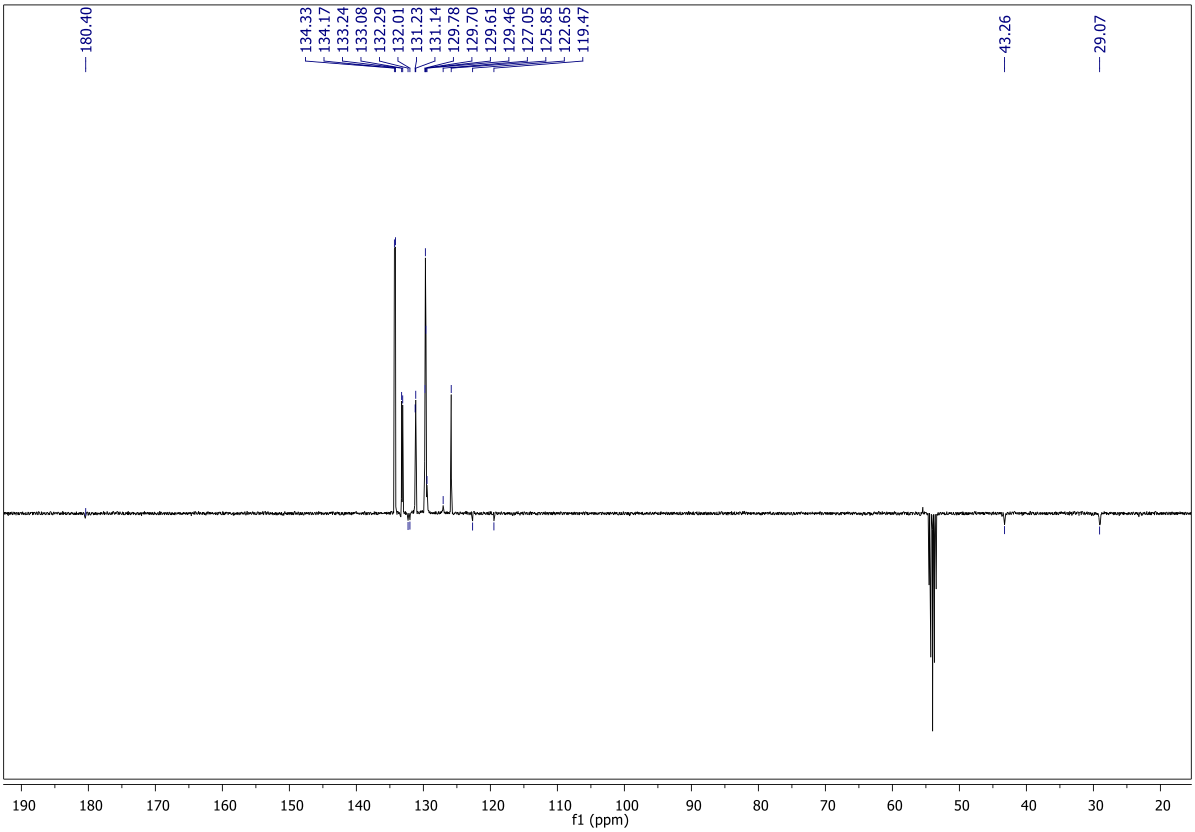

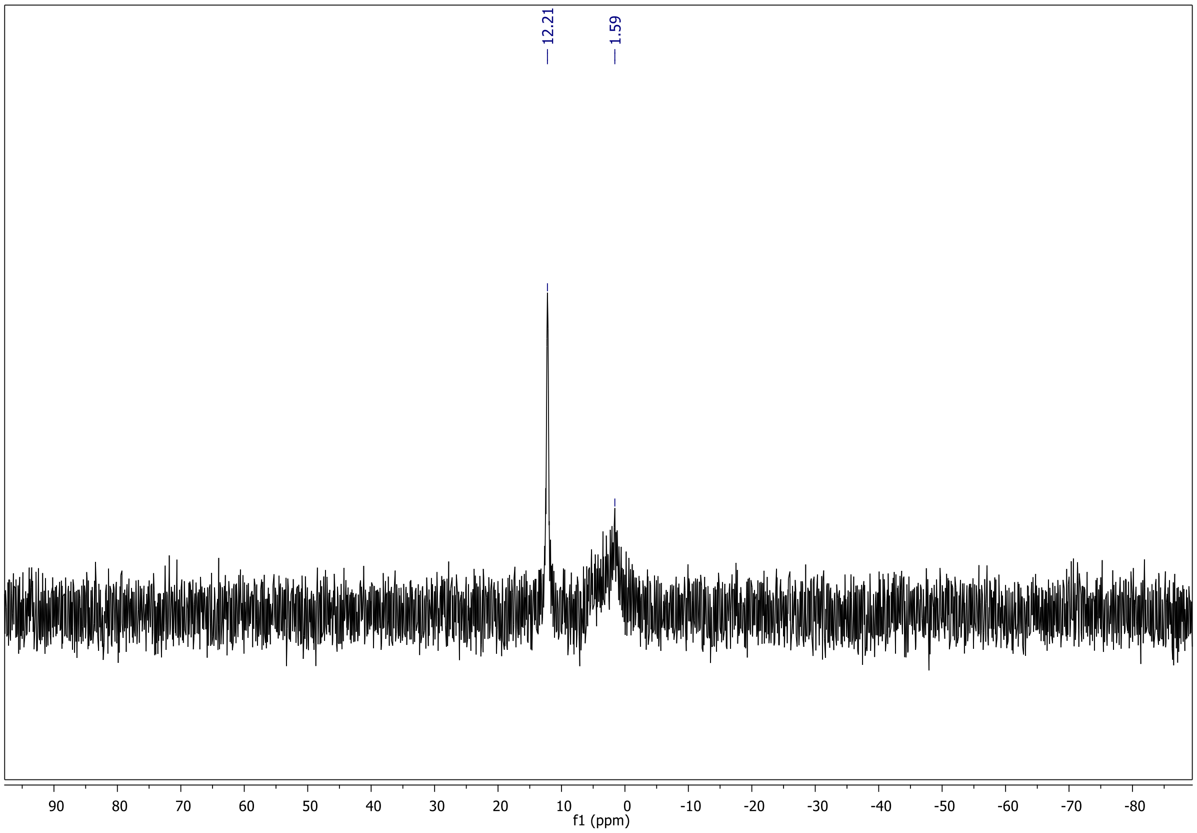
12.21
1.59
90
80
70
60
50
40
30
20
10
0
-10
-20
-30
-40
-50
-60
-70
-80
f1 (ppm)

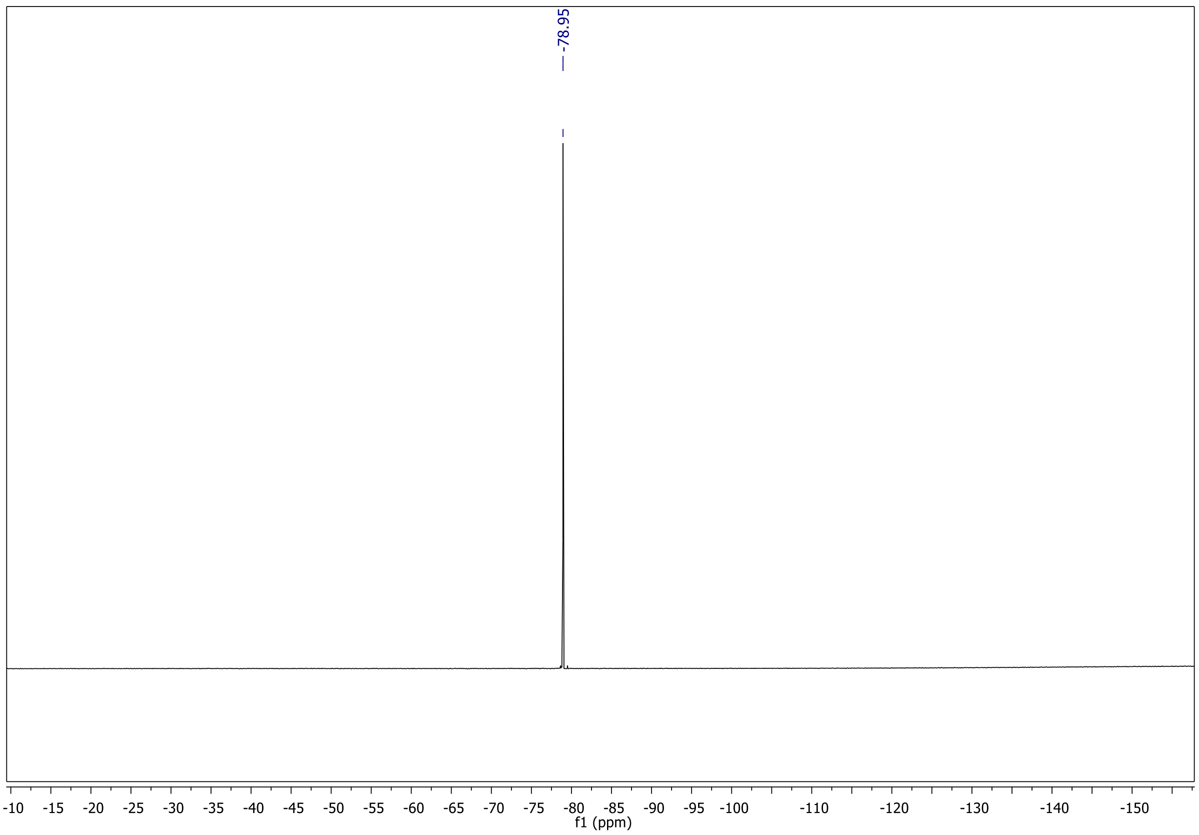
-78.95
-10
-15
-20
-25
-30
-35
-40
-45
-50
-55
-60
-65
-70
-75
-80
-85
-90
-95
-100
-110
-120
-130
-140
-150
f1 (ppm)

Figure S10. ^{1}H NMR spectrum (DMSO-d_6) for complex **C2a**.

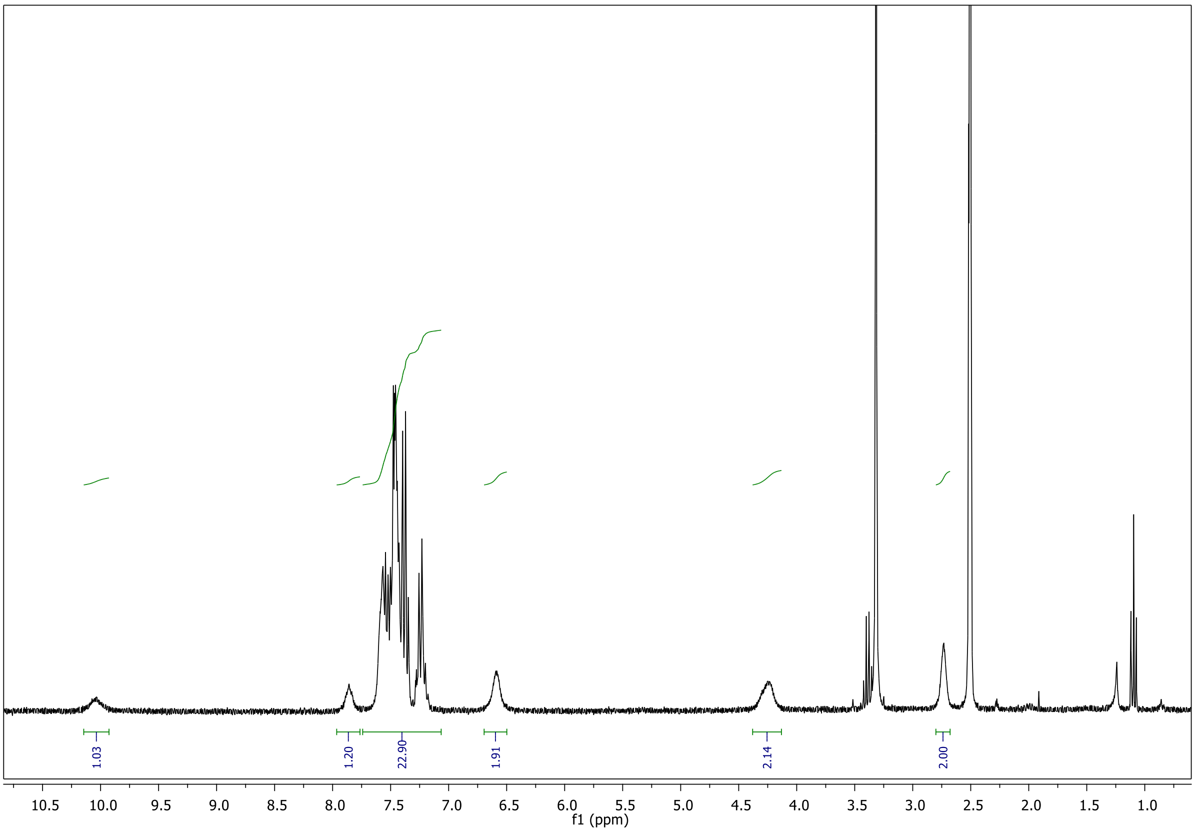

Figure S11. ^{1}H, ^{13}C-APT, ^{31}P{^{1}H} and ^{19}F NMR spectra (CD_2Cl_2) for complex **C2b**.

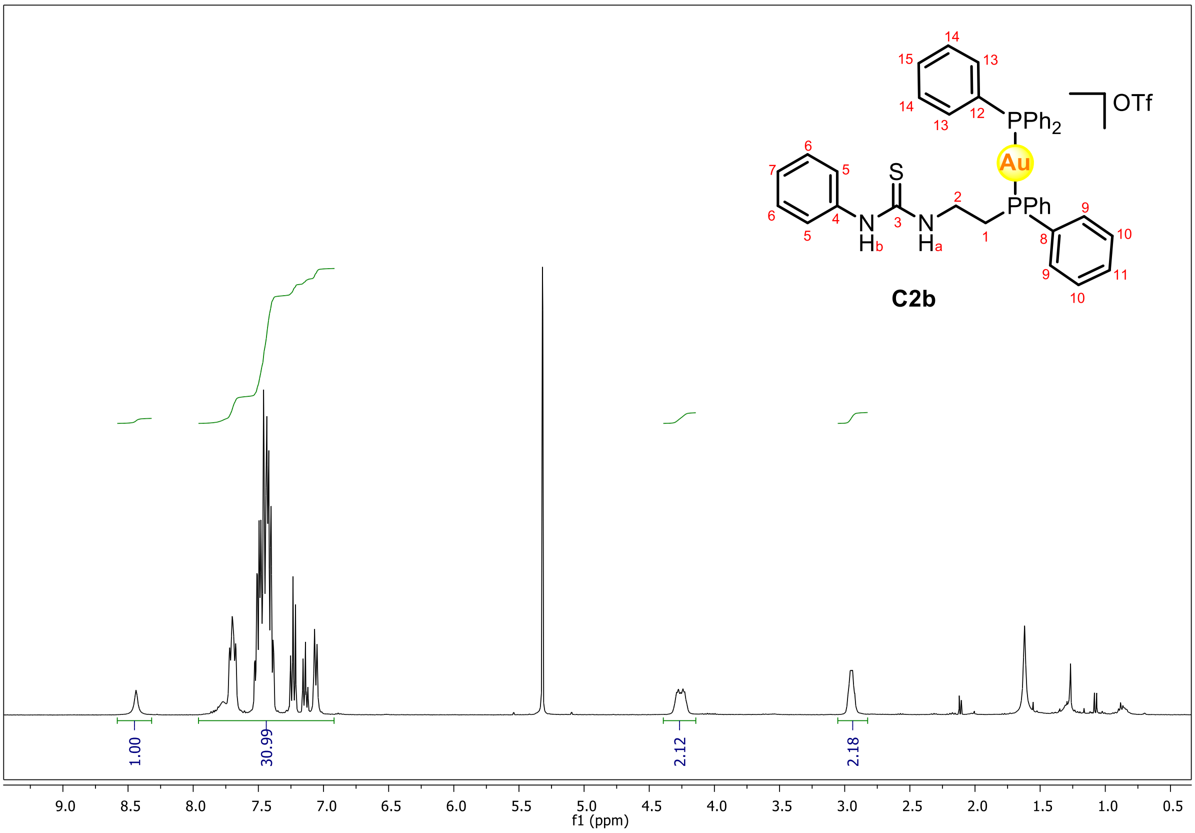

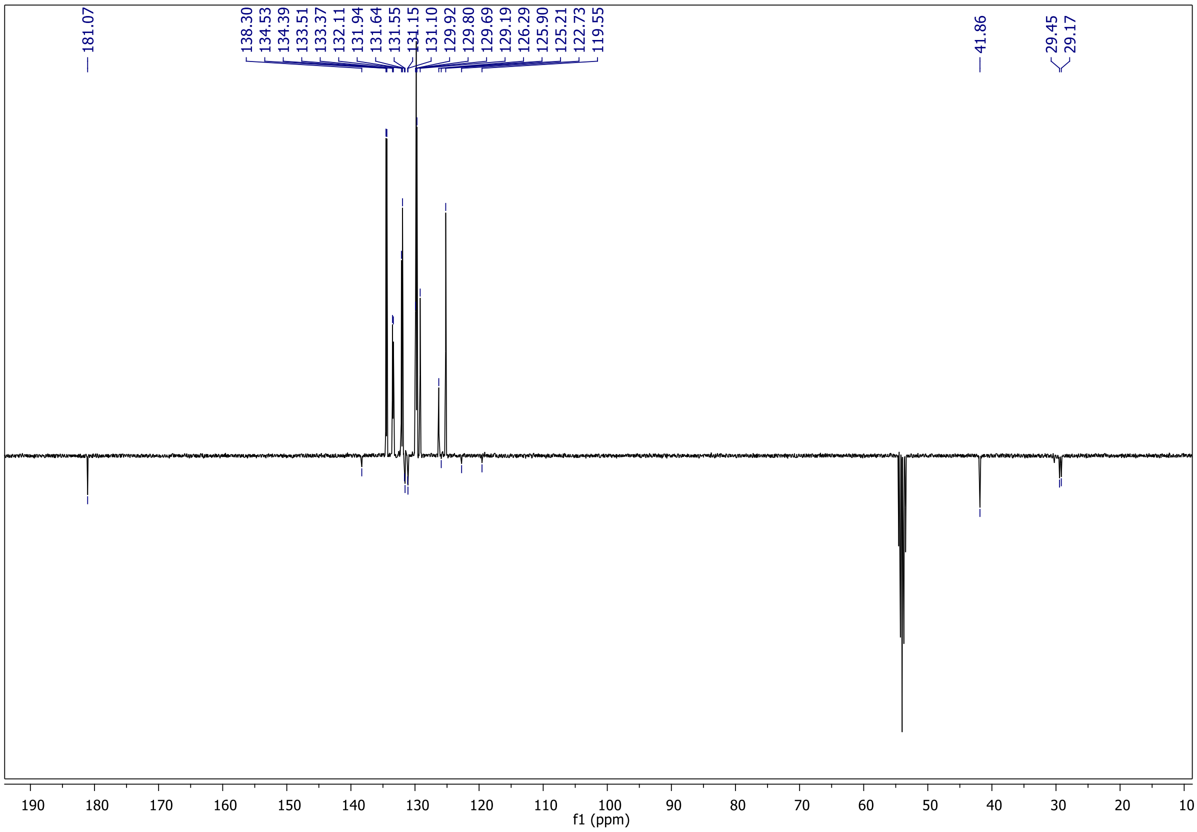
181.07
138.30
134.53
134.39
133.51
133.37
132.11
131.94
131.64
131.55
131.15
131.10
129.92
129.80
129.69
129.19
126.29
125.90
125.21
122.73
119.55
41.86
29.45
29.17
190 180 170 160 150 140 130 120 110 100 90 80 70 60 50 40 30 20 10
f1 (ppm)

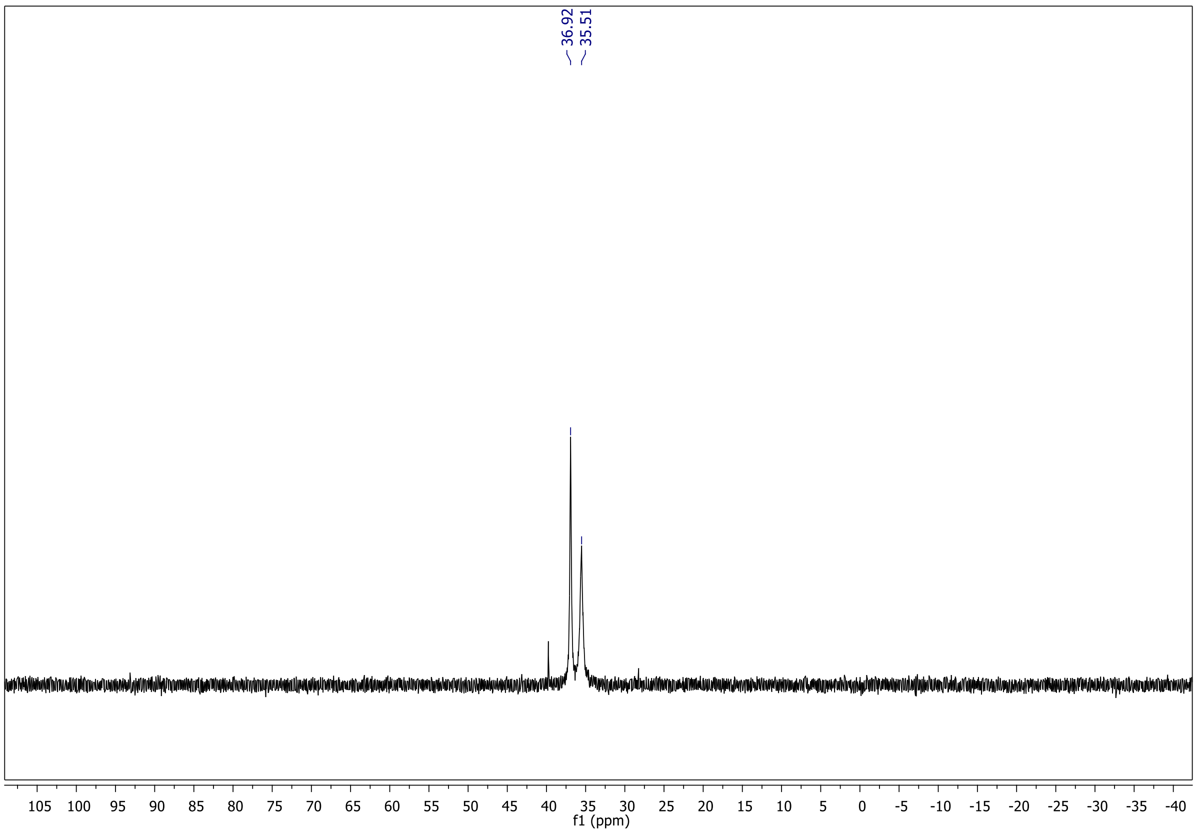
36.92
35.51
105 100 95 90 85 80 75 70 65 60 55 50 45 40 35 30 25 20 15 10 5 0 -5 -10 -15 -20 -25 -30 -35 -40
f1 (ppm)

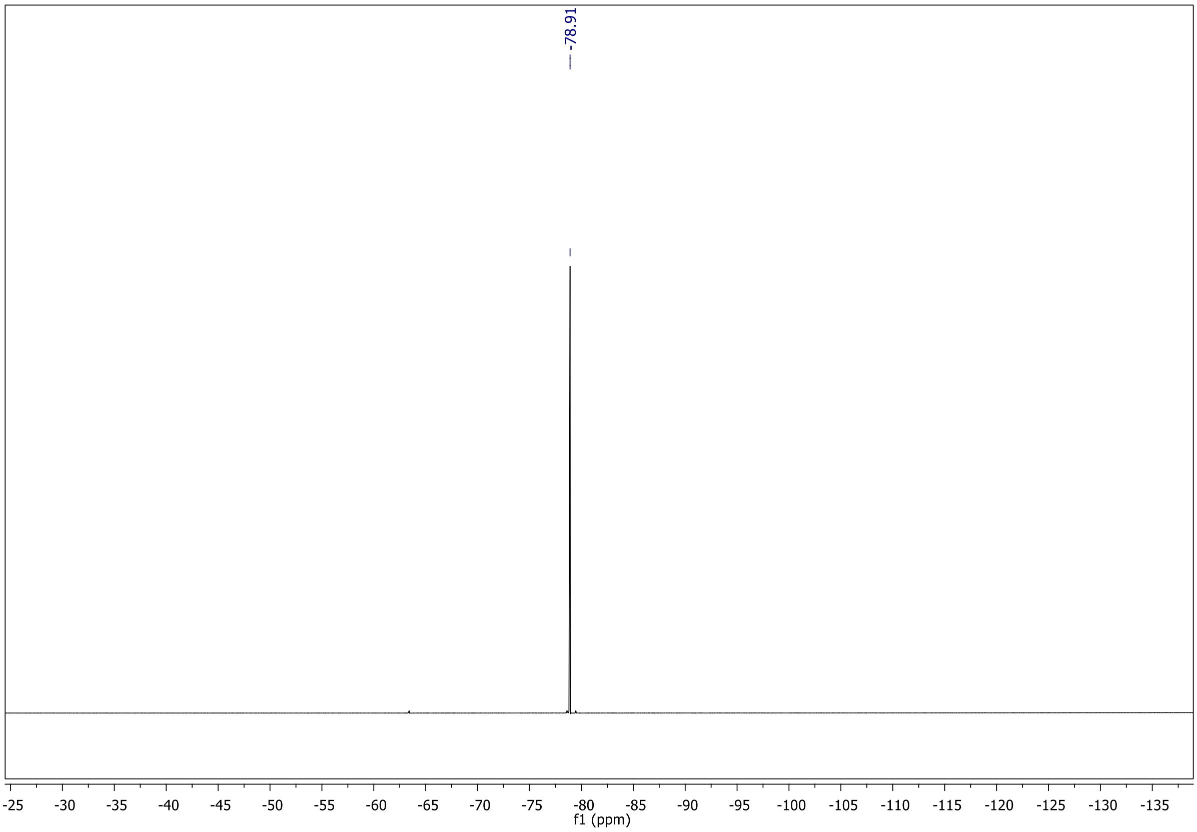

Figure S12. ^{1}H NMR spectrum (DMSO-d_6) for complex **C2b**.

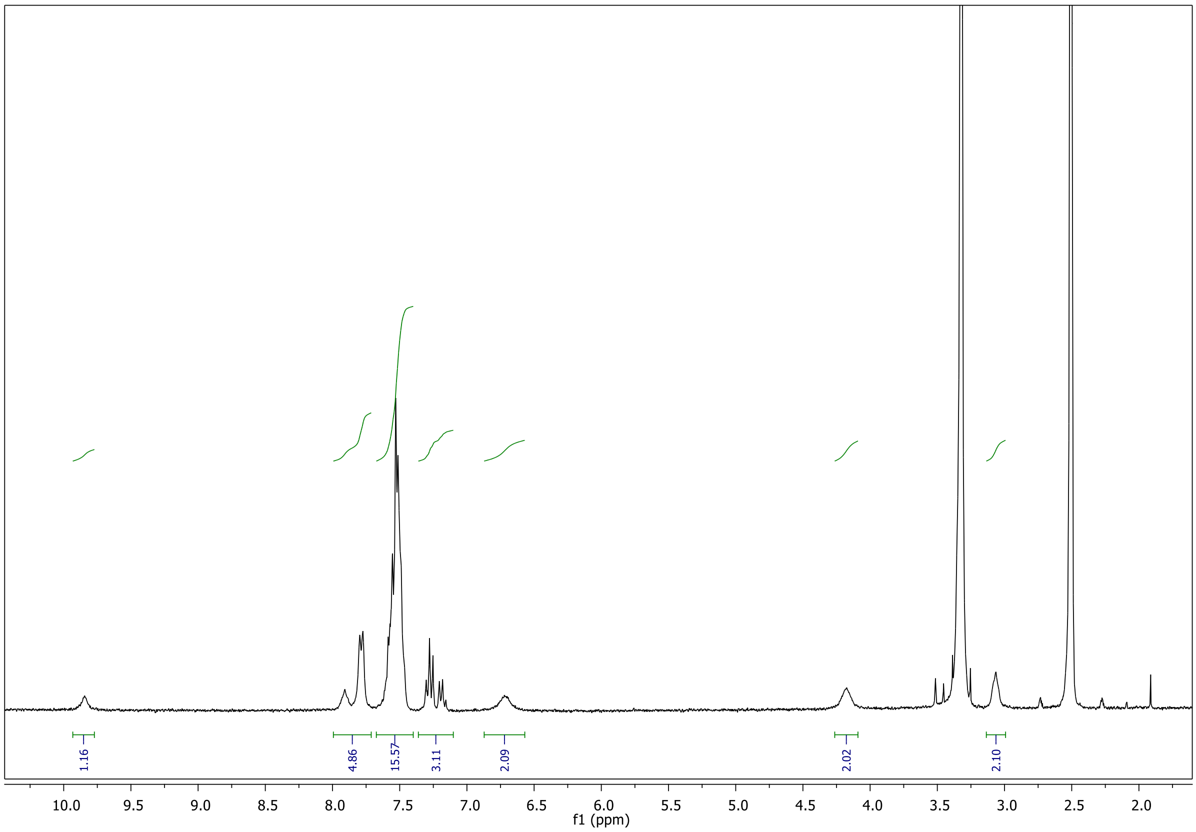

Figure S13. ^{1}H, ^{13}C-APT, $^{31}P\{^{1}H\}$ and ^{19}F NMR spectra (DMSO-d_6) for complex **C2c**.

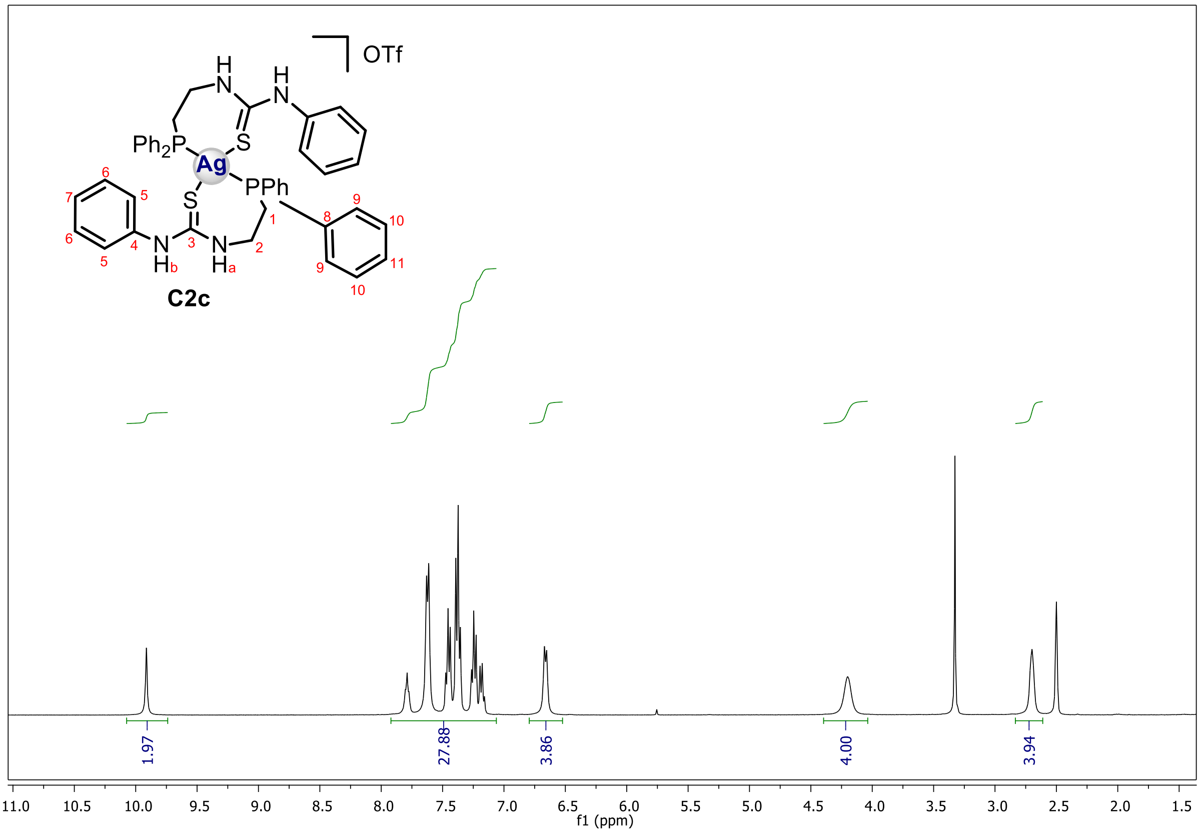

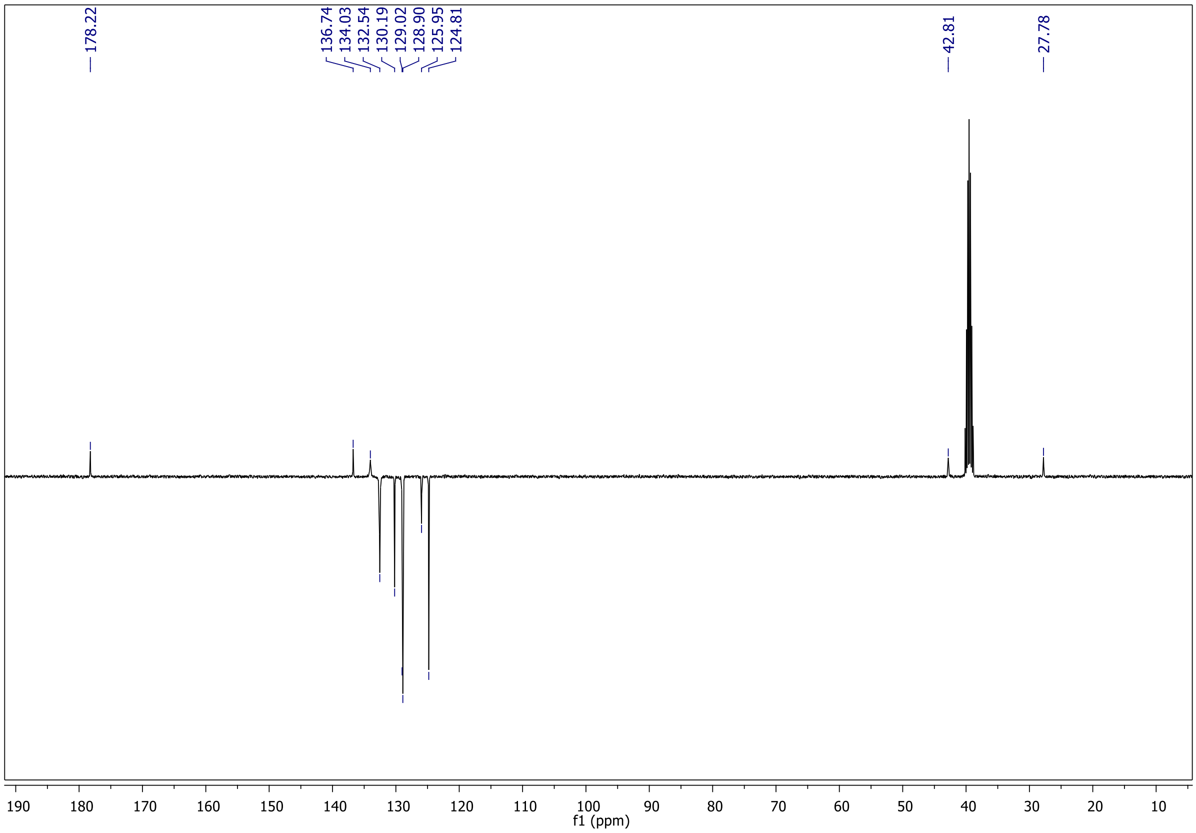

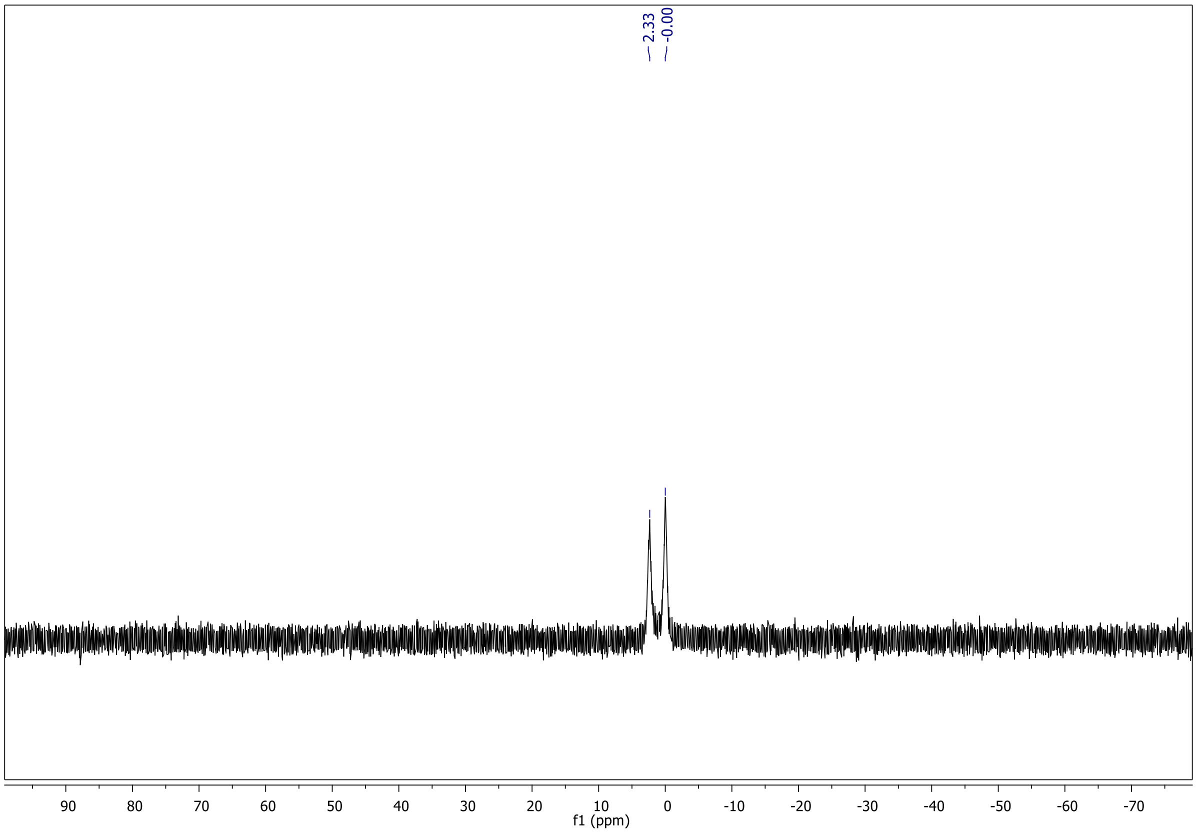
2.33
-0.00
90 80 70 60 50 40 30 20 10 0 -10 -20 -30 -40 -50 -60 -70
f1 (ppm)

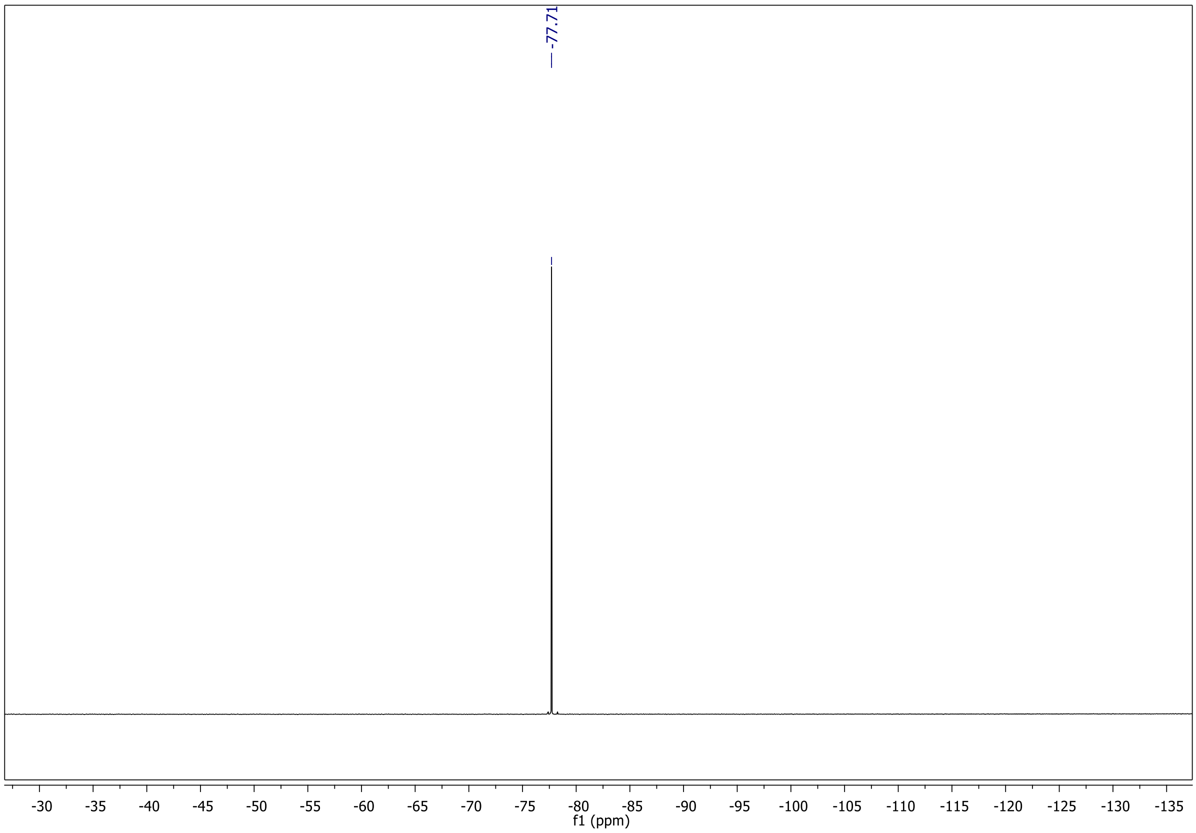
-77.71
-30 -35 -40 -45 -50 -55 -60 -65 -70 -75 -80 -85 -90 -95 -100 -105 -110 -115 -120 -125 -130 -135
f1 (ppm)

Figure S14. $^{31}P\{^1H\}$ NMR spectrum (CD_2Cl_2) at 193 K for complex **C1a**.

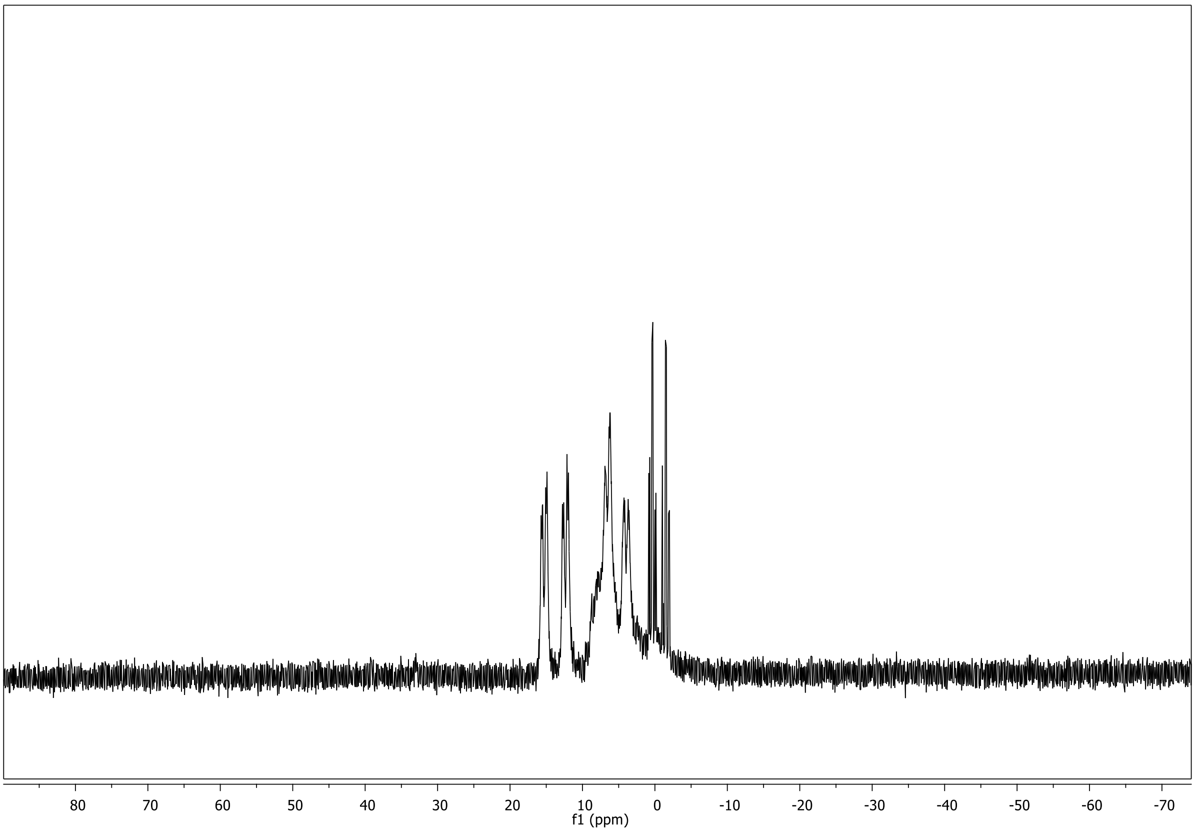

Figure S15. $^{31}P\{^1H\}$ NMR spectrum (CD_2Cl_2) at 193 K for complex **C1b**.

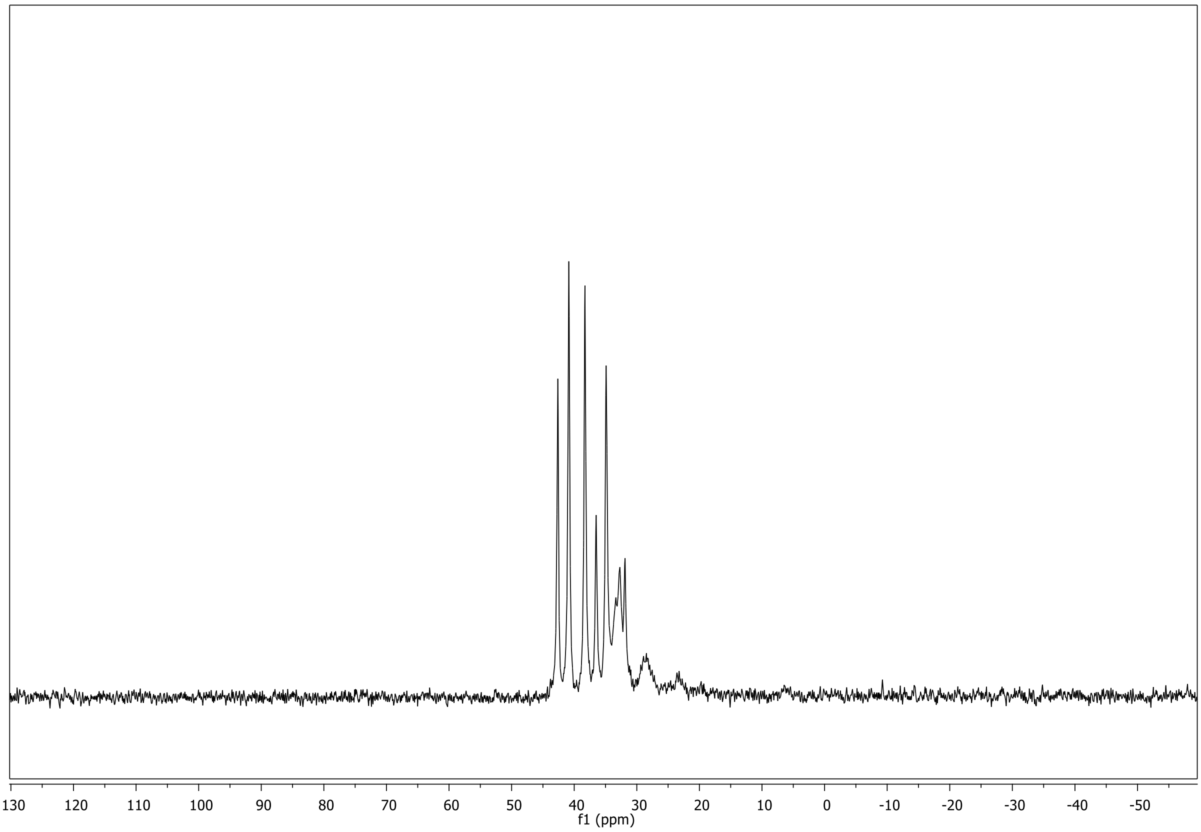

Figure S16. $^{31}P\{^1H\}$ NMR spectrum (CD_2Cl_2) at 193 K for complex **C1c**.

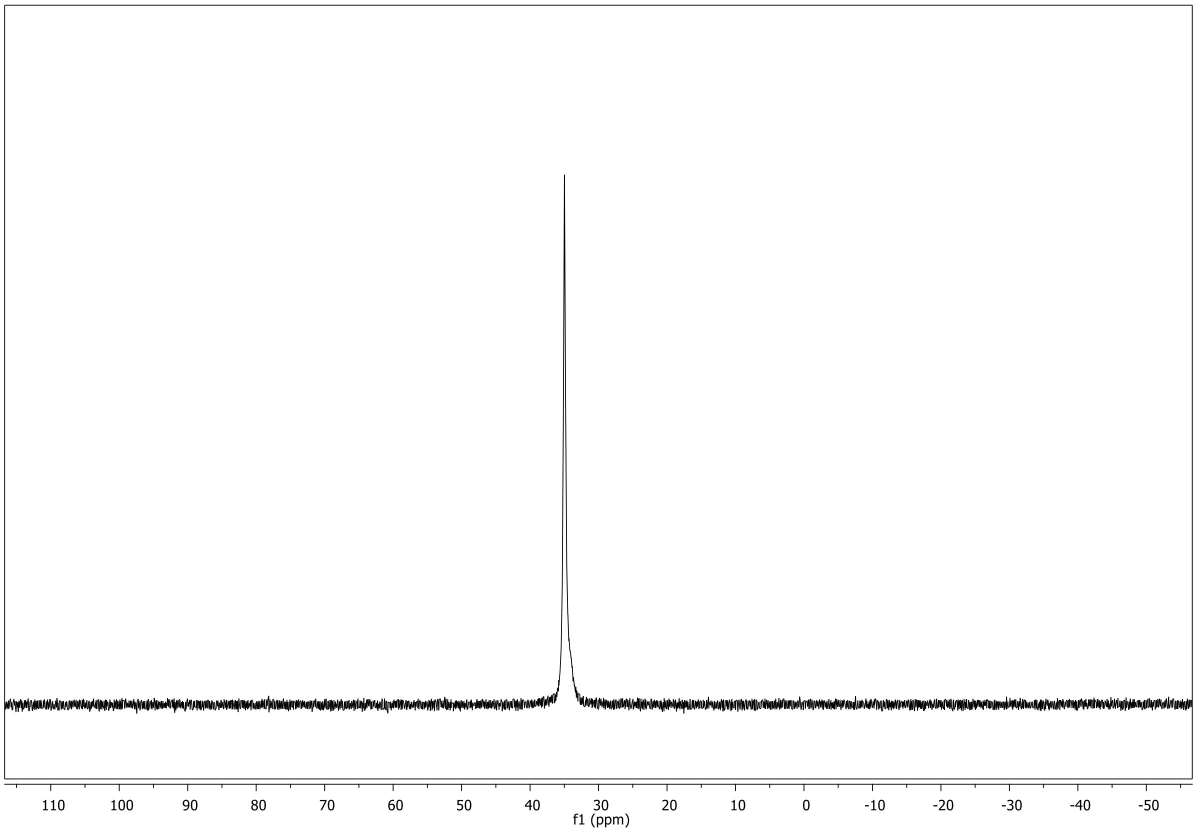

Figure S17. HSQC and COSY NMR spectra of **C2c** to explain the correlation among protons and proton-carbon on C1 and C2.

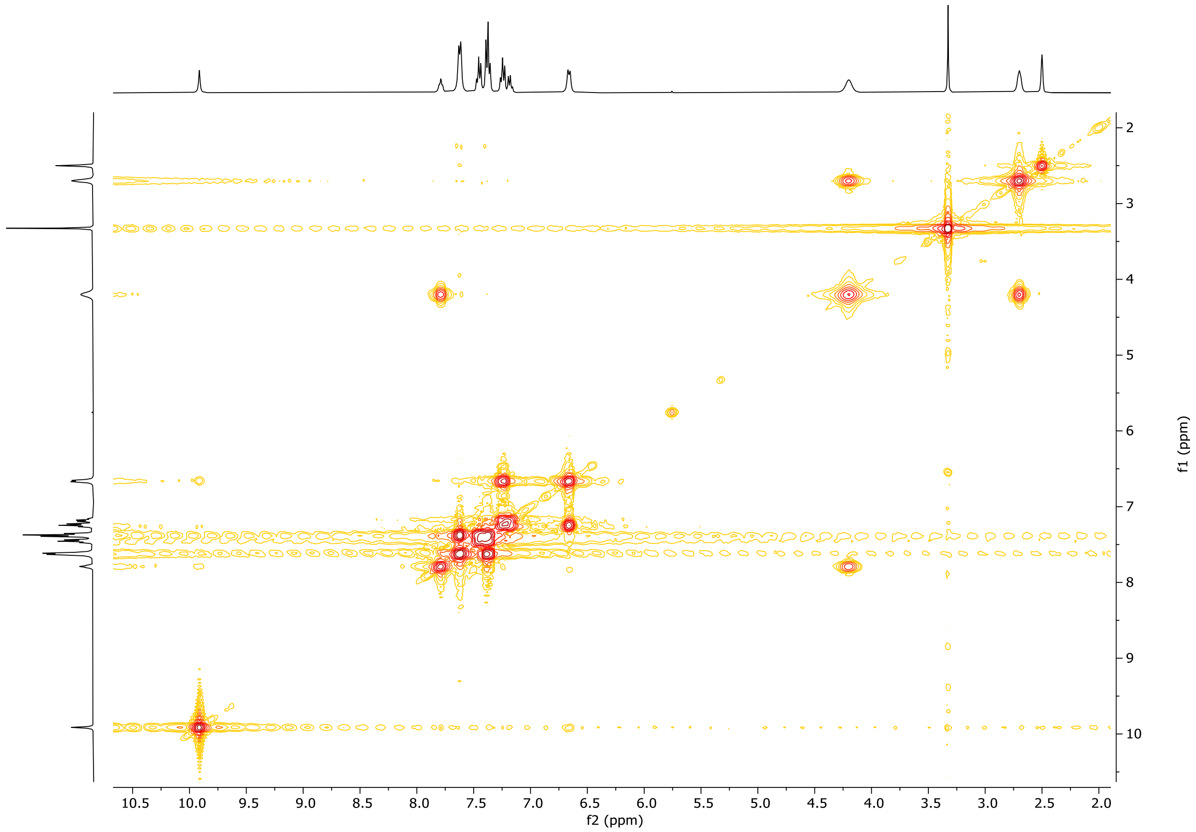

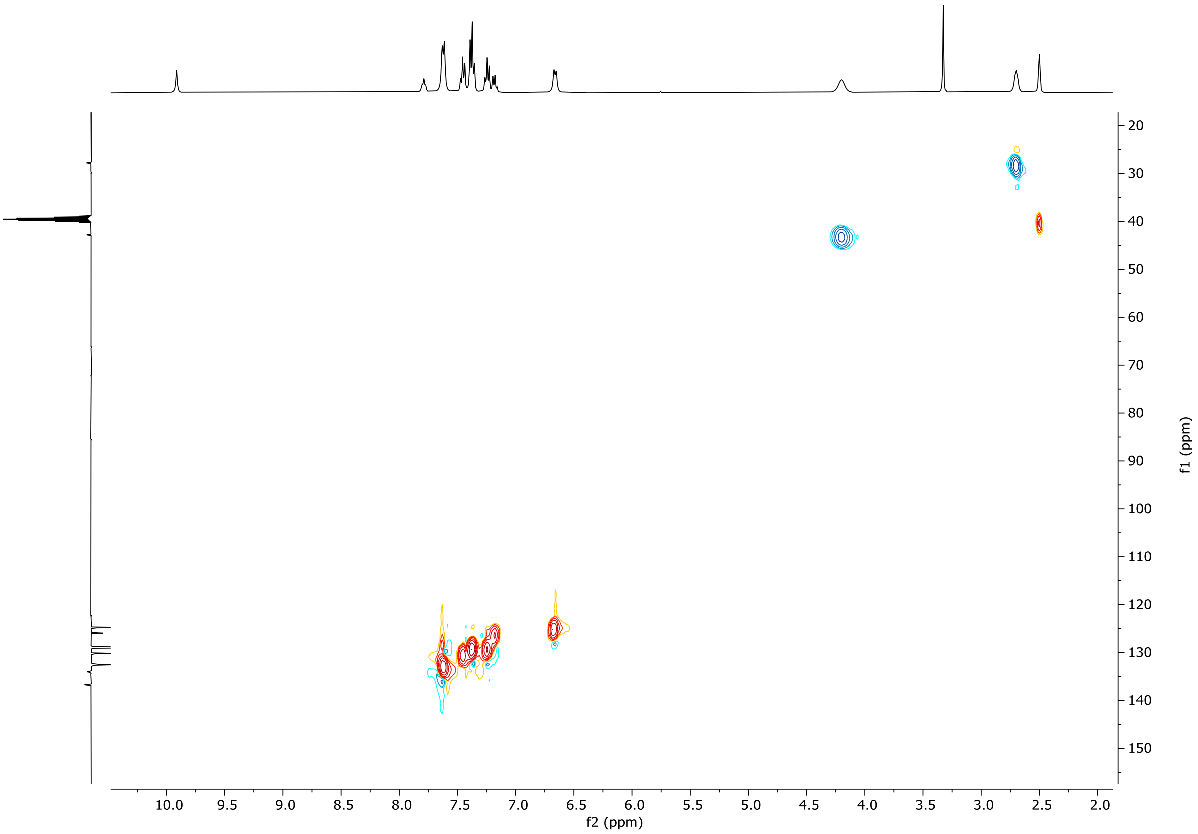

Explanation:

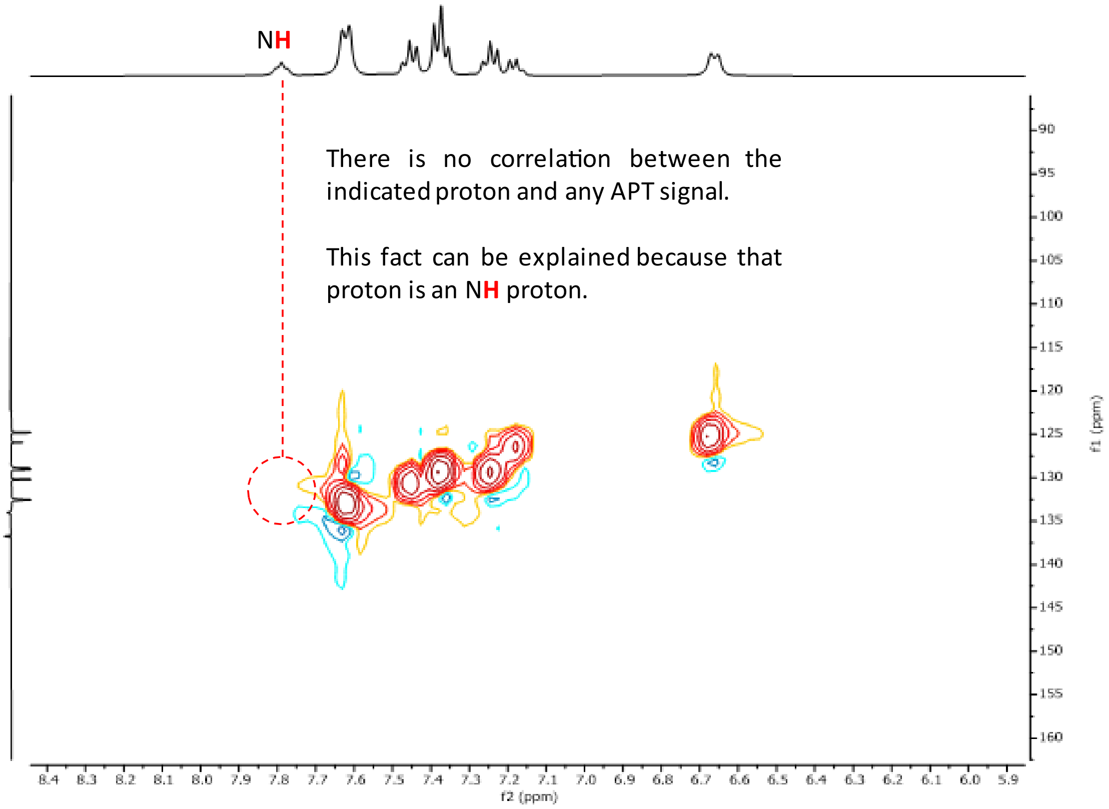

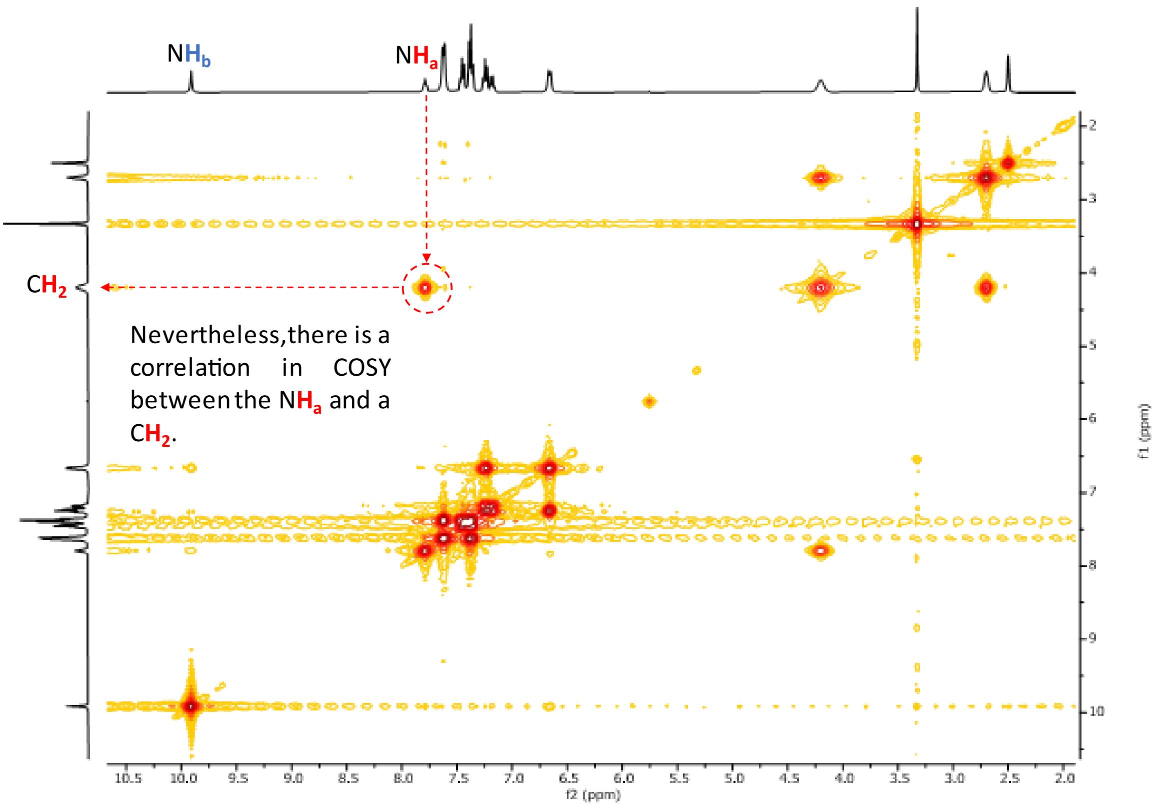

NHb
NHa
CH2
Nevertheless, there is a correlation in COSY between the NHa and a CH2.
f2 (ppm)
f1 (ppm)

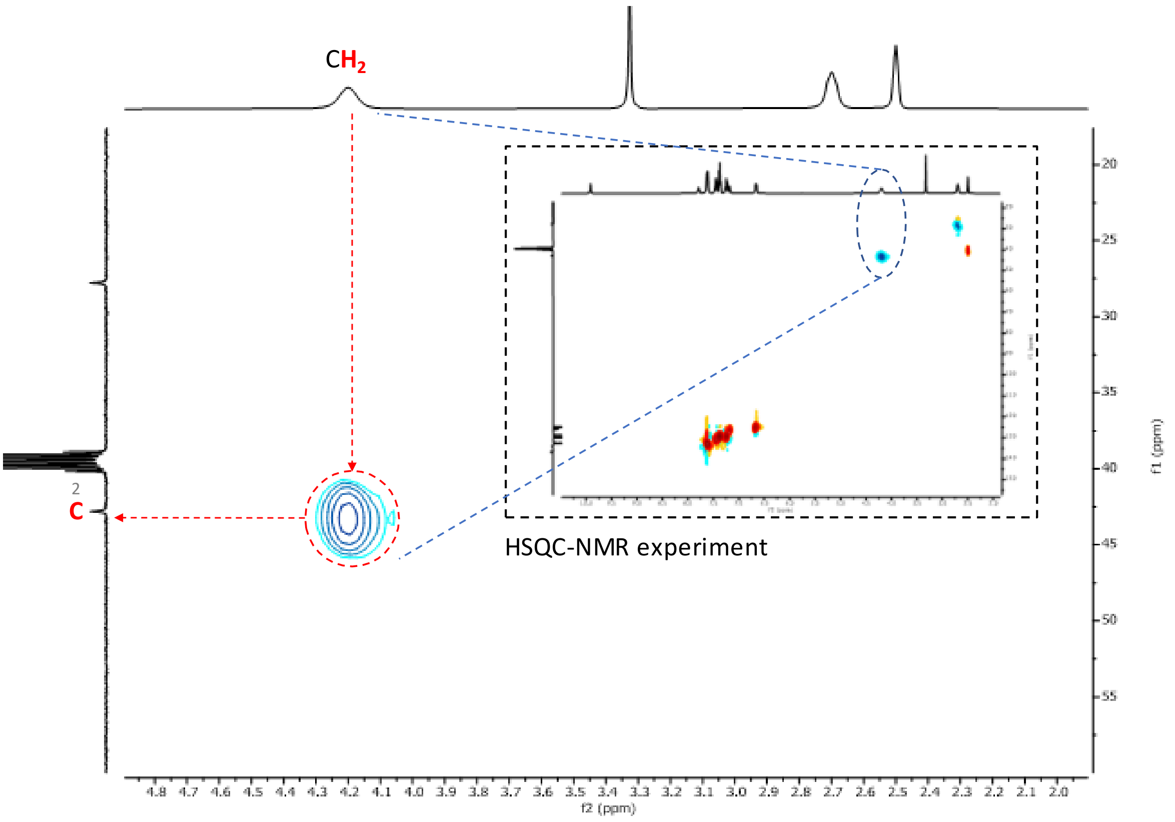

CH2
2
C
HSQC-NMR experiment
f2 (ppm)
f1 (ppm)

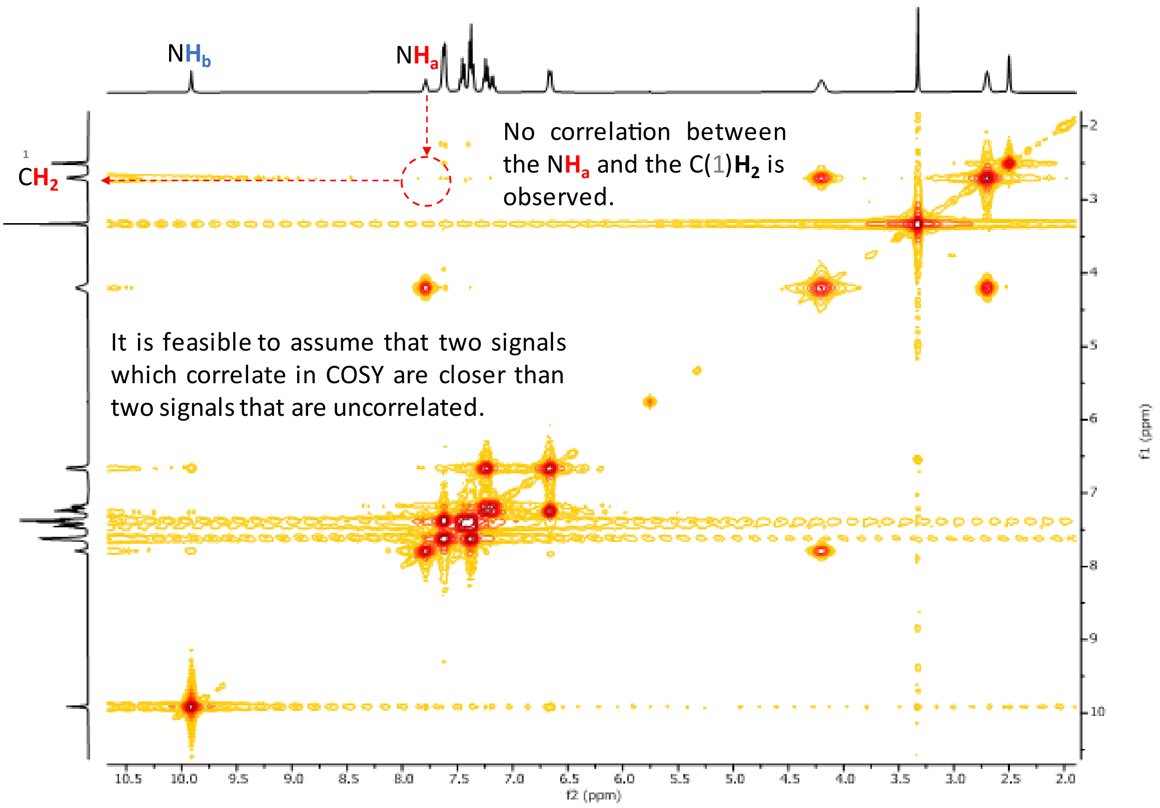

Figure S18. HSQC and COSY NMR spectra of **C2b** to explain the correlation among protons and proton-carbon on C1 and C2.

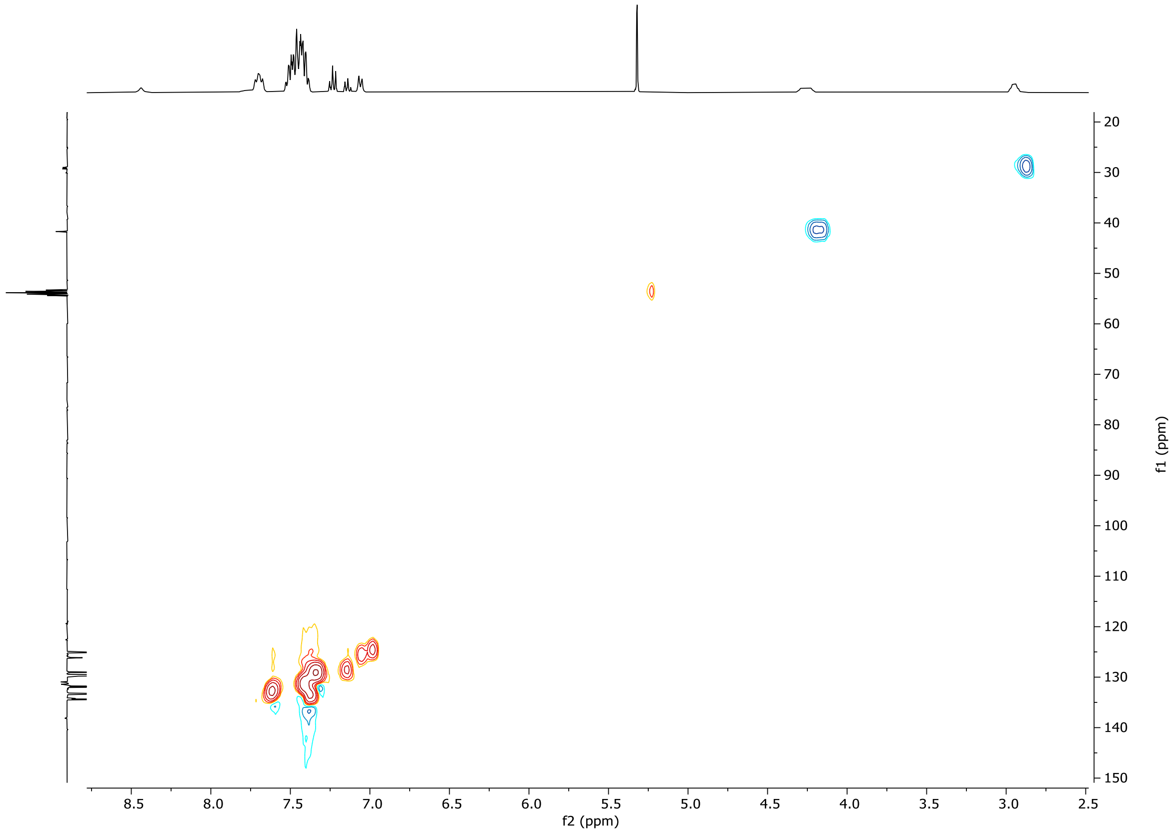

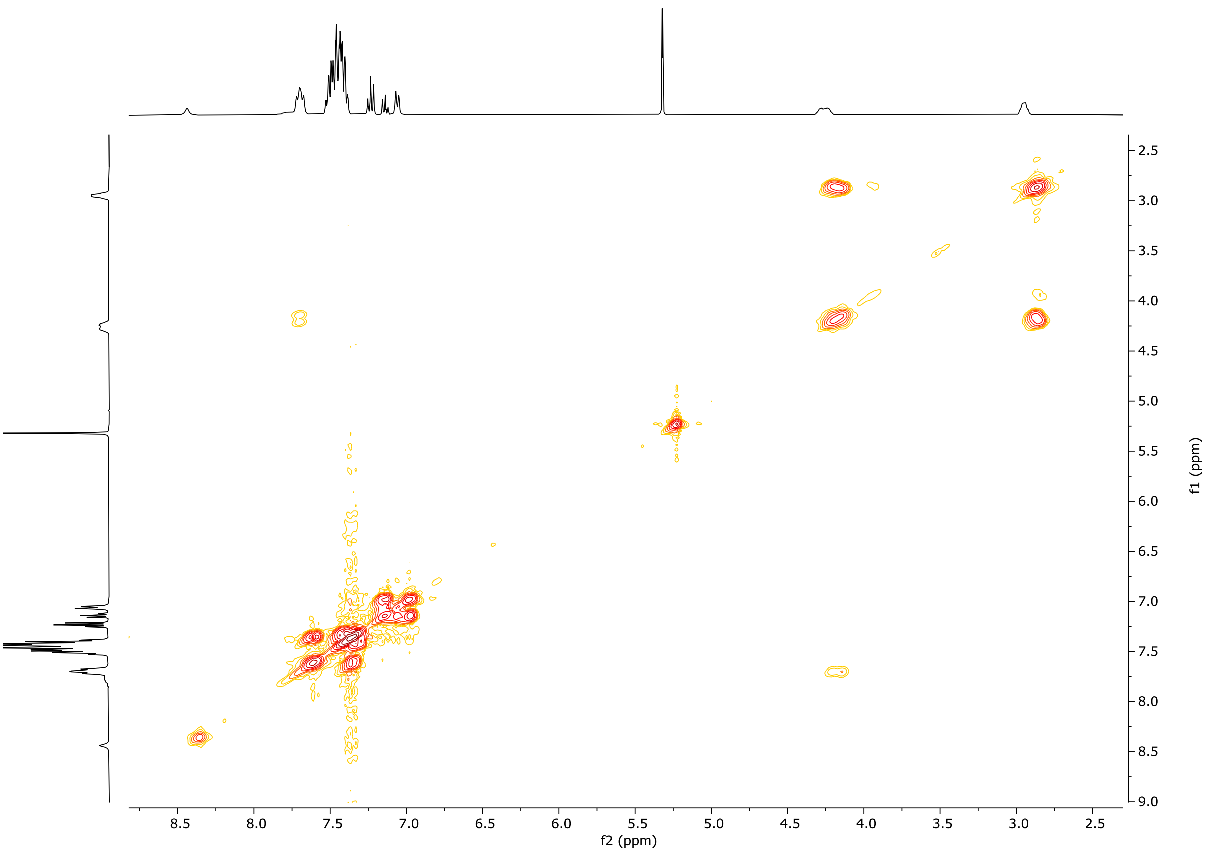

Figure S19. ESI-mass for all compounds

T1

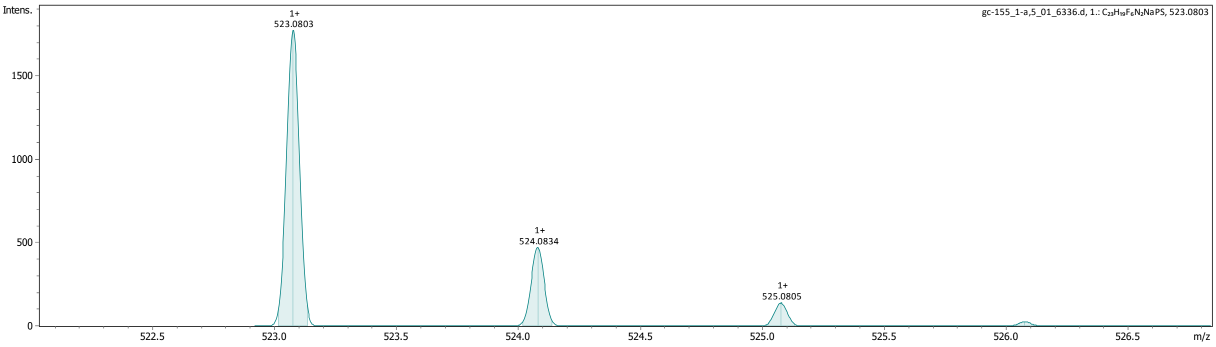

C1a

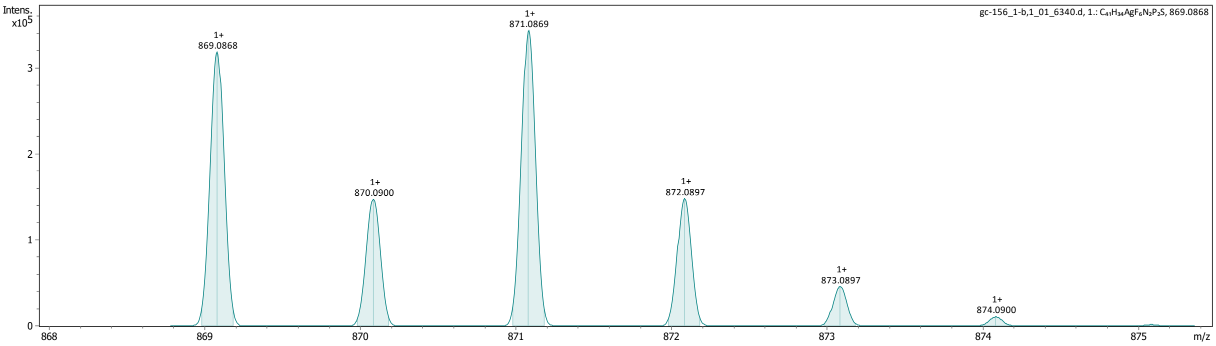

C1b

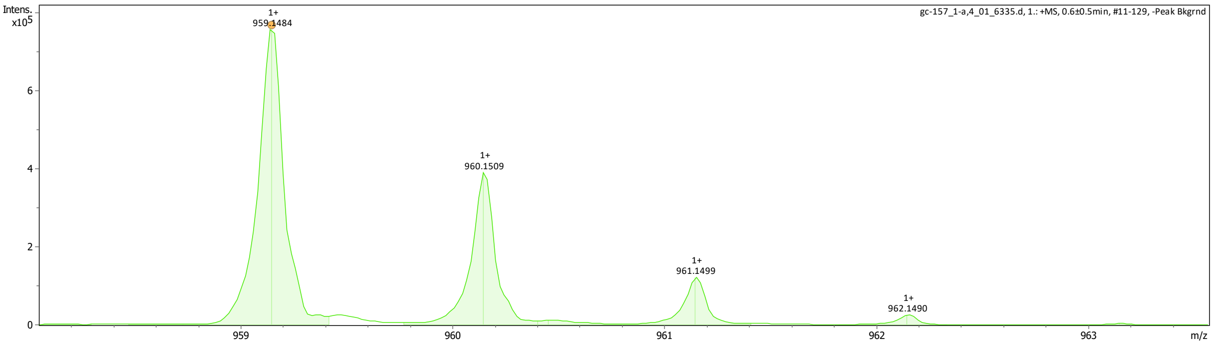

C1c

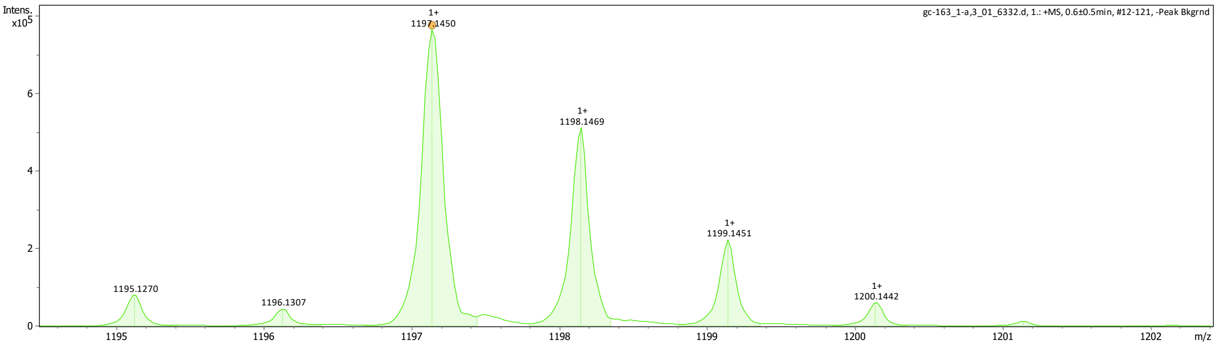

C1d

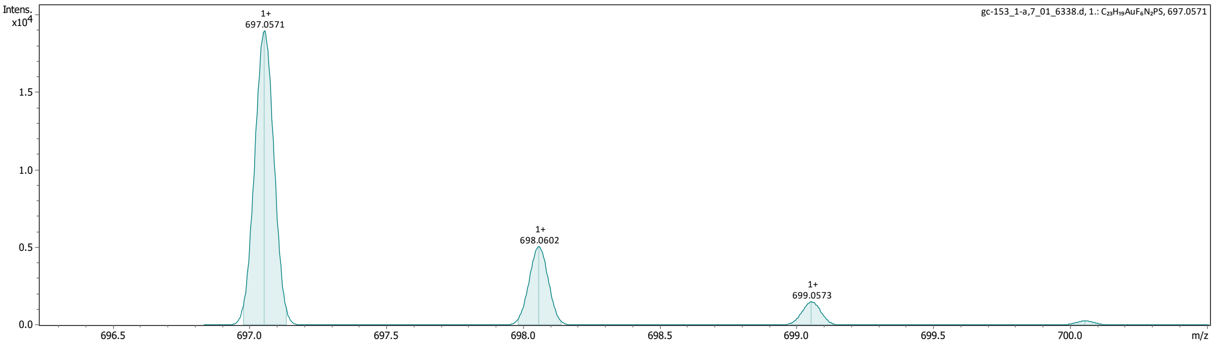

C1e

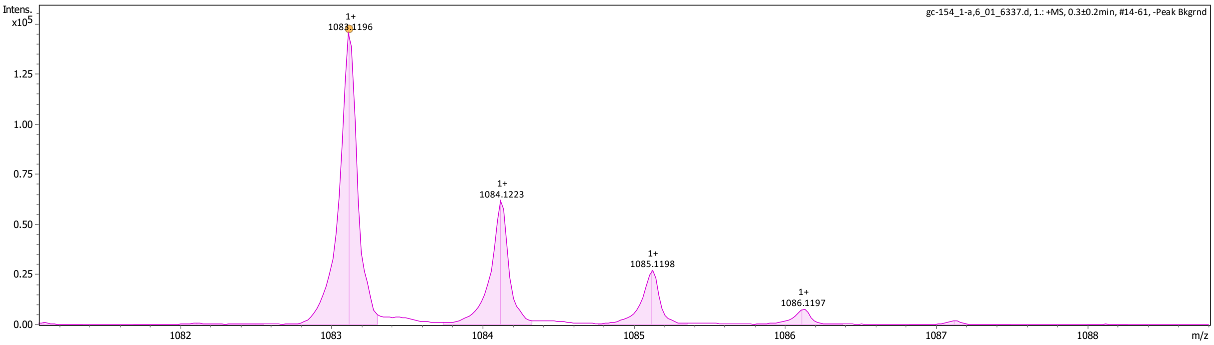

T2

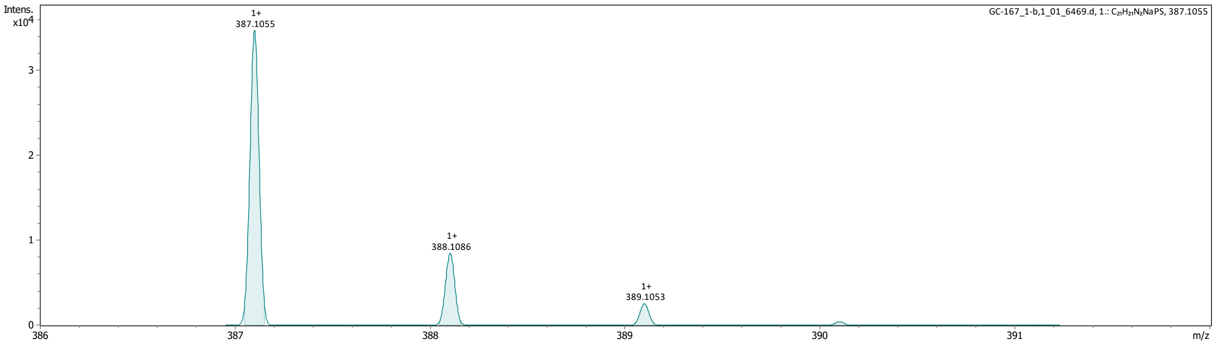

C2a

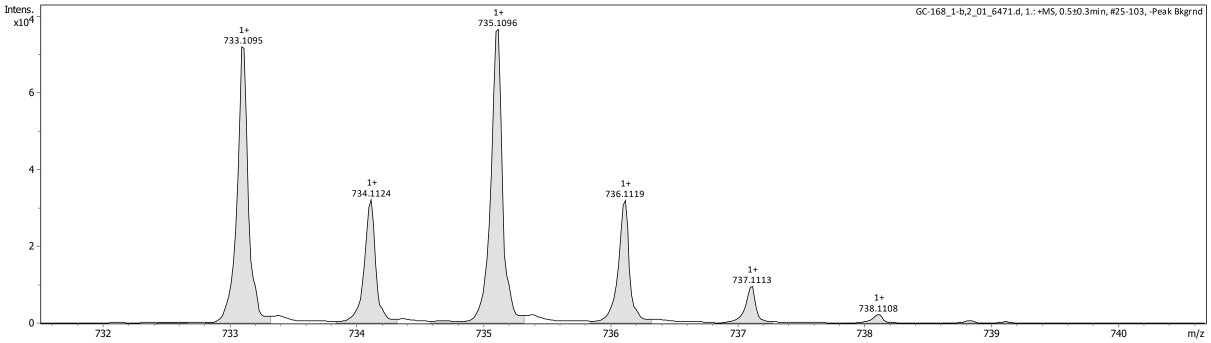

C2b

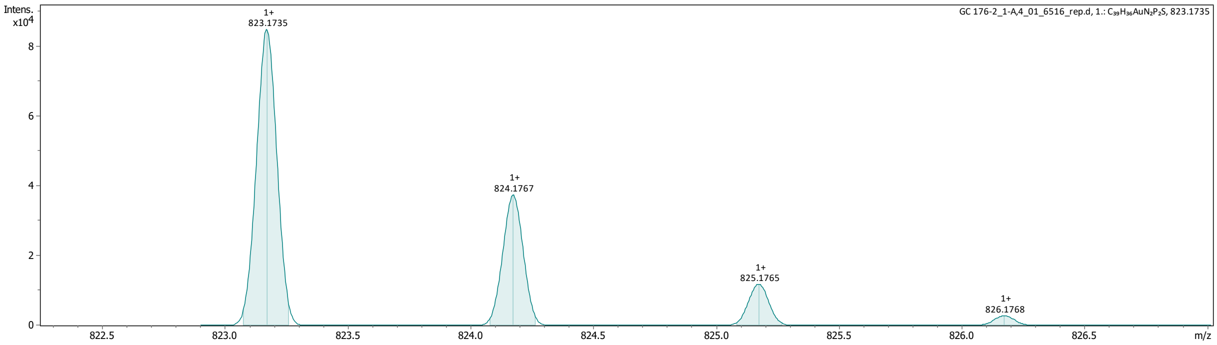

C2c

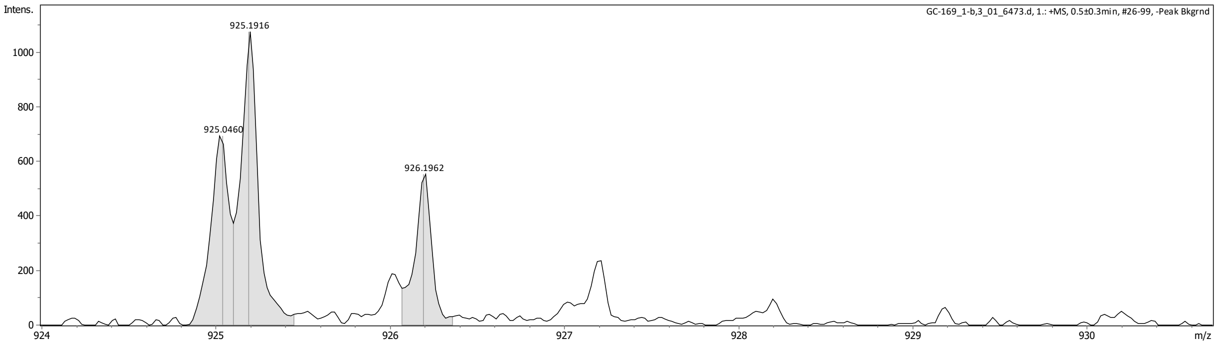

Supplement: Supplementary file 1 [file molecules-26-06891-s001.zip › molecules-1454717-supplementary.pdf]
